# Directing Near‐Perfect (001) Orientation in Wafer‐Scale BiOI Thin Films via Halide‐Mediated van der Waals Stacking

**DOI:** 10.1002/adma.74729

**Published:** 2026-09-23

**Authors:** Gabriel Aygur, Ashakiran Maibam, Hayden Tuohey, Tony Nguyen, Mei Xian Low, Sumeet Walia, Zijun (June) Yong, Ravichandar Babarao, Daniel Gomez, Enrico Della Gaspera, Joel van Embden

**Affiliations:** ^1^ School of Science RMIT University Melbourne Victoria Australia; ^2^ School of Engineering RMIT University Melbourne Victoria Australia

**Keywords:** orientation, oxyhalide, oxyiodide, photodetector

## Abstract

Controlling crystallographic orientation and facet exposure in 2D bismuth oxyhalide materials is critical for numerous applications, including efficient (photo)catalysis and enhanced charge transport. Oriented bismuth oxyhalide thin films are of strategic importance not only for their intrinsic properties but also as a gateway to many other valuable materials via their ability to undergo facile topotactic reactions. To progress the numerous applications linked to this family of materials this work identifies, for the first time, the mechanism regulating crystal orientation in bismuth oxyhalide (BiOI) thin films. Detailed experiments, backed by density functional theory (DFT) calculations, reveal the pivotal role of iodine (halide) anions to modify the van der Waals stacking energies required to direct orientation. Using our methodology, we demonstrate (001) BiOI thin films on commercially relevant wafer scales with unprecedented (001) crystallographic alignment—addressing the key challenge of scalability. To probe structure‐function relations, we fabricate BiOI photodetectors. Compared to unoriented devices, our (001)‐oriented films demonstrate vastly increased operational stability, fewer trap states, ON/OFF signal switching up to 10^5^, low (pA) noise levels, and millisecond response times. Our work can be used as a general methodology to control orientations in BiOX and related chalcohalide systems.

## Graphical abstract

Our work introduces a new framework that fundamentally changes how orientation in bismuth oxyhalide systems is approached. Comprehensive experiments, backed by theoretical models, reveal the pivotal role of halide anions to modify the van der Waals stacking energies required to direct orientation. Our method enables the fabrication of near perfect (001)‐oriented BiOI thin films on a multi‐inch wafer scale.

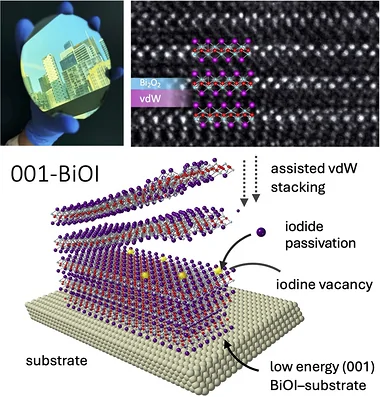

## 1 Introduction

Tailoring the spatial orientation of materials with low‐dimensional (1D or 2D) crystal structures enables directional modulation of charge transport and permits the deliberate exposure of specific crystal facets to enhance important (photo)catalytic reactions. Bismuth chalcohalide semiconductors are currently at the forefront of novel low‐dimensional materials [1, 2, 3, 4, 5]. They comprise a diverse class of V–VI–VII ternary 2D and 1D semiconductors of the general formula BiYX, where Y is a chalcogen and X is a halogen. Their ns^2^ electron configuration, high defect tolerance, and tunable optical band gap (dependent upon the chalcogen and halogen) have encouraged their implementation across a broad range of optoelectronic applications [5, 6, 7, 8, 9, 10, 11]. Likewise, their facet‐dependent reactivity has been exploited to catalyze high‐value chemical reactions [12, 13, 14, 15, 16].

A particularly influential material in this class is bismuth oxyiodide (BiOI). BiOI is an *n*‐type semiconductor [17, 18], possessing an indirect optical band gap between 1.8 and 2.0 eV [18, 19]. BiOI has a long history as a photocatalyst [20, 21, 22, 23, 24] and environment remediation agent [23, 25, 26, 27, 28]. The synthesis of BiOI may be accomplished by a diverse range of strategies. These include vacuum [29, 30, 31, 32, 33] and mist‐based [18] physical and chemical vapor deposition (PVD/CVD) as well as wet chemical methods, which are typically hydrothermal/solvothermal [34, 35, 36], although various other methods have also been developed [37, 38, 39, 40, 41]. In recent years its application has expanded to optoelectronics, finding use as a solar cell absorber layer [19, 29, 30, 31, 42] as well as for photodetection [32] and x‐ray detection [43]. The success of BiOI (and analogous oxyhalides) may in large part be attributed to their strong structure‐function relationship. The BiOI crystal structure is formed from covalently bonded [Bi_2_O_2_]^2+^ slabs interleaved by iodide layers, with weak van der Waals (vdW) (iodine‐iodine) interactions permitting the stacking of each covalent slab [44, 45]. The 2D crystal structure induces a dipolar electric field along the covalent [001] axis that generates strong anisotropic properties, wherein charge transport is facilitated within the covalent plane and charge separation promoted via the internal field [45, 46]. This phenomenon manifests as enhanced catalytic activity localized on the (001) facet of BiOI and related isostructural bismuth oxyhalides [47, 48, 49, 50]. Within optoelectronic applications, oriented BiOI thin‐film architectures are necessary for realizing optimal photovoltaic performance [30]. Likewise, bismuth oxyhalides with a (001) orientation have proven to be essential starting materials to enhance the electrocatalysis of CO_2_ to liquid fuels [51, 52]. Numerous other examples on water splitting and ammonia generation directly evidence the strong link between crystallographic orientation (controlled crystal facet exposure) and optimal material performance [14, 53].

Despite these well‐documented advantages, the ability to control crystal orientation in bismuth oxyhalides remains substantially underdeveloped. Most existing reports show only weak preferential orientation, with levels only slightly exceeding randomly oriented powder samples. Reports demonstrating significant control over crystal orientation are few and have exclusively focused on BiOI. Regulating the precursor vapor pressure/substrate temperature within vacuum‐based CVD systems constitutes the only presently available method for producing oriented BiOI thin films [30]. However, the orientation control strategy used in these works was derived empirically without addressing the underlying cause of crystallographic alignment at the atomic level. Consequently, the chemical mechanism controlling orientation in oxyhalide materials remains unsolved.

Given the prevalence of applications that depend upon controlled facet exposure and orientation, it is of great value to uncover the mechanism that drives orientation in the oxyhalide family of materials. This achievement would constitute a substantial breakthrough, enable the preparation of highly oriented samples, and provide the morphological control required to further advance applications.

Herein, we present the first report on oriented BiOI thin films fabricated via a solution‐based method. Our method is scalable, can be conducted at room‐temperature, and produces vastly superior crystal orientation compared to benchmark high‐temperature vacuum depositions. By generating BiOI within a carefully controlled iodine‐rich environment, we were able to achieve near‐perfect (001) orientation. Through a series of in‐depth experiments, we identify that halide (iodide) anions play a critical role in modifying the vdW interaction energies that regulate crystallographic orientation in BiOI. These results are supported by density functional theory (DFT) calculations, which unveil the strong connection between both surface and vdW stacking energies and facet iodine density. We further demonstrate that our method can be scaled to fabricate (001) BiOI thin films spanning a 4‐inch wafer while retaining orientational uniformity. Finally, to characterize the optoelectronic properties of our films and to probe the structure‐function relationship between randomly oriented and well‐ordered (001)‐oriented BiOI, photodetector devices (PDs) are fabricated. Our (001)‐oriented devices show vastly improved stability with high (∼10^5^) ON/OFF signal switching, millisecond response times, and outstanding responsivities and device sensitivity (∼10^12^ Jones), rivaling previously reported high‐temperature vacuum‐deposited bismuth oxyhalide single‐nanosheet PDs. Overall, our results provide a platform to deliver controlled orientations across bismuth oxyhalide and chalcohalide systems via halide anion modified vdW interactions.

## 2 Results and Discussion

BiOI thin films were fabricated via the direct hydrolysis of bismuth(III) iodide (BiI_3_), as outlined in our earlier seminal works [42, 54]. Briefly, BiI_3_ powder is dissolved in anhydrous tetrahydrofuran (THF) and spin coated onto a target substrate to yield a template BiI_3_ thin film, which is then hydrolyzed to form BiOI (see Experimental for details). When converting BiI_3_ to BiOI a distinct color change is observed from dark brown to yellow/orange. Advantageously, the hydrolysis‐based conversion into BiOI produces carbon free films with the only by‐product being hydroiodic acid (HI)—as per (Reaction 1) (Figure S1). This method may be coupled with a layer‐by‐layer technique to achieve BiOI thin films of a desired thickness (Figure S2). The hydrolysis method differs from BiOI (thin film) formation via vacuum CVD‐ or PVD‐based methods [19, 29, 30, 31, 32], wherein molecular oxygen is used instead of water with the liberation of iodine gas (Reaction 2):

(1) $$\mathrm{Bi}{I_{3}}_{\left(s\right)}+H_{2}O_{\left(l\right)}⇌\mathrm{BiO}I_{\left(s\right)}+2HI_{\left(\mathrm{aq}\right)}$$

(2) $$\mathrm{Bi}{I_{3}}_{\left(s\right)}+1/2{O_{2}}_{\left(g\right)}⇌\mathrm{BiO}I_{\left(s\right)}+{I_{2}}_{\left(g\right)}$$

To establish a solid foundation for this study, our BiOI thin films were subjected to rigorous preliminary characterization. The as‐prepared BiOI thin films are phase‐pure (without any residual BiI_3_ reflections) and match the tetragonal (P4/nmm) BiOI (ICDD# 10‐0445) crystal structure (Figure S3). The corresponding BiOI thin films reliably have complete surface coverage and typically consist of randomly oriented 2D‐nanoflakes (∼10‐15 nm thick). A bulk indirect band gap of 1.91 ± 0.02 eV was estimated from thick BiOI films (Figure S4) and agrees well with current literature values [19, 37, 55]. Absorbance spectra coupled with ultraviolet photoelectron spectroscopy (UPS) were conducted to define the flat band structure of BiOI. The Fermi level, valence band maximum (VBM), and conduction band minimum (CBM) position were calculated at −5.14, −6.53, and −4.58 eV (relative to vacuum), respectively (Figure S5), and confirm our BiOI thin films are *n*‐type [17, 18].

To understand crystallographic orientation in 2D thin film materials, it is important to appreciate the orientation of dominant crystal planes relative to the 2D structure. BiOI is a 2D vdW crystal with a stacked crystallographic structure composed of [Bi_2_O_2_]^2+^ slabs interleaved by iodine atoms to form vdW monolayers. Figure 1a illustrates the BiOI crystal structure viewed along the [110] direction, with the addition of the five major crystallographic planes ((001), (102), (110), (200), and (212)). BiOI grows in a nanoflake morphology with lateral dimensions extending along the covalently bonded *a*/*b*‐axis directions and a height (*c*‐axis) spanning the vdW stacked layers. These nanoflakes may orient themselves in many ways relative to a substrate surface. For typical BiOI thin films, these flakes are randomly positioned and consequently display XRD patterns that closely resemble powder patterns. In the case of a (001)‐oriented BiOI thin film, the 2D monolayers (vdW layers) are aligned parallel to the substrate surface. Conversely, a (hk0)‐oriented BiOI thin film features monolayers that stand vertically, extending outward from the substrate. Analysis of film texture enables effective comparison of films with different orientations, wherein the texture coefficient (P) provides a measure of film orientation in each crystallographic direction (relative to the substrate). For this work we consider the 5 main reflections for BiOI as outlined above. As such, P(*hkl*)= 5 for a given crystal plane indicates perfect orientation in the designated *hkl* direction, while P(*hkl*)= 1 indicates random orientation. Further details on texture coefficient calculations are provided in the Supporting Information.

**FIGURE 1:**
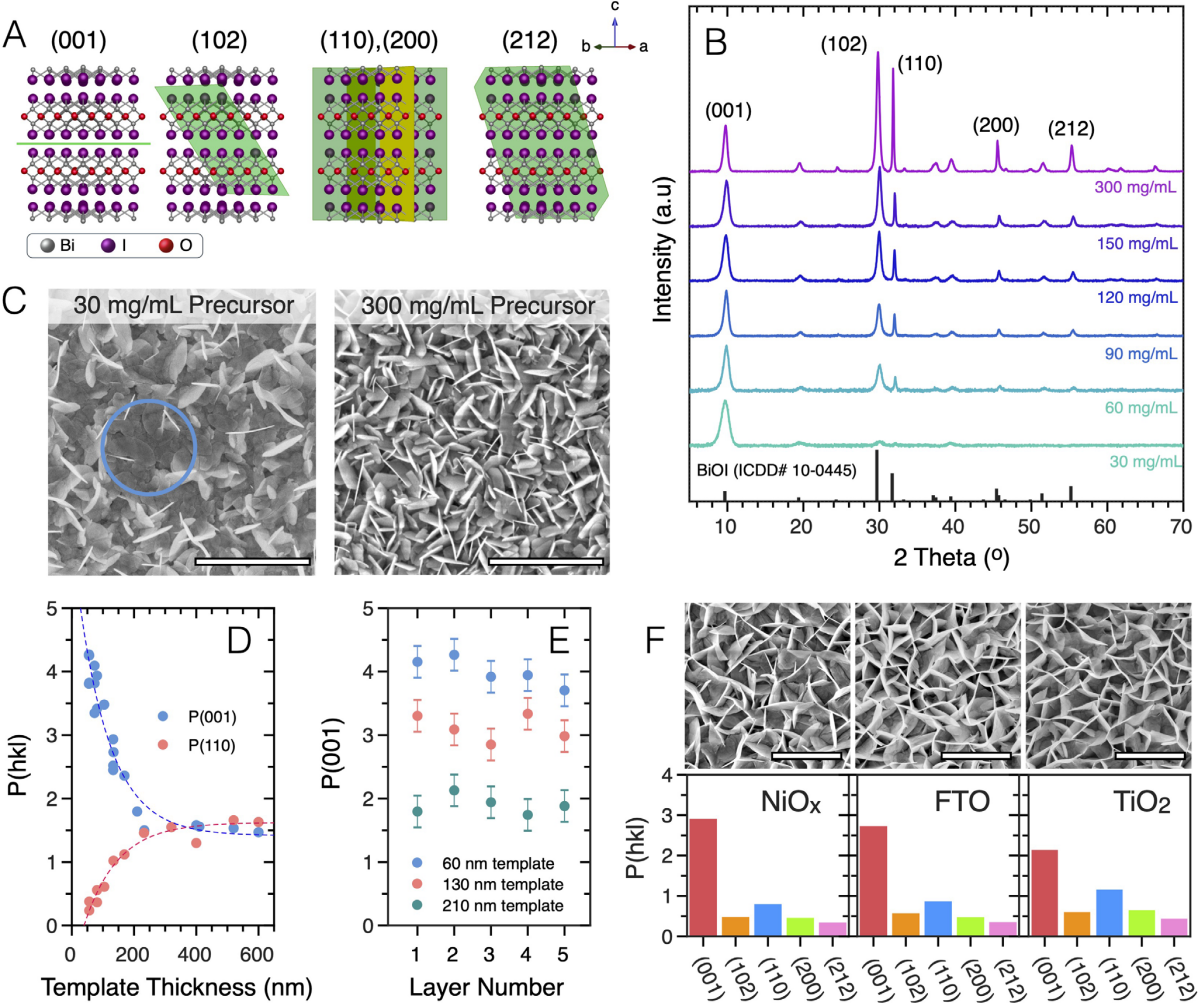
(a) BiOI crystal showing the various key lattice planes. (b) XRD patterns of 3L BiOI films fabricated from different [BiI_3_] precursors. (c) SEM images of 3L BiOI from low (30 mg/mL) and high (300 mg/mL) precursor concentrations. (d) BiOI texture coefficients for varying initial BiI_3_ template thicknesses. (e) BiOI P(001) for various template thicknesses, with sequentially increased layer number. (f) SEM and corresponding texture coefficients for 3L BiOI (100 mg/mL) prepared on a variety of common substrates. All scale bars are 1 µm.

Preliminary investigations into BiOI thin film formation were conducted to assess the effect of key reaction parameters on the properties of the resultant BiOI films. Figure 1b shows a series of XRD patterns of three‐layer (3L) BiOI films converted from BiI_3_ template films cast from BiI_3_/THF precursor concentrations between 30 and 300 mg/mL. For clarity, XRD peak intensities have been normalized to the (001) diffraction peak. Interestingly, at the lowest precursor concentration (30 mg/mL), BiOI is preferentially (001)‐oriented, showing a large diffraction peak at ∼9.7°. As the precursor concentration is gradually increased, the BiOI film adopts a more random orientation, as indicated by a massive increase in the (102), (110), (200), and (212) diffraction peaks relative to (001). This shift toward a random orientation is directly reflected in the bulk morphology of the thin films. Figure 1c shows top‐down SEM images obtained at low (30 mg/mL) and high (300 mg/mL) precursor concentrations. BiOI generated from a low concentration precursor displays a significant portion of flat regions (blue circle), with few vertically aligned nanoflakes. In contrast, high precursor concentrations produce BiOI films with a dense array of randomly oriented flakes (Figure S6).

To explain these orientational changes, we first correlated precursor concentration to the resulting template film thickness [54]. When key texture coefficients are plotted against template thickness, a clear trend emerges. Figure 1d displays the calculated texture coefficients of the two major orthogonal planes—P(001) (blue dots) and P(110) (red dots)—for single‐layer BiOI as a function of BiI_3_ template film thickness. Thin template films (∼50 nm) produce preferentially (001)‐oriented BiOI with P(001) = 4.3 (out of 5), while (110) is highly suppressed (P(110) << 1). As template thickness increases, P(001) declines and the films steadily approach a random orientation with all texture coefficients converging to P ∼ 1. These data uncover the strong correlation between template film thickness and the resultant BiOI thin film orientation post‐hydrolysis. Notably, we found that the orientation of BiI_3_ thin films varied slightly between sample batches and was sensitive to processing conditions such as atmospheric moisture, solvent (THF) purity, and BiI_3_ powder quality. Nevertheless, targeted experiments revealed that the orientation of BiOI is not correlated to the orientation of the BiI_3_ template film, ruling out topotactic conversion (Figure S7).

Studies on multilayer films were conducted to confirm that the observed changes to texture were not a measurement artifact of overall film thickness. Figure 1e shows a plot of P(001) as a function of layer number for three select BiI_3_ template thicknesses. These data show the invariability of texture to changes in BiOI film thickness, which remains relatively constant for each template used. Finally, it is well known that substrate type may also strongly affect the morphology of thin film samples [56, 57]. As initial texture‐based characterizations were conducted on glass/NiO_x_ substrates (as proven to be highly useful for optoelectronic device application) [19, 42], we assessed the persistence of orientational effects on other substrates (Figure 1f). The film morphology and P(*hkl)* values were found to be similar on NiO_x_, FTO, and TiO_2_ substrates. Broader studies showed that the BiOI nanoflake morphology was also nearly identical on Si, SiO_2_, PEDOT:PSS, and SnO_2_, and CuSCN (Figure S8). The insensitivity of BiOI morphology to substrate type is advantageous, establishing that orientations achieved using our hydrolysis method translate across a wide range of common substrates, thereby permitting greater flexibility in application (device) architectures.

Collectively, these results confirm that BiI_3_ template film thickness is a decisive factor governing the morphological evolution of BiOI during hydrolysis and can be used as a tool to guide crystallographic orientation. Film conversion begins at the BiI_3_/water interface and proceeds downward to the substrate surface, generating BiOI nanoflakes. We hypothesize that for thick template films, once conversion has reached the substrate, randomly oriented nanoflakes have been generated. However, for very thin films a larger portion of the conversion takes place near or at the substrate surface. It has been shown that the (001) iodine terminated facet of BiOI is the lowest energy facet [58, 59, 60]. The provision of a low‐energy substrate interaction ultimately supports (001)‐preferred orientations in thinner films.

These prior investigations provide the required foundation upon which to build a deeper understanding of BiOI growth in solution. During the conversion (hydrolysis) of BiI_3_, hydroiodic acid (HI) is liberated from the films as BiOI is formed, as outlined previously in (Reaction 1). This hydrolysis is a heterogeneous equilibrium reaction wherein the yield and formation rate of BiOI may be manipulated using HI. Iodine is crucial to the 2D layered structure of BiOI, residing at the terminal faces of the vdW stacked [Bi_2_O_2_]^2+^ slabs. These iodine atoms are those that form the (001) crystal facet of BiOI, which may be either iodine‐rich or iodine‐poor. As such, we expect the iodide ion (I^–^) to play a pivotal role in the crystallization process of BiOI and have the potential to exert morphological/orientational effects through manipulation of the vdW stacking energies.

To explore the effect of iodide, varying amounts of HI were added to the conversion bath. Early investigations revealed that high [HI] resulted in immediate dissolution of BiI_3_ thin films upon immersion in the bath. However, lower concentrations were able to readily yield BiOI. Figure 2a shows raw (unscaled) XRD patterns of BiOI prepared from various [HI] in the bath. Increasing [HI] from 0 to 7 mm results in a massive concomitant increase in signal intensity (over an order of magnitude) and a significant decrease in the full width half maximum (FWHM) of the diffraction peaks—indicating vastly improved crystallinity (Figure S9). Even more striking is that the films are seen to become exceedingly (001)‐oriented. A significant decrease in both (102) and (110) reflection intensities is observed, while the (001) reflection becomes prominent. The (001) orientation is highest at [HI] = 7 mm, wherein only periodic {00*l*} reflections can be readily observed in the sample XRD. We note that [HI] is limited to ∼7 mm, and [HI] > 10 mm yielded inhomogeneous films due to corrosion (Figure S10). At ∼10 mm the forward reaction rate to produce BiOI is equivalent to the dissolution rate (back reaction rate) to BiI_3_. Close to the maximum permissible [HI], the formation rate of BiOI is slow, and the greatest crystallinity and crystallographic alignment occur. Notably, slowing of the conversion time is significant enough to be seen visually by the time taken for the films to change color from brown (BiI_3_) to orange (BiOI)—increasing in duration from ∼10 s to ∼1 min. Attempts to further slow the growth rate by lowering the bath temperature to as low as −10°C in water/ethylene glycol mixtures had no discernible effect on crystallographic orientation.

**FIGURE 2:**
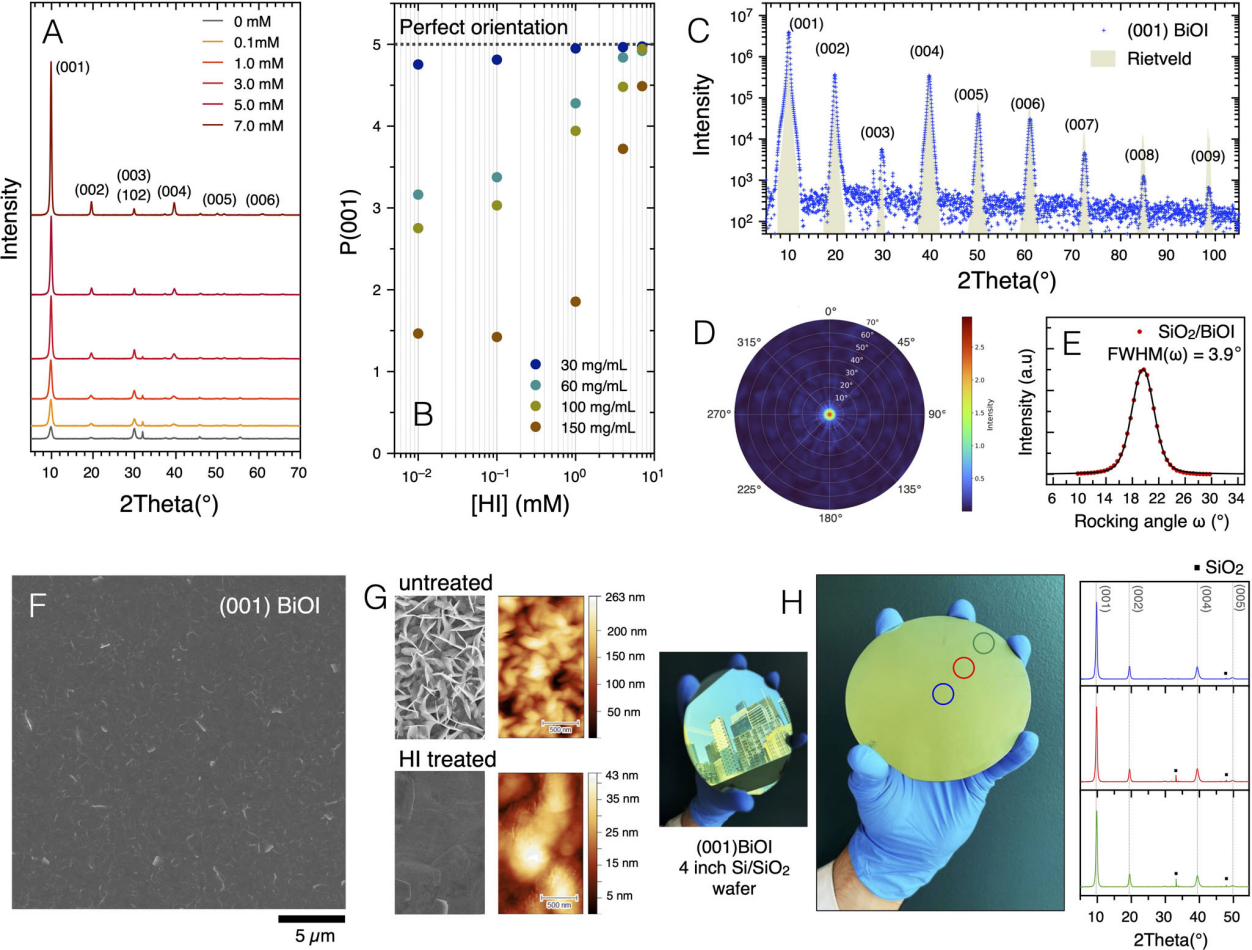
(a) XRD patterns of 3L BiOI (100 mg/mL) prepared in varying [HI]. (b) P(001) values for 3L BiOI of different [BiI_3_] precursors, prepared in varying [HI]. (c) Semilogarithmic wide‐angle 1D XRD pattern of 001‐BiOI (7 mM HI bath). (d) Pole figure (004) of a 001‐BiOI on Si/SiO_2_. (e) BiOI (004) Rocking Curve. (f) Low‐magnification 25 × 25 µm area SEM image of 001‐BiOI. (g) Approximately 2 × 1 µm area SEM images of standard (top) and 001‐BiOI (bottom) alongside their corresponding AFM images. (h) Digital images of 001‐BiOI on a 4‐inch Si/SiO_2_ wafer and the corresponding XRD patterns from various locations on the wafer surface (all patterns yielded (P(001) = 4.99).

Remarkably, the orienting effect of HI is prevalent regardless of the template film thickness (Figure 2b). Irrespective of the initial orientation, as [HI] increases to its optimal concentration, P(001) progressively increases toward P(001) = 5 (perfect orientation). Changes to film orientation are most pronounced in thick films, which begin with a near random orientation and increase to become highly (001)‐oriented at optimal [HI]. To demonstrate the versatility of the technique, 001‐BiOI films were successfully deposited on a variety of commonly employed device substrates (Figure S11). It is worth noting that this hydrohalic‐acid‐assisted approach could be extended to produce (001)‐oriented BiOBr films, although only modest improvements were observed because BiOBr naturally exhibits a strong preference for (001)‐oriented growth (Figure S12).

Calibrated elemental analysis was conducted via energy dispersive x‐ray spectroscopy (EDS) to determine whether the orienting effects were due to significant changes in iodine content. We found sequentially increasing amounts of iodine are present in more oriented samples. The stoichiometric excess of iodine peaks at ∼3.3% for 7 mm conversions, indicating that lattice iodine may be a key participant in the orienting process (Figure S13). Optoelectronic analysis revealed that the slight excess of iodine did not significantly alter the band structure, with (001)‐oriented BiOI displaying a slightly lower band gap and similar band positions (Fermi level) to standard converted BiOI (Figure S14).

Super‐oriented BiOI thin films may be achieved by a combination of thin template films and optimal [HI]. Figure 2c shows the 1D XRD diffractogram for 001‐BiOI, with the signal intensity plotted on a logarithmic scale to accentuate any minor diffractions not visible on a linear scale. Nine periodic {00*l*} reflections are observed, which match perfectly to the simulated (001) diffraction pattern for tetragonal BiOI (P4/nmm, space group 129) with almost zero contribution from any other crystal orientation (Figure S15). From this diffractogram, a P(001) = 4.996 was calculated, indicating near perfect (001) crystallographic alignment. For reference, state‐of‐the‐art BiOI thin films synthesized via CVD exhibit a scaled (001)/(110) intensity ratio of approximately 9 (extrapolated P(001) = 2.84), whereas our films achieve a significantly higher ratio of ∼7300 (P(001) = 4.99). The average P(001) values from the measurement of ∼20 optimal samples are 4.95 ± 0.044. The ability to manipulate BiOI orientation to this extent has not been possible until now and vastly surpasses benchmark BiOI generated from vacuum depositions [19, 30].

The crystallographic texture of the highly oriented 001‐BiOI films was quantified using pole figure and rocking‐curve measurements of the BiOI (004) reflection on an Si/SiO_2_ substrate. The pole figure exhibits a highly intense central pole with a rapidly decaying off‐axis intensity and minimal azimuthal variation, confirming a strong *c*‐axis fiber texture with the majority of crystallites aligned normal to the substrate surface (Figure 2d). The absence of pronounced azimuthal intensity maxima indicates that, although the crystallites exhibit strong preferential *c*‐axis alignment, they possess limited long‐range in‐plane orientational order and are therefore best described as a highly textured fiber structure rather than an epitaxial film. Consistent with the pole‐figure results, Voigt fitting of the corresponding BiOI (004) rocking curve yielded an FWHM of 3.9°, indicating a narrow crystallite tilt distribution and low mosaic spread (Figure 2e). This corresponds to a root‐mean‐square (RMS) tilt of approximately 1.7°, demonstrating that most crystallites are oriented within only a few degrees normal to the substrate. Comparative measurements of films deposited on the same substrate incorporating a thin (∼10 nm) NiOx interlayer showed a broader pole‐figure intensity distribution and an increased rocking‐curve FWHM, highlighting the sensitivity of crystallographic texture to the underlying substrate chemistry (Figure S16). Collectively, the pole figure and rocking‐curve analyses confirm the formation of a highly textured BiOI thin film with exceptional preferential (001) orientation and a low degree of orientational disorder.

SEM imaging shows the morphology of the 001‐BiOI films to be homogeneous in coverage, free from pinholes, and largely featureless, with flat areas spanning several tens of micrometers (Figure 2f). Higher magnification shows the film consists of overlapping nanoflakes (approximately 0.5–1 µm across) lying parallel to the substrate (Figure 2g). For comparison, an SEM image of a control (untreated) film fabricated in the absence of HI is shown, which displays a combination of flat and more vertically aligned nanoflakes. AFM imaging reveals that the control sample is rough with a root‐mean‐square (RMS) surface roughness of 44 nm. In contrast, the oriented BiOI film is highly smooth with an RMS surface roughness of only 8 nm. Wafer‐scale vdW layered 2D materials are indispensable for enabling large‐scale practical applications [61, 62, 63]. To present the versatility of our technique, large area oriented BiOI thin films were fabricated. Figure 2h shows an image of 001–BiOI on a 4‐inch wafer. These wafers are highly uniform and reflective. XRD patterns taken from various positions on the film surface confirm crystallographic alignment across the entire wafer (P(001) = 4.99). AFM measurements performed at the wafer center and edge locations yielded RMS roughness values in the range of 9.6–11.4 nm, indicating a uniform surface morphology across the wafer with no statistically significant positional dependence (one‐way ANOVA, p = 0.49) (Figure S17). This represents a significant step forward for 2D vdW layered BiOI applications that require ready access to large‐area functional films.

To investigate the atomic and nanostructure of these films high‐resolution transmission electron microscopy (HRTEM) imaging was performed on a focused ion beam (FIB) cut lamella. Figure 3a shows the cross‐sectional TEM image of a moderately thick (80 nm) (001)‐oriented BiOI thin film. A protective capping layer of platinum was deposited on top of the film to aid FIB cutting. A layering effect is observed in these films, which display long crystalline strands running parallel to the substrate as indicated by the labels (1–5). The distinct lines of separation between strands do not correlate to the number of layers deposited but rather arise from the layering of individually oriented nanoflakes. Figure 3b shows the selected area electron diffraction (SAED) pattern within a region spanning the entire film thickness (white circle), which shows periodically spaced bright spots corresponding to the {00*l*} plane reflections (yellow circles). The additional diffraction spots observed belong to the (004) and (022) reflection of the silicon substrate (blue circle) and thin native oxide (red circle), respectively. From these data a crystallographic *c*‐lattice constant of 0.913 ± 0.011 nm was determined (Figure S18), which corresponds well to the calculated *c*‐lattice constant for BiOI (0.915 nm) [45]. Figure 3c shows a cross‐section of the 001‐BiOI thin film at high magnification. The 2D crystal structure is clearly visible running parallel to the substrate. The on‐axis magnified region (Figure 3d) highlights the vdW stacking of covalently bonded 2D BiOI monolayers. A matched atomic structure diagram of BiOI is included to visually aid in identifying each vdW monolayer.

**FIGURE 3:**
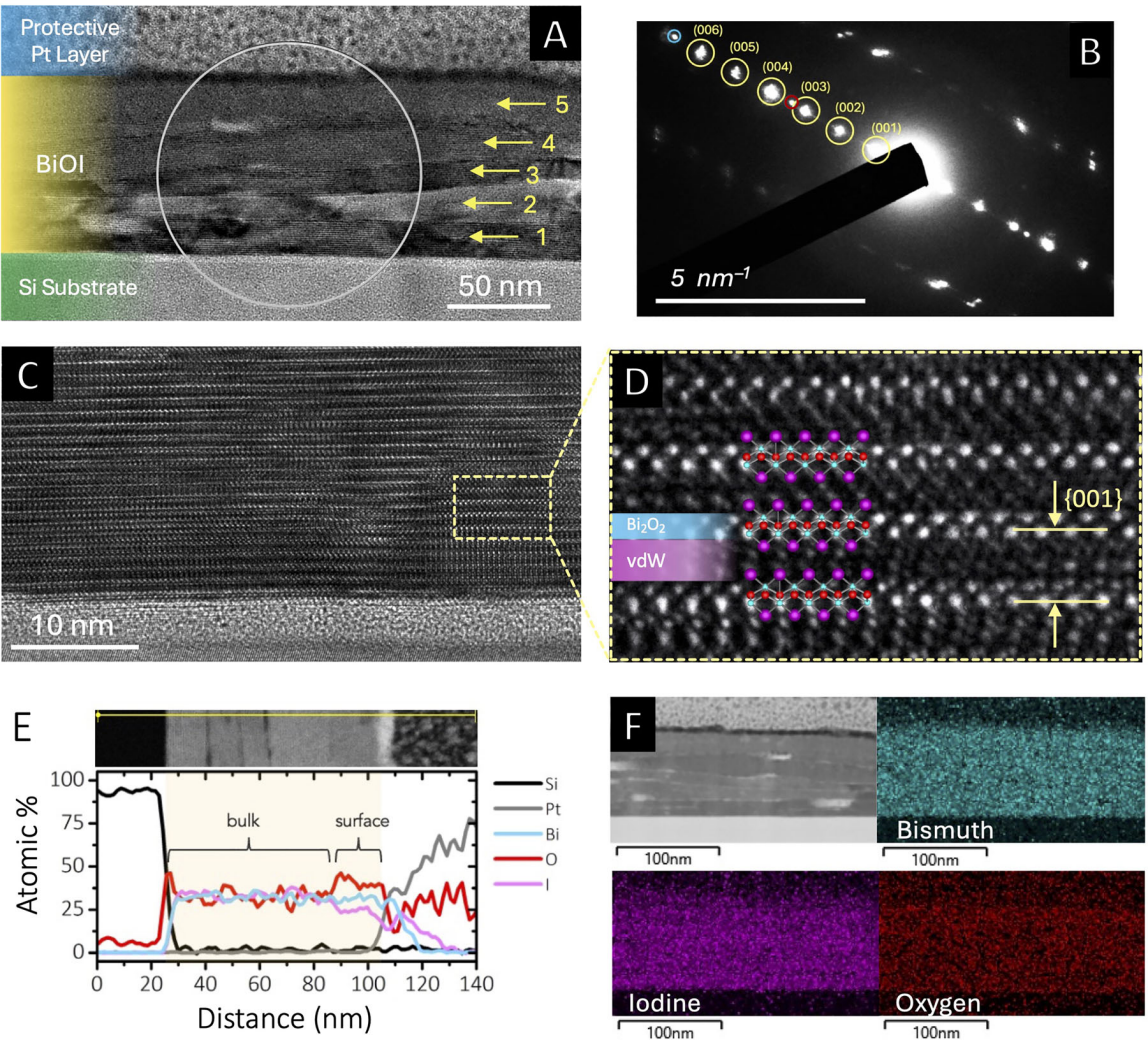
(a) Cross‐section FIB‐cut TEM image of 001‐BiOI, showing individually stacked layers of BiOI. The white circle defines the SAED region. (b) SAED pattern of 001‐BiOI, with indexed BiOI (yellow circles), Si (004) substrate (blue circle), SiO_2_ (022) native oxide (red circle) diffraction spots. (c) High‐resolution cross‐section TEM image of a 001‐BiOI thin film. (d) Magnified on‐axis region highlighting the vdW stacking. (e) Elemental quantification profile of the 001‐BiOI cross‐section. (f) Elemental maps of 001‐BiOI in cross‐section.

Multiple large‐area EDS spectra were also acquired, which yielded average percentages of (32.3 ± 1.8):(34.7 ± 2.6):(32.9 ± 1.7) for Bi:O:I (Figure S19). All elements are stoichiometric within error, but a slightly higher than average oxygen content is observed (∼1.5%). Line profile EDS demonstrates uniform stoichiometric atomic percentages of Bi, O, and I across the “bulk” of the film (Figure 3e). Lower iodine and elevated oxygen are noted near the film “surface” and is likely a consequence of FIB processing (hot deposition of the protective Pt layer) and beam exposure inducing volatile iodine loss [64, 65]. Broader‐scale elemental mapping (Figure 3f) confirms the uniform distribution of all key elements throughout the film.

Having established that optimal levels of HI effectively promote the near‐perfect (001) orientation, we now investigate the underlying cause of crystallographic alignment. The driving force for orientation may be a consequence of either the solution pH ([H^+^]) or the presence of free iodide ions (anions). The prevalent literature shows consistent evidence of increased (001) facet production and a subsequently elevated (001) texture for BiOX systems in acidic environments [66, 67, 68, 69]. However, all such studies were performed on bulk materials, which were shown to have pH *dependent morphologies*, thus convoluting the interpretation of texture coefficients (XRD peak ratios) as a function of pH. This has undoubtedly led to the spurious hypothesis that solution pH ([H^+^]) is the component influencing orientation in BiOX systems. Given the absence of controlled studies that appropriately deconvolute morphology and bulk crystal facet exposure from crystallographic texture, the mechanism of BiOX orientation in solution remains unknown.

To carefully decouple pH and anion effects, a series of detailed experiments were performed with hydrohalic acids, other common acids, and iodine‐based salts (neutral pH). We observed that both HBr and HCl had similar orienting effects, yielding high (001) texture coefficients. Notably, these texture coefficients decreased slightly in the order I > Br > Cl, indicating that while halides may orient BiOI, this is less effective for the smaller halides (Figure S20). EDS results from films oriented using HBr and HCl showed the presence of Br and Cl in significant enough amounts to conclude they must reside within the van der Waals layers of the final bulk crystal and are not simply surface adsorbates (Figure S21). As such, the hydrohalic acids liberate halide anions that participate in the vdW stacking of BiOI. The observation of sequentially broader (001) XRD peaks for HI, HBr, and HCl corroborates poorer vdW stacking for smaller halide anions (Figure S22).

Conversely, acids with larger counter cations (such as nitric, sulfuric, and acetic) are typically too bulky to reside within the vdW plane, and thus their activity may be more accurately measured as a function of acidity. Investigations with non‐hydrohalic acids revealed that they induce the growth of larger nanoflakes, consistent with previous reports [68, 69, 70, 71]. These acids also produce BiOI samples with elevated (001) textures, albeit to a slightly lesser extent than when hydrohalic acids were used (Figure S23). The observation of elevated (001) textures at low pH in BiOI may simply be attributed to the acid‐induced growth of larger (001) surfaces as well as their higher probability to lie slightly flatter on a substrate, both of which (artificially) enhance P(001), but are not actually associated with a pH‐based orientation mechanism. This was confirmed by our SEM analysis, which revealed that (despite elevation in the (001) texture coefficient) non‐halogen‐based acids did not result in the formation of highly ordered “flatter” (001) thin films (Figure S24).

To exemplify this, Figure 4a shows the SEM images for BiOI prepared using contrasting additives at neutral and acidic pH. The control BiOI thin film prepared using HI (pH ≈ 2) is seen to adopt its characteristic oriented (flat) morphology. However, films converted in nitric acid (pH ≈ 2), acetic acid (pH ≈ 2), and sulfuric acid (pH ≈ 2) are all seen to adopt a similar morphology to BiOI films converted in Milli‐Q water (pH ≈ 7), consisting of randomly oriented nanoflakes. In sharp contrast, BiOI thin films prepared in neutral iodine salts (sodium iodide (pH ≈ 7), ammonium iodide (pH ≈ 6), and guanidinium iodide (pH = 7)) displayed flat and highly (001)‐oriented films analogous to those formed using HI. These data confirm that the halide anion (not pH or counter ions) is the active component that drives high level orientation and morphological changes in BiOI. Texture analysis of samples prepared using a broad array of other iodine‐based salts (at neutral pH) further verified this phenomenon (Figure S25). Figure 4b shows how various iodide salt additives can be used to generate highly oriented (001) thin films with texture coefficients near unity (P(001) > 4.95), which are equal to or better than HI converted films. We note here that higher iodide concentrations (100 mm) are required compared to HI to generate an equivalent reservoir of available iodide given the lower dissociation constant of typical iodide salts compared to strong acids.

**FIGURE 4:**
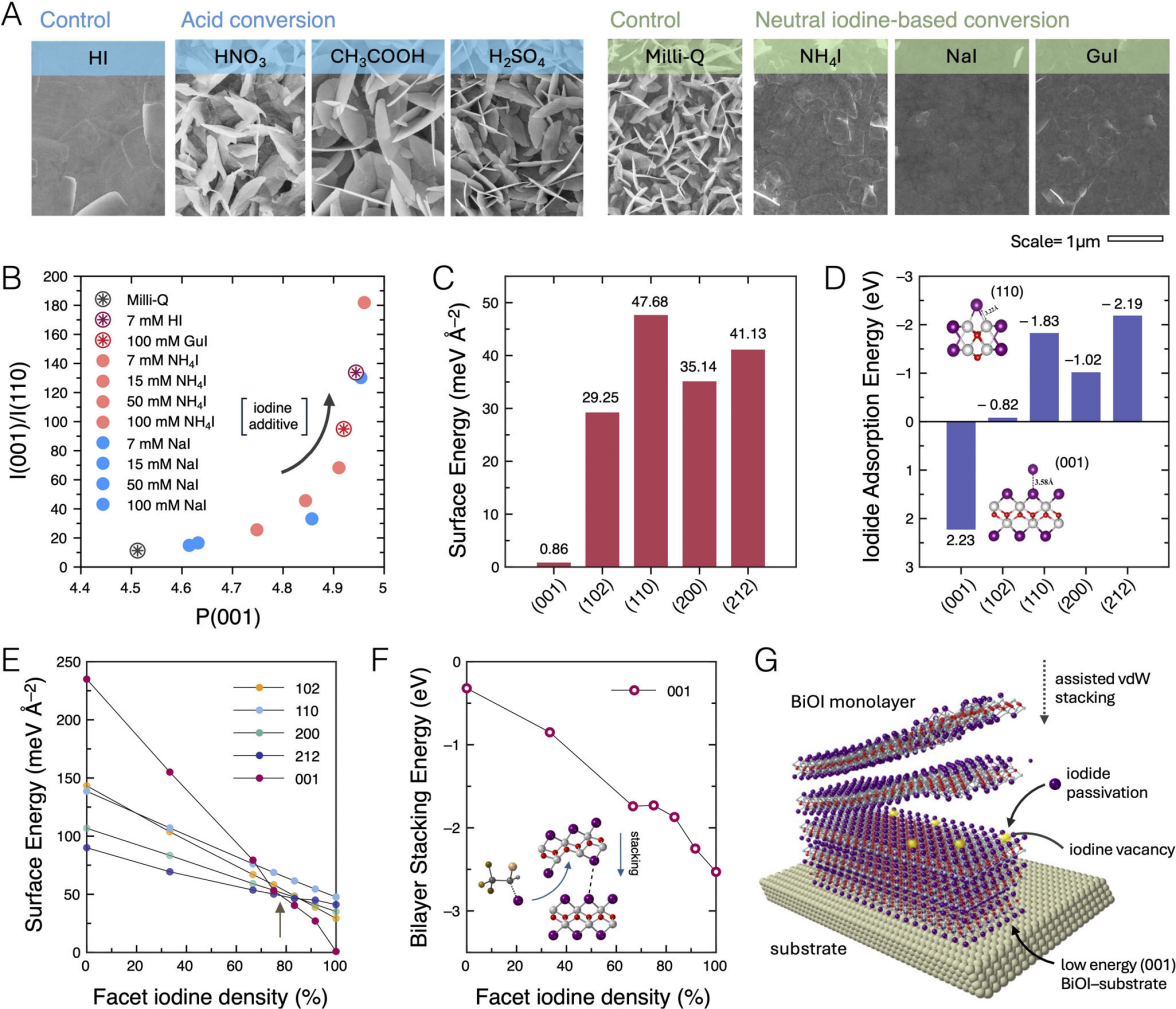
(a) SEM images of 3L (100 mg/mL) BiOI formed at acidic pH (∼2) with various acids and 5L (30 mg/mL) at neutral pH (∼6‐7) with iodine salt additives. (b) Plots I(001)/I(110) XRD peak intensity ratio as a function of P(001) texture coefficient for (5L 30 mg/mL) BiOI in various iodide salt additives. (c) Surface energy of different BiOI planes; (d) Adsorption energies of iodide (I^–^) onto BiOI planes; (e) Surface energy of BiOI planes as a function of exposed terminal iodine concentration; (f) bilayer (vdW) stacking energy of the BiOI (001) plane as a function of exposed terminal iodine concentration; (g) Schematic of halide‐assisted vdW stacking and (001) orientation of BiOI monolayers.

To further substantiate and explain the role of iodine in the growth and orientation of BiOI we performed density functional theory (DFT) calculations. The five key planes for BiOI were computed to investigate their formation and stability (Figure S26). The cohesive energies of all five planes were observed to be negative, thus inferring that their formation is feasible and the monolayers of BiOI in their respective orientations are achievable (Figure S27). Surface energy calculations revealed the (001) facet has a significantly higher stability compared to all other facets, with a remarkably low surface energy of only 0.86 meVÅ^−2^ (Figure 4c). This is consistent with the typical crystal growth habit of BiOI observed experimentally, which forms as flakes extending in the *a/b*‐axis direction (either as single nanoflakes, nanoflake arrays, or hierarchical “microflower” structures) exposing large (001) surfaces [22, 72, 73].

Evaluating the adsorption energy of I^–^ on different crystal facets was undertaken to learn about the growth behavior of BiOI. The adsorption of I^–^ on the (102), (110), (200), and (212) planes of BiOI involves chemical bonding between the exposed Bi atoms and I^–^ ions, which is not possible on the (001) plane (Figure S28). Calculations of the adsorption energy uncover that the adsorption of additional iodine atoms at the native (001) surface (i.e., generating I–I interactions) is not thermodynamically favorable (Figure 4d). This indicates that the growth of BiOI in the [001] direction (to generate vdW stacked layers) does not proceed by initial iodine adsorption and sequential atomic addition to form the new layer. Instead, growth must proceed via the formation of a (partial) BiOI layer followed by vdW stacking.

To accurately simulate the growth of BiOI in environments of varying [I^–^], the stability of BiOI monolayers was calculated as a function of surface iodine concentration. Figure 4e shows the surface energies of the five predominant BiOI planes as a function of the iodine concentration on their terminal face. We observe that the density of terminal iodine atoms plays a crucial role in the stabilization of BiOI planes and thus can influence growth dynamics. While all planes are sensitive to changes in iodine content, the (001) facet is the most energetically susceptible. Importantly, it is only when the facet iodine density on the (001) surface is very high (above ∼80%) that its surface energy drops below that of all other planes. XPS experiments of 001‐BiOI showed small but significant increases in surface iodine (∼4%) compared to control samples converted in a pure water bath (consistent with previous bulk EDS analyses) (Figure S29). Iodine vacancies (V_I_) are known to have the lowest formation energy (donor defect) [19]. High (optimal) iodine concentrations in solution are needed to provide maximum coverage (minimize V_I_) and subsequently minimize the (001) surface energy. The preferential growth of a (001) film necessitates the facilitated vdW stacking of BiOI monolayers. To evaluate this phenomenon, the growth of a BiOI monolayer on an existing (001) facet was investigated via calculations of the bilayer stacking energies (Figure 4f). These calculations show that the vdW stacking of BiOI bilayers is far more efficient as the concentration of terminal iodine increases.

Figure 4g shows a schematic summarizing the mechanism of halide‐assisted vdW stacking and orientation in BiOI. The addition of iodide (halide) anions in our experiments leads to the passivation of iodine vacancies and the selective thermodynamic stabilization of the (001) facet, which provides favorable substrate interactions and reduces the vdW (bilayer) stacking energy, culminating in highly (001)‐oriented thin films. The implications of this finding are groundbreaking. Having identified the root cause of orientation, adjusting the availability of free iodine/halide during synthetic reactions (by either supplemental addition or reactive removal) grants direct control over the key crystal facet energies to manipulate orientation in BiOX systems. In fact, we believe that this should extend to vacuum systems as well, through the control over the vapor phase concentration of iodine gas (Reaction 2). Furthermore, the BiOI system is known to undergo facile topotactic conversions to other compounds. This has already been proven for BiOI/oxyhalide heterostructures (BiOBr, BiOCl) [74, 75, 76], as well as Bi_2_O_2_(CO_3_) [77, 78] and Bi (metal) [16, 52, 79]. As such, we expect added downstream advantages through the fabrication of films with controlled orientation and facet exposure across a wide variety of analogous material systems for various applications in optoelectronics and photocatalysis using BiOI templates.

Following the development of an orientation‐controlled fabrication method, the effects of BiOI morphology and orientation on the optoelectronic properties of thin films were investigated. BiOI films were integrated into lithographically patterned lateral photodetectors (PDs) consisting of Cr/Au electrodes on Si/SiO_2_ substrates (see Experimental). Figure 5a presents the measured current‐voltage (*I–V*) curves in the dark and at various illumination powers using a λ= 455 nm LED. The 001‐BiOI PD exhibits distinct linear behavior and Ohmic contact at the electrode junction (Figure S30). In contrast, the randomly oriented (r‐BiOI) device showed minor Schottky barrier characteristics. Immersion of the r‐BiOI device in dilute 1 mm HI for 30 min showed that the Schottky barrier characteristics persisted, verifying that the junction behavior is predominantly influenced by the BiOI morphology and not a result of the higher [I^–^] content in 001‐BiOI films (Figure S31).

**FIGURE 5:**
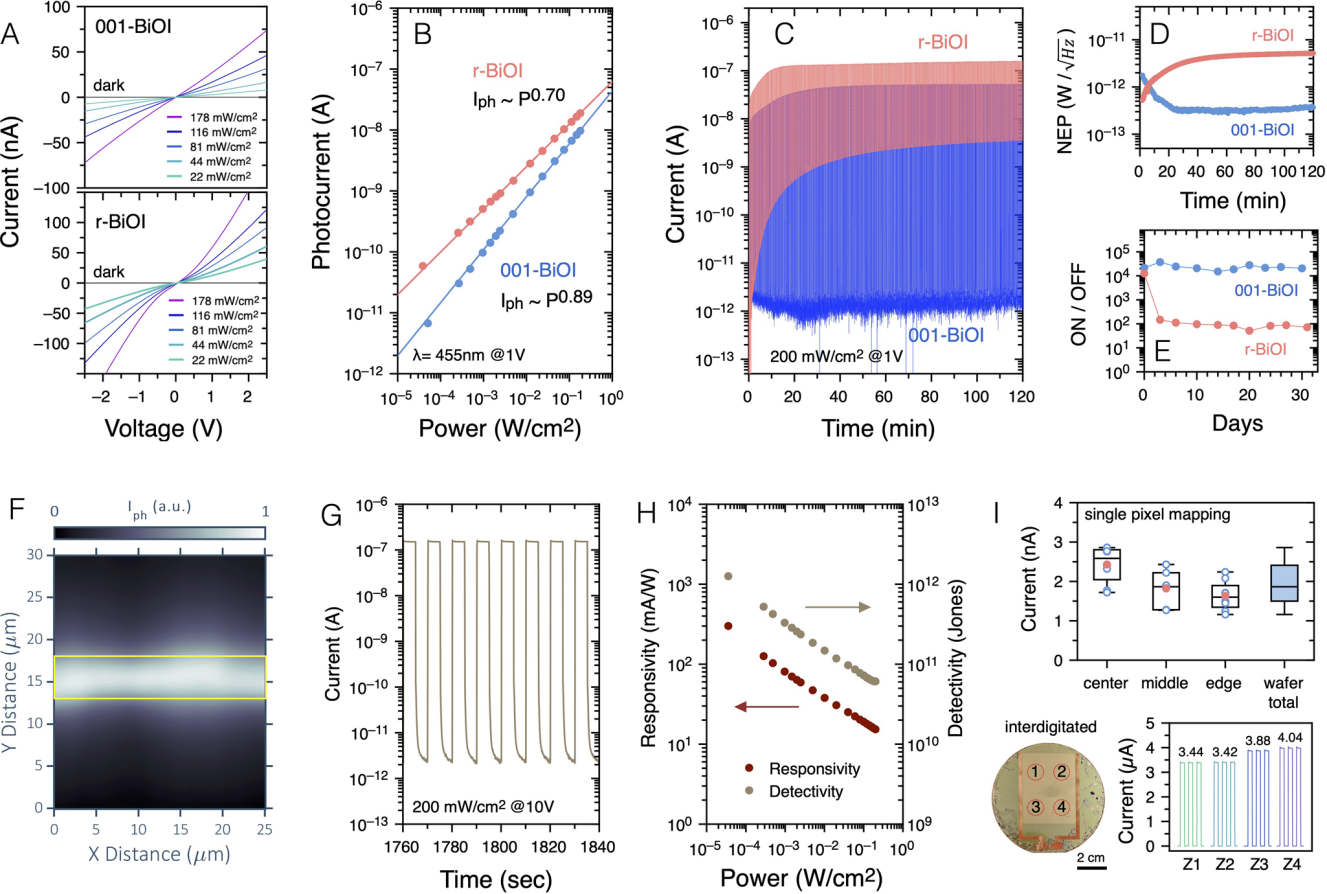
(a) Typical (*I‐V*) curves for 001‐BiOI and r‐BiOI (2L, 150 mg/mL). (b) Photocurrent as a function of illumination power and extracted power‐law trends for 001‐BiOI and r‐BiOI. (c) *I‐t* pulsed (5 s ON/OFF) responses showing the differences in operational stability of 001‐BiOI and r‐BiOI. (d) *NEP* over time for 001‐BiOI and r‐BiOI. (e) Long‐time operational ON/OFF of 001‐BiOI and r‐BiOI. (f) Photocurrent map of a section of device channel for a typical 001‐BiOI PD. (g) Pulsed (5 s ON/OFF) light response for a champion device. (h) Evaluated *R* and D* of champion 001‐BiOI PD. (i) Single pixel mapping at key positions across a 4” wafer (top), interdigitated electrode array on a 3” wafer and the corresponding pulsed photo response in each quadrant (bottom).

The photocurrent (*I_ph_* = *I_light_* − *I_dark_*) is a key parameter in the optoelectronic performance of a material and standard PD characterization, which may be described by a power‐law relationship, *I_ph_* =  *AP*_λ_^α^, where *A* is a constant, *P*_λ_ is the irradiance of the incident light at a specific wavelength, and α is an empirically fitted exponent. In well‐behaved semiconductors a linear relationship between *I_ph_* and irradiance is observed (*α* = 1), indicating few alternate pathways to direct charge carrier collection at the electrodes, that is, a largely “trap‐free” response. Sublinear behavior (*α* < 1) is associated with a higher density of trap states [80, 81]. Figure 5b illustrates the extracted power‐law trends for r‐BiOI and 001‐BiOI PDs over four orders of magnitude in irradiance (measured after a short light soaking period). The r‐BiOI PD exhibited a sub‐unity power‐law exponent (*α* = 0.70), while the 001‐BiOI PD displays a far higher exponent (*α* = 0.89), indicating a substantially reduced native trap‐state density in oriented films. The high exponent observed for 001‐BiOI is on par with or better than BiOI made from high temperature CVD routes [32, 82]. Notably, pristine 001‐BiOI displays an exceptional near‐unity exponent (*α* = 0.99) at low power irradiances, which changes after the first measurement (Figure S32). Such changes in the photocurrent dynamics and power law exponents (under light and bias) are indicative of a change in the trap state depth and density in the films, consistent with mobile atoms/ions.

To further investigate these changes, the operational photoresponse of r‐BiOI and 001‐BiOI PDs was investigated. Figure 5c displays the measured current‐time (*I–t*) response of both PDs over a 120‐min period under pulsed 455 nm illumination cycles. Both types of PDs exhibited a light‐soaking effect at early times, evidenced by the gradual increase in *I_light_* prior to reaching a steady‐state. However, the *I*_*dark* _ of the r‐BiOI PD is observed to continuously rise over 3‐orders of magnitude during the early stages of operation, while the 001‐BiOI displayed no significant changes in *I*_*dark* _ and superb operational stability.

Based on these findings, further assessment of our devices was conducted using key figures of merit, including responsivity (*R*), total noise‐equivalent power (*NEP*), and specific detectivity (D*):

(3) $$R=\frac{I_{ph}}{S\times P_{\lambda }}\left(A/W\right)$$

(4) $$NEP=\frac{i_{noise}}{R}\left(W\right)$$

(5) $$D^{*}=\frac{\sqrt{S\times \Delta f}}{\mathrm{NEP}}\left(\mathrm{cm}\cdot {\mathrm{Hz}}^{1/2}\cdot W^{-1}\;\mathrm{or}\;\mathrm{Jones}\right)$$

where *S* is the active area, *i_noise_* is the noise current and ∆*f* is the individually determined measurement bandwidth (average ∆*f* ≈ 12.5 Hz). These parameters enable the quantitative analysis of a device to convert optical signals into electrical output, alongside estimating overall sensitivity to low‐intensity light. Figure 5d presents the estimated *NEP* derived from the previously discussed *I–t* measurements. As our PDs operate under the Gain threshold (Figure S33) in this analysis, *i_noise_* may be reliably approximated by the sum of the shot noise ($\sqrt{2qI_{dark}\Delta_{f}}$) and thermal (Johnson) noise ($\sqrt{(4k_{B}T\Delta_{f}/R_{dark\;}})\;$ currents (white noise), where *q* is the elementary charge, *k_B_* is the Boltzmann constant, *T* is the temperature, and *R_dark_* is the dark resistance. Given that *i_noise_* is predominantly influenced by *I_dark_*, r‐BiOI experiences a substantial degradation in performance. This increases the device noise (*NEP*), resulting in a notable decline in device sensitivity over time (1 order of magnitude). In contrast, the 001‐BiOI PD maintains a stable noise floor (*NEP* = ∼3 × 10^−13^ W/$\sqrt{Hz}$) approximately 16 times lower than r‐BiOI, preserving detectivity throughout device operation (Figure S34). Periodic measurements confirmed that our 001‐BiOI devices remained stable for 1 month under high power use (180 mW/cm^2^), retaining an ON/OFF switching ratio of > 10^4^ (Figure 5e; Figure S35). In contrast the r‐BiOI device, which had good initial switching, degraded to show poor ongoing performance due to the large increase in *I_dark_*.

Increases in dark current for BiOI have been previously reported and attributed to the formation of oxygen vacancies [32]. Dark current drift has also been associated with halide migration in single‐crystal BiOI [43]. Likewise, iodine (halide) migration is known to impart signal hysteresis and degrade dark current in lead halide perovskites [83]. The light‐induced operational degradation observed in r‐BiOI is therefore a consequence of a far more dynamic trap landscape compared to 001‐BiOI. The elevated iodine content observed in 001‐BiOI is expected to partially passivate iodine‐vacancy (*V_I_*) defects.

To investigate whether orientation and iodine passivation modify the density and distribution of electronically active defect (trap) states in BiOI, space‐charge‐limited current (SCLC) measurements were performed on electron‐only devices. Initial experiments were conducted on control and HI‐treated r‐BiOI films to quantify trap density, trap‐filling behavior, and carrier mobility. HI treatment (by film immersion in 1 mM HI for 2 hrs) resulted in a substantial reduction in trap density, with analyses indicating decreases of approximately 33% and 40% for the thin (∼350 nm) and thick (∼550 nm) films, respectively, accompanied by corresponding improvements in carrier mobility (Figure S36). To assess the effect of crystallographic orientation, thin HI‐treated r‐BiOI films were compared with highly oriented 001‐BiOI films. The (001)‐oriented films displayed a much sharper trap‐filling transition, more well‐defined SCLC behavior, and an effective device mobility over three orders of magnitude lower than the HI‐treated r‐BiOI films (Figure S37). This indicates that crystallographic orientation independently exerts a profound influence on charge transport. The poor vertical transport observed in 001‐BiOI is consistent with intrinsic transport anisotropy associated with carrier motion across the layered vdW structure. Accordingly, charge transport parallel to the BiOI layers is expected to be comparatively more favorable in lateral device architectures. Overall, iodine incorporation improves the electronic quality of BiOI through trap passivation, with 001‐BiOI exhibiting remarkable resistance to light‐induced defect activation and dark‐current drift, which degrade performance in poorly formed unoriented (r‐BiOI) devices. These results further highlight the importance of crystallographic control in achieving electronically stable BiOI thin films.

To better define our 001‐BiOI PDs, a spatial photocurrent current map of a typical 001‐BiOI PD was measured (Figure 5f). The corresponding spatial current map reveals that *I_ph_* is largely confined to the active channel region (5 µm), confirming the localization of photogenerated carriers. Slight signal inhomogeneity within the physical channel region of the map (yellow box) is observable. As such, calculated figures of merit will be slightly underestimated by assuming a physically defined active area (1 × 10^−4^ cm^2^), indicating that additional improvements to device design may further improve device performance.

Device optimizations were conducted by varying the precursor concentration (template thickness) and layer number to maximize device performance. Optimal performance was consistently achieved from our most (001)‐oriented samples, that is, a thin template, high‐layer number approach, with the best‐performing device fabricated with eight layers (8L) of a 30 mg/mL precursor solution (Figure S38). Figure 5g displays the segmented *I‐t* response of the champion device under an applied potential of 10 V. The device demonstrated excellent stability over a 30‐min period (Figure S39) and exhibits an ON/OFF switching ratio approaching ∼10^5^. Furthermore, temporal analysis found the device to exhibit sub‐millisecond responsive behavior, with rise and fall times estimated to be ∼488 and ∼645 µs, respectively, although signal response times of ∼40–50 ms are more common for moderate light intensities (Figure S40). Figure 5h shows overall 001‐BiOI device performance (*R* and D* values). Under low‐light conditions (35 µW/cm^2^), our champion device achieved a responsivity of 300.4 mA/W and a specific detectivity of 1.25 × 10^12^ Jones. These figures of merit rival those of the best previously reported bismuth oxyhalide photodetectors while offering a scalable low‐temperature fabrication platform that avoids the practical limitation of single‐nanosheet devices (see Table S1).

To provide a preliminary assessment of wafer‐scale electronic uniformity, photocurrent mapping was performed across 22 individual device locations (50 µm channel width, 1 mm channel length, 1 V bias, 180 mW/cm^2^). As shown in Figure 5i, the devices exhibited an average photocurrent of 1.98 ± 0.55 nA, with a gradual decrease in photocurrent from the wafer center to the edge (∼30% reduction; pink data points). Despite these spatial variations, device performance remained reasonably consistent across the wafer while exhibiting excellent temporal stability (Figure S41). Additionally, photocurrent mapping of a 3‐inch interdigitated electrode array demonstrated improved large‐area uniformity, yielding an average photocurrent of 3.70 ± 0.31 µA. The photocurrent varied by only approximately ±8% across the four measured quadrants of the wafer (Z1‐Z4) (Figure S42). The observed level of uniformity is particularly encouraging for early‐stage development, indicating that the favorable electronic properties of the BiOI films are largely maintained across wafer‐scale substrates.

## 3 Conclusion

We have demonstrated a novel, facile, room‐temperature solution‐based procedure to achieve highly (001)‐oriented BiOI thin films with near perfect texture on a wafer scale. Such precise (001) orientation has not been demonstrated previously. Through a series of in‐depth experiments and molecular modeling we have provided conclusive evidence that the mechanism of orientation in BiOI and related oxyhalide systems is directly correlated to the extent of halide passivation at the (001) facet during the growth process. This work redefines the previous hypothesis that orientation in bismuth oxyhalide systems is based on the pH of the growth medium. The insights provided in this work now enable researchers to manipulate crystal orientation in BiOX systems through a strategic approach, focusing on either the addition or removal of halide from the reaction system. We expect this will also prove valuable to control orientation in the vacuum‐based deposition of thin films by controlling the vapor phase concentrations of halide gas. Our results show that orientation and improved iodide passivation strongly influence the optoelectronic performance and stability of BiOI thin‐films, as evidenced by the high detectivity, low NEP, and long operational lifetime of our 001‐BiOI photodetectors. Bismuth chalcohalides is a fast‐emerging field of research wherein crystal orientation and controlled facet exposure are critical to advance their many applications. Our work introduces a new framework that fundamentally changes how orientation in bismuth chalcohalide systems is approached.

## 4 Experimental

### 4.1 Materials

Bismuth iodide (99%), hydroiodic acid (57 wt. % in H_2_O, 99.99%), hydrobromic acid (48 wt.% in H_2_O), hydrochloric acid (32 wt. % in H_2_O), sodium iodide (≥99.5), ammonium iodide (≥99.0), nickel(II) acetate tetrahydrate (>99%), ethanolamine (>99.0%), titanium(IV) isopropoxide (99.999%) were purchased from Sigma‐Aldrich. Tetrahydrofuran (99.9%) was purchased from RCI Labscan and dried to ∼30 ppm water via two packed columns of neutral alumina under argon and filtered through a 2 µm Nupro TF series Tee‐type sintered filter. Acetone (≥99.5%), 2‐propanol (≥99.5%), nitric acid (70% in H_2_O), sulfuric acid (96%), and acetic acid (glacial) were purchased from Ajax Finechem. Guanidium iodide was purchased from Great Cell Solar Materials.

### 4.2 Substrate Cleaning

Prior to use, all substrates (Pilkington) were cleaned via sonication sequentially in detergent‐water, acetone, and 2‐propanol solvents for 10 min each. Cleaned substrates were then dried with a nitrogen stream. Dried substrates were then UV/ozone cleaned for 15 min prior to spin coating to remove any remaining organics from the substrate surface.

### 4.3 BiOI Thin Film Fabrication

A BiI_3_ ink solution was prepared by completely dissolving BiI_3_ powder in dried THF. Concentrations typically varied from 30 mg/mL to 300 mg/mL. The solution was filtered with a 0.22 PTFE filter before being spin coated (in air) onto the target substrate at 4000 rpm (2000 rpm/s acceleration) for 30 s. The BiI_3_ thin films were then annealed at 150°C for 2 min in air and then cooled to room‐temperature. The BiI_3_ thin films were then immersed in a beaker containing 1 L of Milli‐Q water (unless otherwise specified, i.e., 7 mM HI) for 5 min to ensure the complete hydrolysis of BiI_3_ into BiOI. The precise bath volume and substrate size are not critical parameters. Multiple substrates may be converted in the same bath, provided the water volume is sufficient to prevent significant HI accumulation during hydrolysis. These thin films were then rinsed in fresh Milli‐Q water and then isopropanol before being dried with a nitrogen stream and further annealed in air at 150°C for 2 min. This process is then repeated (in a layer‐by‐layer fashion) to achieve BiOI thin films of desired thicknesses, with the final layer annealed at 150°C for 5 min. For the large area 4″ wafer conversion, a 2L bath was used.

### 4.4 Photodetector Electrode and Device Fabrication

Metal contacts were fabricated on a highly conductive *p*‐doped silicon substrate with 300 nm thermally oxidized SiO_2_ (D&X Co. Ltd.) using standard photolithography processes. Source/drain electrodes were patterned with a maskless aligner (MLA150‐Heidelberg Instruments), followed by electron‐beam deposition of 100 nm Au metal contacts at a base pressure of < 5 × 10^−7^ Torr, with 10 nm Cr as an adhesion layer (PVD75‐Kurt J. Lesker). The resulting patterned contacts were then realized through a lift‐off process in an acetone bath. BiOI thin films were then prepared on the pre‐patterned substrates, with layer‐by‐layer conversions repeated until a desired film thickness was achieved.

Electron‐only devices were fabricated on patterned FTO substrates (Latech Scientific Supply (15 Ω/sq)), TiO_2_ was deposited as outlined in the Supporting Information. BiOI was fabricated with different thicknesses and crystallographic orientations. The devices were finished with ZnO nanoparticle—aluminum top contact (ZnO/Al) as outlined previously [42].

### 4.5 Computational Modeling

Density functional theory (DFT) calculations were carried out using a projected augmented wave (PAW) method implemented in the Vienna Ab initio Simulation Package (VASP 5.4) [84]. A conjugate gradient method with PBE functional and plane wave basis set with an energy cutoff of 300 eV was sampled at a (2 × 2 × 1) *k*‐point grid using the Monkhorst−Pack scheme to describe the ionic interactions of BiOI films [85]. Spin‐polarized DFT calculations were carried out with long‐range DFT‐D3 dispersion correction with a vacuum space of 30 Å along the Z‐direction to avoid the interactions between the different nearest neighboring BiOI layers [86]. Further details are included in Supporting Information.

## Conflicts of Interest

The authors declare no conflicts of interest.

## Supporting information




**Supporting File**: adma74729‐sup‐0001‐SuppMat.pdf.

## Data Availability

The data that support the findings of this study are available from the corresponding author upon reasonable request.
