# Supplementary material for: Directing Near‐Perfect (001) Orientation in Wafer‐Scale BiOI Thin Films via Halide‐Mediated van der Waals Stacking
Source: Adv Mater. 2026 Sep 23;38(62):e74729. doi: 10.1002/adma.74729 (PMC13741101; doi:10.1002/adma.74729)
Supplement: Supplementary file 1 [file ADMA-38-e74729-s001.pdf]

Supporting Information

Directing Near-Perfect (001) Orientation in Wafer-Scale BiOI Thin Films via Halide-Mediated van der Waals Stacking

Authors: *Gabriel Aygur*, Ashakiran Maibam*, Hayden Tuohey*, Tony Nguyen*, Mei Xian Low¶, Sumeet Walia¶, Zijun (June) Yong*, Ravichandar Babarao*, Daniel Gomez*, Enrico Della Gaspera*, Joel van Embden**

* School of Science, RMIT University, Melbourne, VIC 3000, Australia

¶ School of Engineering, RMIT University, Melbourne, VIC 3000, Australia

E-mail: joel.vanembden@rmit.edu.au

Substrate Layers: NiO_x & TiO_2

For BiOI textural and morphological analysis a NiO_x precursor solution was prepared by adapting a method from Chen et al.[1]. For our work we prepared a 0.2 M solution. NiO_x thin films were fabricated by spin coating at 3000 rpm (1500 rpm/s acceleration, 30 sec). After spinning, substrates were heated at 275°C for 5 minutes in air then allowed to cool to room temperature. After this initial layer, a second layer was spun and substrates were heated at 275°C for 1 hr in air, then allowed to cool to room temperature.

For BiOI UV-Visible characterization, TiO_2 precursor was synthesized and used following the method adapted from Zhang et al., (2016) which outlines the synthesis of a 0.23 M titanium (IV) isopropoxide and 0.013 M HCl solution in 2-propanol [2]. TiO_2 thin films were spin coated at 3000 rpm (1500 rpm s-1 acceleration) for 30 sec. After spinning, substrates were heated at 275°C for 2 minutes, then 500 °C for 5 minutes, and 275 °C for 2 minutes. The second layer was spun, and substrates were heated at 275°C for 2 minutes, then 500 °C for 1 hr. To prevent substrate cracking, a sequential cooling to 275°C and then 100°C was implemented.

UPS Characterisation

To obtain reliable spectral data from the UPS characterisations, the BiOI thin film samples were prepared and measured on Si/Au substrates. This was done to mitigate effects from charging, as much as possible. To conduct these characterisations, we thermally evaporated a secondary Au region on the surface of our sample, to allow for charge correction via a reference. As minimal difference between the charge offset of the bare substrate and second Au region was observed, we utilised the secondary region for referencing and charge correction of all samples.

Texture Analysis

The Texture Coefficient (P) can be described as the deviation of our diffraction intensity ratios compared to that of the reference BiOI-ICCD#10-0445, assisting in the indication of the preferred (hkl) plane for crystal growth. The texture coefficient is defined as follows

$$P_{(hkl)} = \frac{I_{(hkl)}}{I_{0(hkl)}} \left\{ \frac{1}{n} \sum \frac{I_{(hkl)}}{I_{0(hkl)}} \right\}^{-1} \qquad (1)$$

where P(*hkl*), I(*hkl*), I_0(*hkl*), and *n* represent the texture coefficient of the specified (*hkl*) plane, the measured intensity, the reference intensity, and the total number of included reflections, respectively. For BiOI, the reflections included for texture quantification consisted of the (001), (102), (110), (200), and (212) planes, as these represent the most significant peaks with respect to BiOI-ICDD# 10-0445, possessing intensities above 20% relative to the strongest diffraction peak. As a total of number of *n* reflections are incorporated, this signifies that any (*hkl*) plane with a texture coefficient value close to or equal to *n* would represent near-perfect texture for that set of {*hkl*} reflections.

Scherrer (Crystallite) And Strain Analysis

The Scherrer equation, which coincides with peak broadening and crystallite size, can be defined as

$$\tau = \frac{K\,\lambda}{\beta\ \cos\theta} \qquad (2)$$

where τ, *K*, λ, β, and θ represent the mean crystallite size, shape factor (0.89), the X-ray wavelength, the full width half maximum (FWHM), and the Bragg angle, respectively.

The estimated Strain of a diffraction peak can be defined as

$$\varepsilon = \frac{\beta}{4\tan\theta} \qquad (3)$$

Rocking Curve and Pole Figure Analysis

For Rocking curve data collection, the sample was carefully aligned for sample height, sample rotation (Omega) and sample tilt (Psi). In order to collect data in a suitable rocking curve range, the (004) plane was chosen with its fixed diffraction angle in coupled 2theta/omega mode. A HyPix-3000 Hybrid Pixel Array Detector running in 1D mode, with a step size of 0.5° and a scanning speed of 5° per minute. An 0.5 mm incident slit and a 0.5 mm receiving slit were used. Other optics include 2.5° Soller slits on both primary and secondary sides. For the (004) Pole Figure analysis, a CBO-f polycapillary optic was used for point beam conditioning. The HyPix-3000 Hybrid Pixel Array Detector runs in 2D mode. The data was collected and reduced with 5° intervals for both rotation angle and tilt angle with a tilt angle range up to 70°. The data was processed with SmartLab Studio II software and normalised for comparison purposes.

Photocurrent Spatial Map Characterisation

For the set-up, a 405 nm continuous wave laser was used as the illumination source. This was coupled with a 280 APC-A fibre collimator, 405 nm laser line filter, 405 nm dichroic mirror, 405 nm notch filter, 100x air objective, and a 25% Excelitas avalanche photodiode (APD). A FEMTO DLPCA-200 variable gain transimpedance amplifier was used to amplify the photocurrent signal, applying a 2 V bias, low noise performance range setting, transimpedance gain of 10^4 V/A, and 10 Hz low pass filter. Pixel time during spatial mapping was set to 10 ms.

Computational Modelling

The feasibility of formation of BiOI monolayers were computed through the cohesive energies and their relative stability were further calculated in terms of their surface energy. The following equations were used to calculate cohesive energies, E_{coh} and surface energies, γ of the BiOI monolayers

$$E_{coh} = \frac{1}{n}(E_{BiOI} - N_{Bi}E_{Bi} - N_O E_O - N_I E_I) \qquad (1)$$

where n is the total number of atoms in a BiOI monolayer, N_x and E_x are the numbers and electronic energies of constituent Bi, O, and I atoms.

$$\gamma = \frac{1}{2A}(E_{BiOI} - n \times E_{bulk}) \quad (2)$$

where A is the surface area of BiOI monolayer, E_{BiOI} is the total energy of the BiOI monolayer, E_{bulk} is the electronic energy of BiOI unit cell and n is the number of BiOI unit cells in BiOI monolayer (ML).

The feasibility of BiOI thin film growth over another layer has been evaluated in terms of its bilayer stacking energy (BLSE), which is calculated as

$$BLSE = E_{stacked\ bilayer} - E_{1st\ ML} - E_{2nd\ ML} \quad (3)$$

Instrumentation

X-ray diffraction (XRD) patterns were measured on a Bruker D4 Endeavour diffractometer, equipped with a Cu-Kα radiation source. The Rocking Curve and Pole Figure data were collected using a Rigaku SmartLab X-ray diffractometer with Cu Kα radiation (1.5406 Å) equipped with a rotating anode, which was operated at a generator voltage of 50 kV and a current of 200 mA. The X-ray beam was conditioned with a Cross Beam Optics (CBO) and a Parallel Beam (PB) selection slit. Scanning Electron Microscopy (SEM) imaging was conducted on a FEI Verios 460L SEM operated at an accelerating voltage of 3 kV and current of 13 pA. UV-Visible spectra were collected on a Cary 7000 UV–vis–NIR spectrophotometer, equipped with an integrated sphere attachment and center-mount sample holder. Ultraviolet photoelectron spectroscopy (UPS) analysis was conducted on a Kratos AXIS Supra XPS instrument, with He I (21.2 eV) low photon energy. Two-dimensional diffraction patterns were measured on a Bruker D8 Discover general area detector diffraction system (GADDS) instrument, equipped with a Cu-Kα radiation source. Transmission electron microscopy (TEM) imaging and selected area electron diffraction (SAED) was conducted on a JEOL JEM-F200 TEM instrument, while elemental analysis was performed via the included scanning TEM energy-dispersive x-ray spectroscopy (STEM-EDS) function. Surface roughness was characterized on an Asylum Research MFP-3D Infinity Atomic Force Microscope (AFM) instrument. Photodetector devices were measured with a Keithley 2636B source measure unit (SMU). For photodetection, incident light consisted of a blue (455 nm; M455L4 – ThorLabs) LED source, with pulse modulation generated by a DC2200 High-Power 1-Channel LED Driver (ThorLabs). Light intensities were measured and calibrated using a PM16-121 Standard Photodiode Sensor (ThorLabs). Spatial current mapping was conducted with the excitation source consisting of a 405 nm continuous wave laser (Thorlabs), alongside a DLPCA-200 variable gain transimpedance amplifier (FEMTO).

Table S1: Key figures of merit for BiOX-based photodetectors and other bismuth perovskite inspired materials. Definitions: TF, Thin Film; NS, Nanosheet; SC, Single Crystal, SOL, Solvothermal; CVD, Chemical Vapour Deposition; SP, Solution-Processed; MS, Molten Salt; *, estimated; -, not stated.

Absorber Material	Method	λ (nm)	*P* (W/cm^2)	Bias (V)	*R* (A/W)	*D** (Jones)	Ref
BiOCl-NS	SOL	320	9.10 x 10^{-4}	30	* 4 x 10^{-2}	* 1 x 10^{10}	[3]
BiOBr-NS	CVD	340	1.5 x 10^{-5}	-10	14.96	5.74 × 10^{10}	[4]
BiOBr-NS	CVD	365	4.6 x 10^{-4}	8	12.4	1.6 × 10^{13}	[5]
BiOI-NS	SP	454<br>535	1 x 10^{-4}<br>1 x 10^{-4}	2<br>2	5.91 x 10^{-2}<br>6.70 x 10^{-2}	*3.28 x 10^{10}<br>*3.27 x 10^{10}	[6]
BiOI-TF	CVD	405	1.16 x 10^{-5}	-10	4.35 x 10^{-2}	8.70 x 10^{10}	[7]
BiOI-NS	CVD	473	2.83 x 10^{-4}	10	2.60 x 10^{-2}	8.20 x10^{11}	[8]
BiOI-NS	MS	532<br>310	5 x 10^{-3}<br>9 x 10^{-5}	-5<br>-10	0.014<br>1.05	6.46 x 10^{11}<br>4.70 x 10^{12}	[9]
$MA_3Bi_2I_9$-TF	SP	~620	1 x 10^{-5}	0.8	1.6 x 10^{-2}	4.6 x 10^{12}	[10]
$MA_3Bi_2I_9$-TF	CVD	382	4 x 10^{-5}	0	0.28	8.8 x 10^{12}	[11]
$Cs_3Bi_2I_9$-SC	SP	405	2 x 10^{-5}	5	0.1	2.55 x10^{13}	[12]
$Cs_3Bi_2I_9$-SC	SP	white	1 x 10^{-6}	-2	7.2 x 10^{-2}	1 x 10^{11}	[13]
$Cs_3Bi_2I_9$-SC	SP	white	0.1	-4	0.16	-	[14]
$Cs_2AgBiBr_6$-TF	SP	265	5 x 10^{-5}	0	1.46	9.3 x 10^{12}	[15]
$Cs_2AgBiBr_6$-TF	SP	445	-	0	0.14	3.3 x 10^{12}	[16]
BiOI-TF	SP	455	3.56 x 10^{-5}	10	0.3	1.25 x 10^{12}	This work

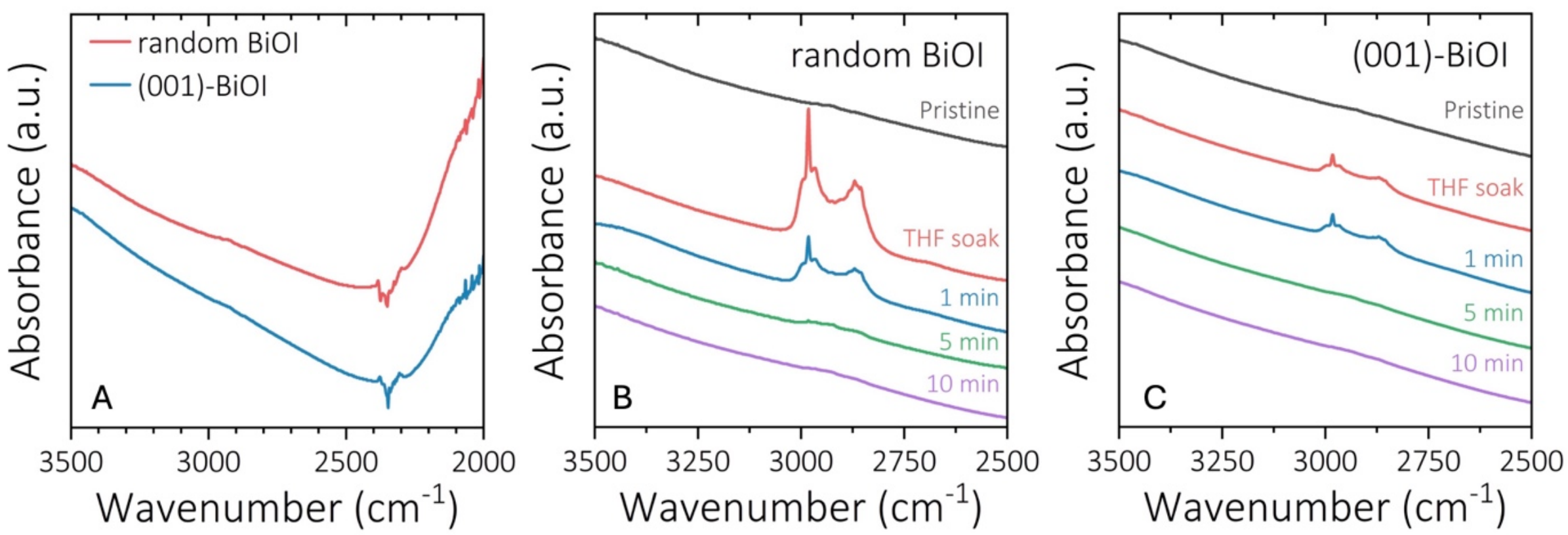

Figure S1: a) FTIR characterisation of typical random and (001) oriented BiOI thin films. The pristine (as-prepared) BiOI thin films show no evidence of solvent-related carbon species, with absorbances at ~2350 cm^{-1} corresponding to gaseous CO_2. b,c) To ensure signal from any trapped solvent molecules or carbon residues were readily detectable in these films a small volume of THF was drop cast on the film surface followed by immediate measurement ("THF soak"). This experiment was performed on both random and 001-oriented films to remove any further ambiguity from morphology dependent solvent evaporation/residue phenomena. The addition of THF resulted in a clear doublet signal between 2850–2980 cm^{-1}. This doublet peak diminishes back to the baseline as the THF solvent slowly evaporates (over several minutes). These results demonstrate that THF readily evaporates from the BiOI films and does not leave behind organic residues. These data confirm that any detectable carbon-species are purely adventitious. As such, this solution-process method allows for carbon-free BiOI thin films.

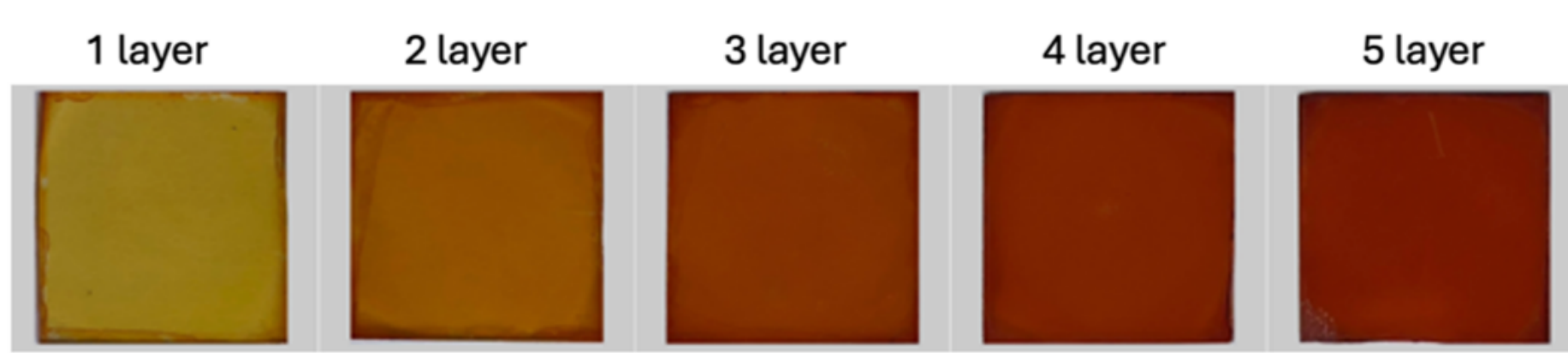

Figure S2: Optical images of 100 mg/mL BiOI thin films with increasing thickness.

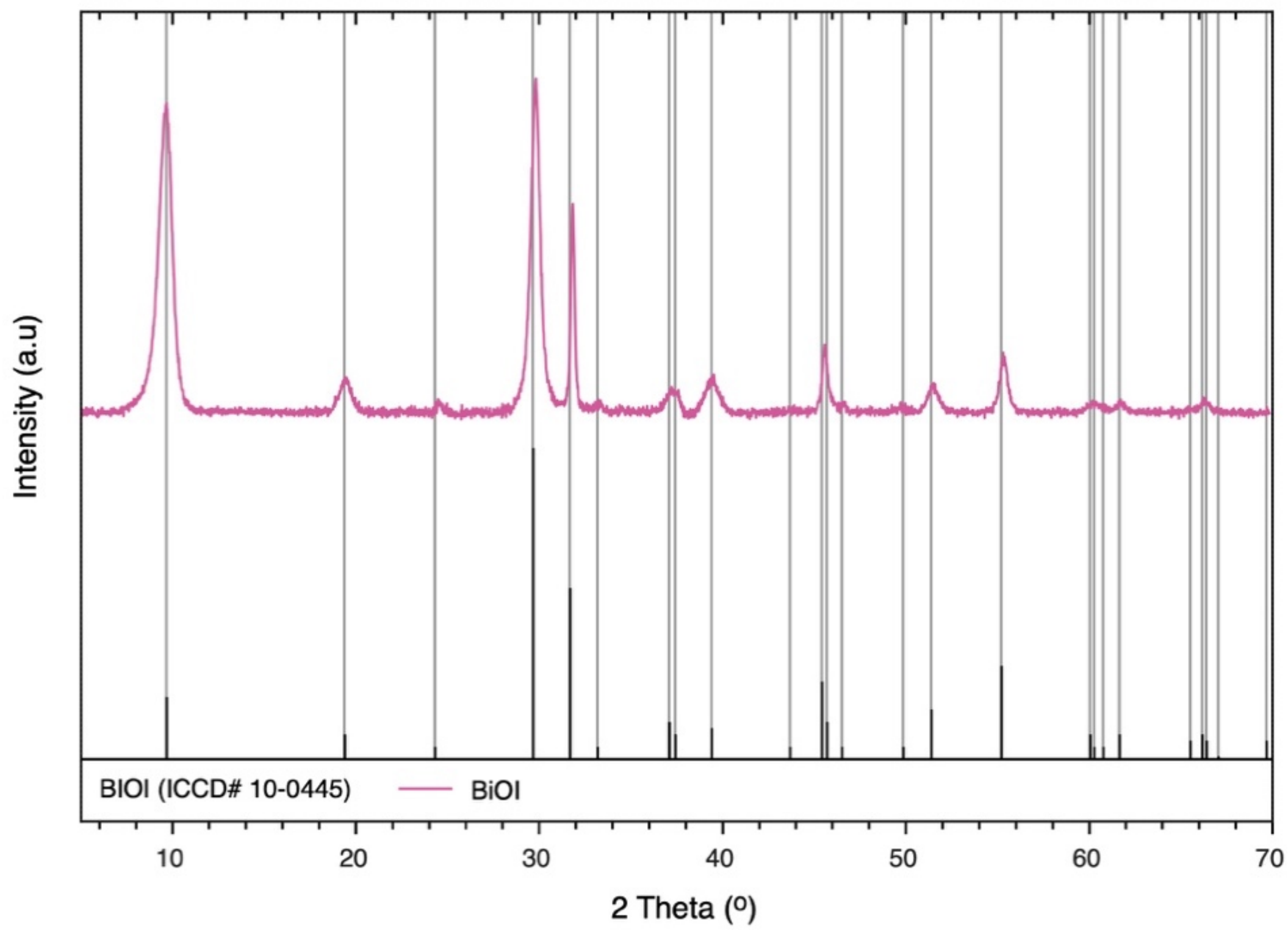

Figure S3: Characterisation BiOI thin films. The XRD is taken from a typical 3L BiOI thin film (120 mg/mL precursor). The pattern matches tetragonal (P4/nmm) BiOI (ICDD# 10-0445) with no residual BiI_3 peaks present.

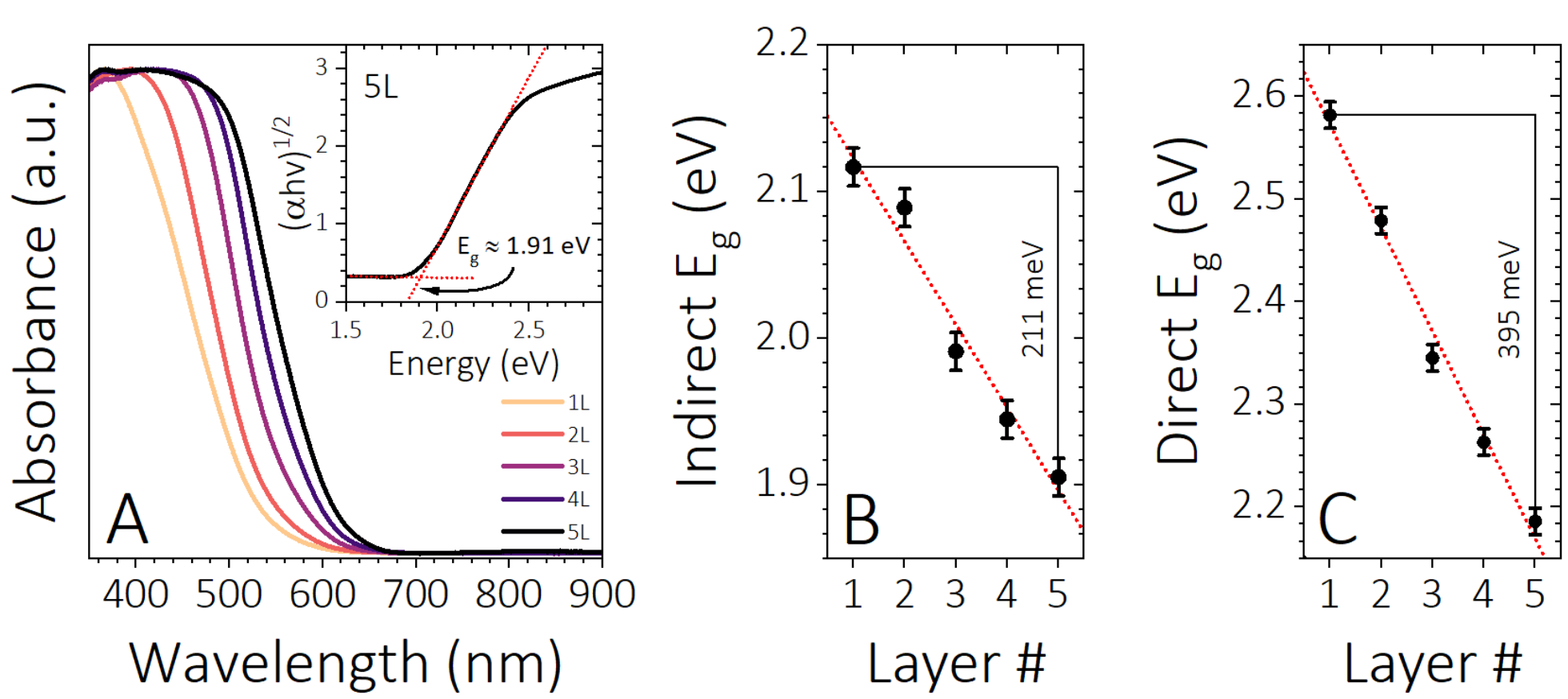

Figure S4: a) UV-Visible absorbance spectra of BIOI thin films as a function of film thickness (1–5 layers, from 100 mg/mL BiI_3). The absorbance profile of BiOI shows a gradual onset at approximately 600 nm typical of BIOI. Tauc analysis of BiOI using $(\alpha hv)^{1/r}$. A reduction in both (b) allowed indirect (r = 2) and (c) allowed direct (r = ½) band gaps was observed when increasing film thickness and a bulk indirect band gap of ~1.91 eV was estimated.

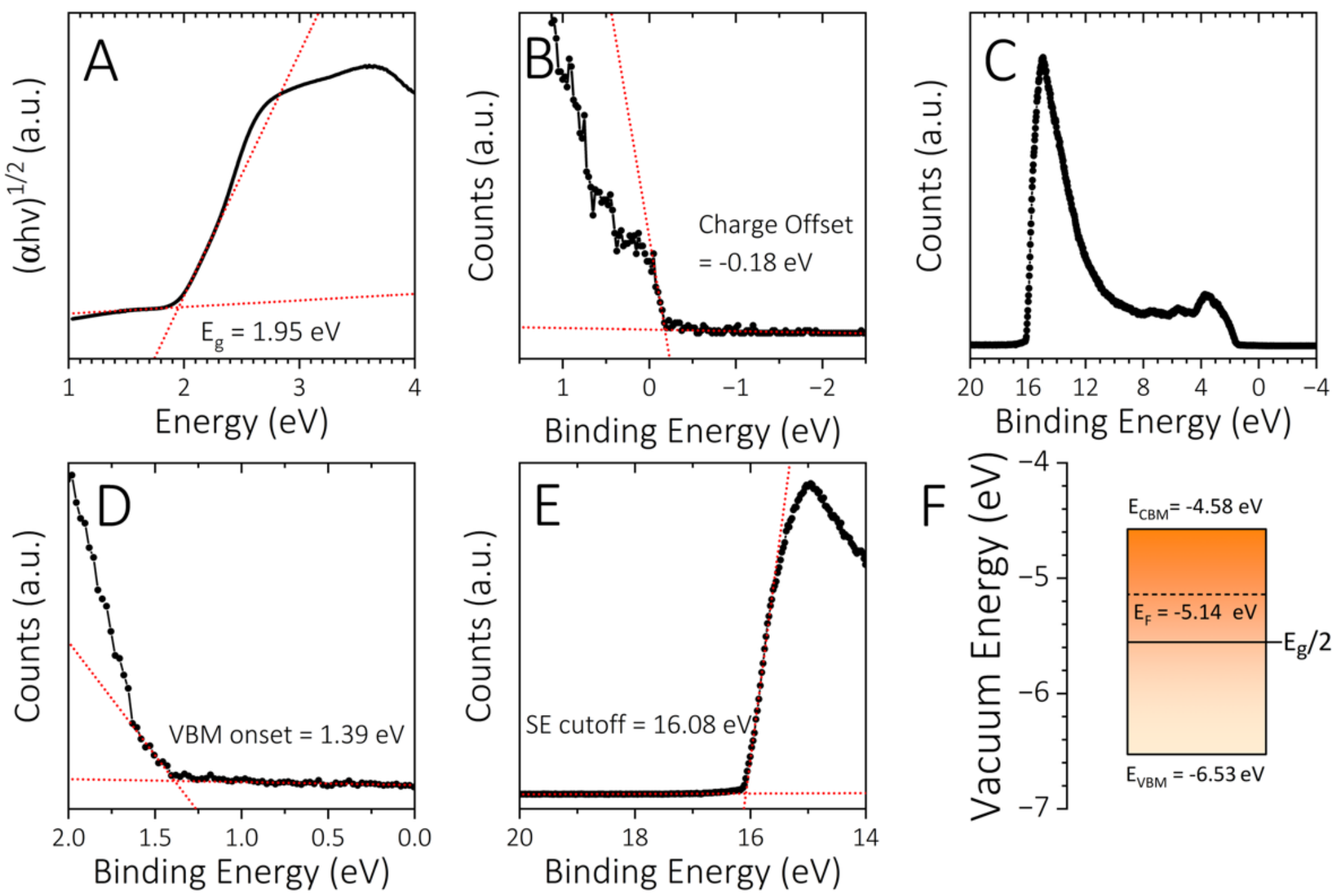

Figure S5: Band diagram characterisation of a three-layer 100 mg/mL BiOI sample. a) Tauc analysis of BiOI prepared on glass/TiO_2. b) UPS spectra of Au-spot, used for energy scale calibration. c) Charge-corrected UPS spectra of BiOI sample as prepared on Si/Au substrate. d,e) Magnified UPS spectra regions of interest, with linear fits included. f) Band diagram of as-prepared BiOI, on Si/Au substrate.

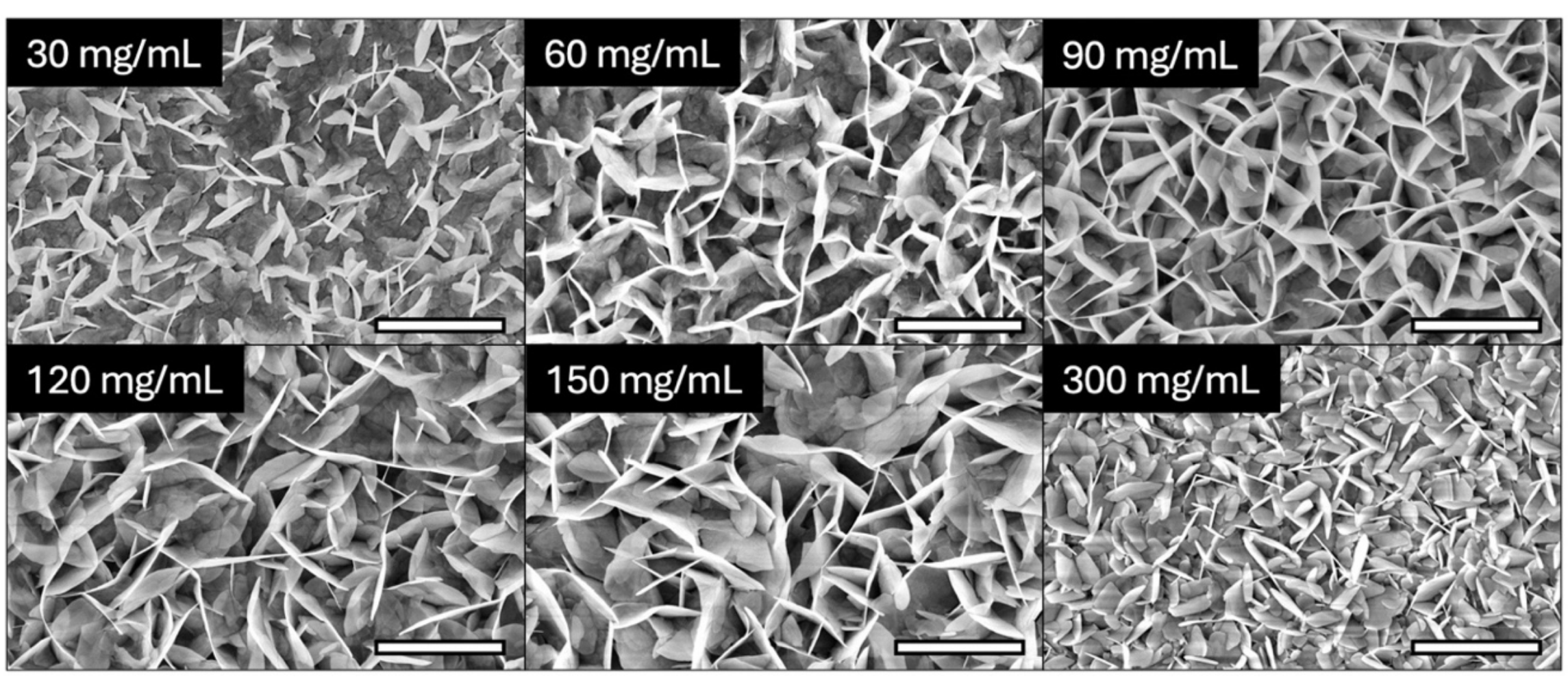

Figure S6: Top view SEM images of 3L BiOI cast from increasing [BiI_3] precursor between 30-300 mg/mL (scale bars are 1μm).

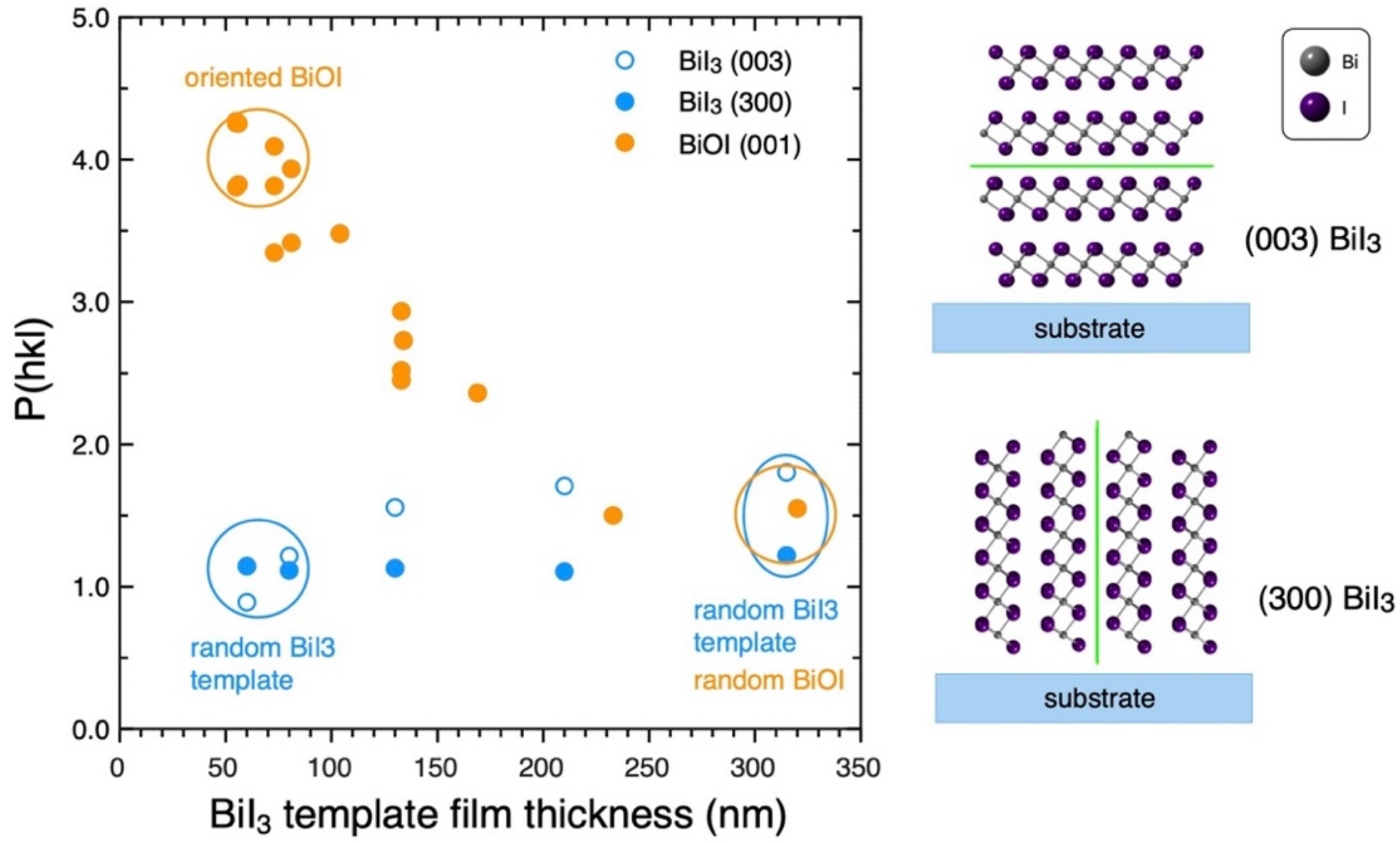

Figure S7: BiI_3 to BiOI orientation as a function of template thickness. BiI_3 were indexed to rhombohedral (R-3 space group) BiI_3 (ICDD# 48-1795). Texture coefficient was evaluated from n=5 peaks. Specifically, (003), (101), (113), (300), and (303). BiI_3 adopts a near random orientation within the thickness range investigated here, with only a very slight (003) orientation preference for the thickest template films. The data clearly show that the randomly oriented BiI_3 generates highly oriented BiOI for the thinnest films and random BiOI for thick films. As such, the orientational preference of BiOI is not correlated to the orientation of the template layer- ruling out topotactic conversion.

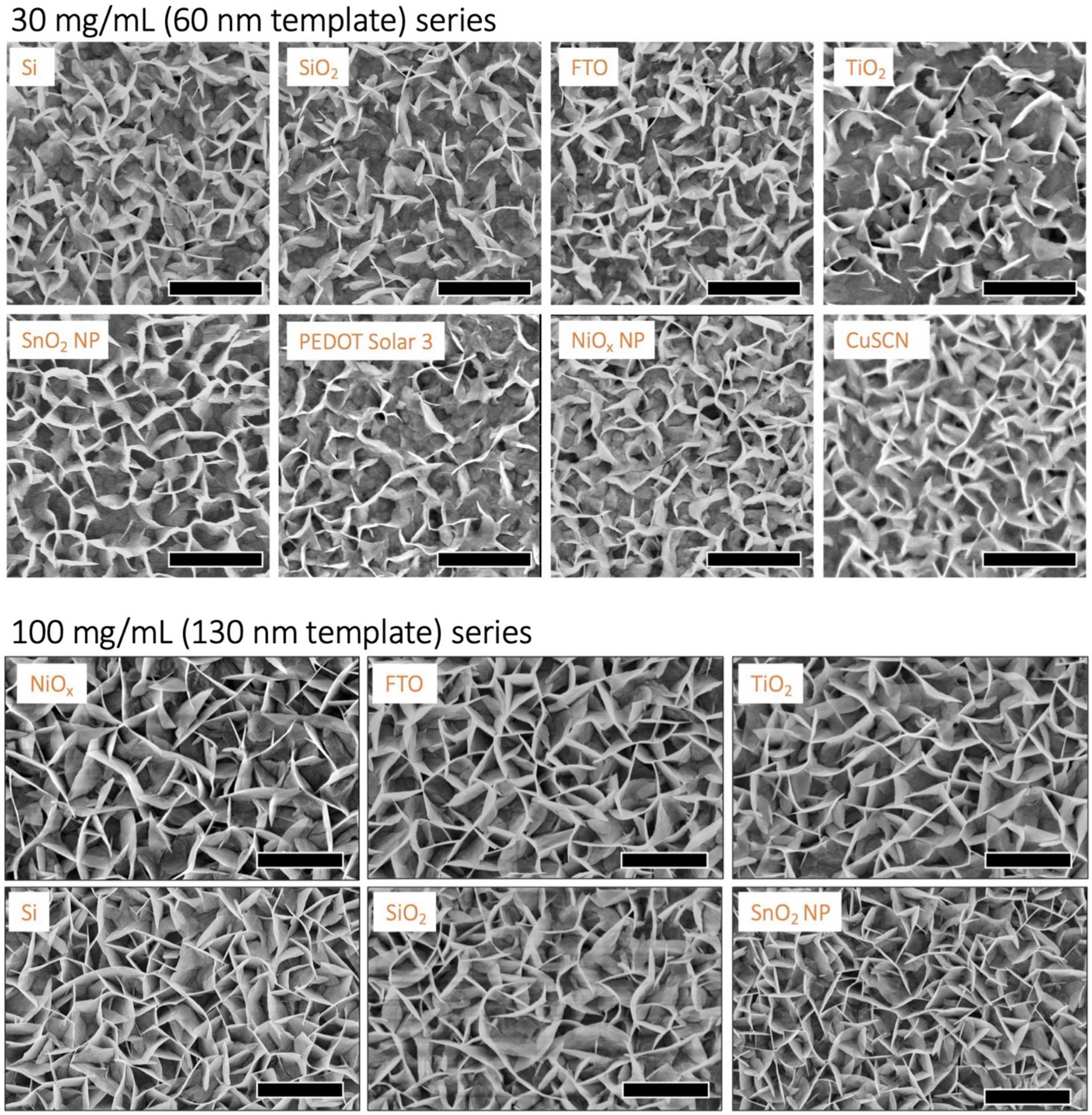

Figure S8: Top-down SEM images of BiOI (three-layers) on different substrates. Samples on various substrates for two different template thicknesses is shown. Scale bars are 2µm for the 60 nm template series and 1µm for the 130 nm template series.

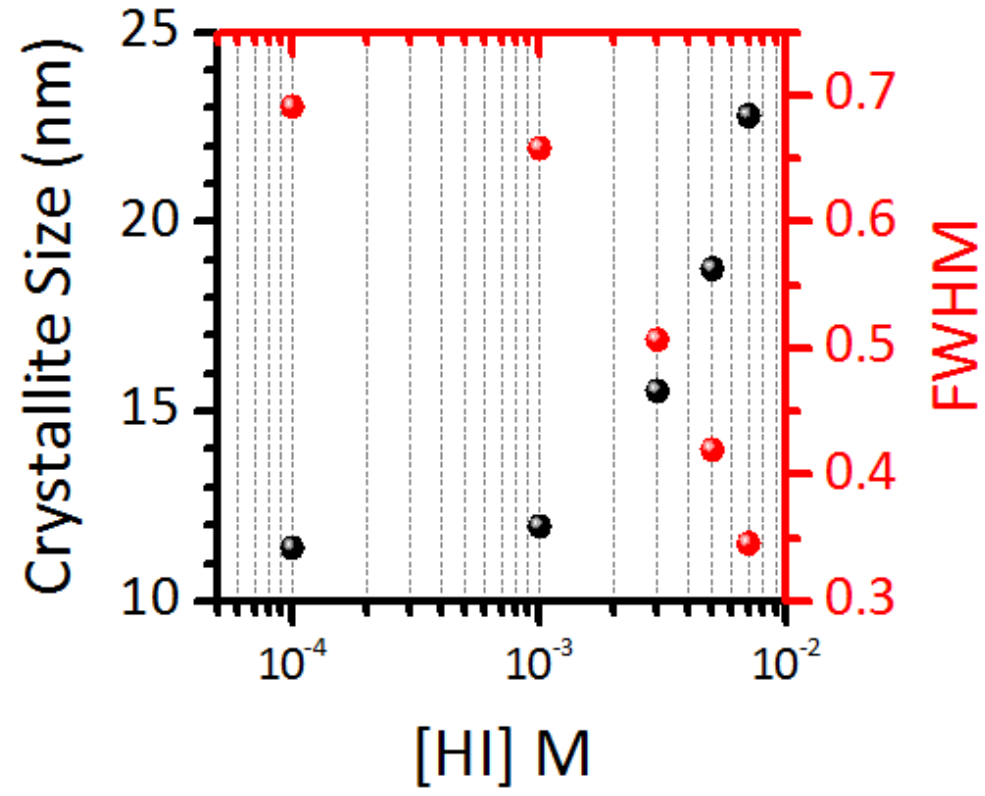

Figure S9: The Scherrer analysis of as prepared 3L 100 mg/mL (130 nm template) BiOI hydrolysed in various concentrations of HI in the conversion bath.

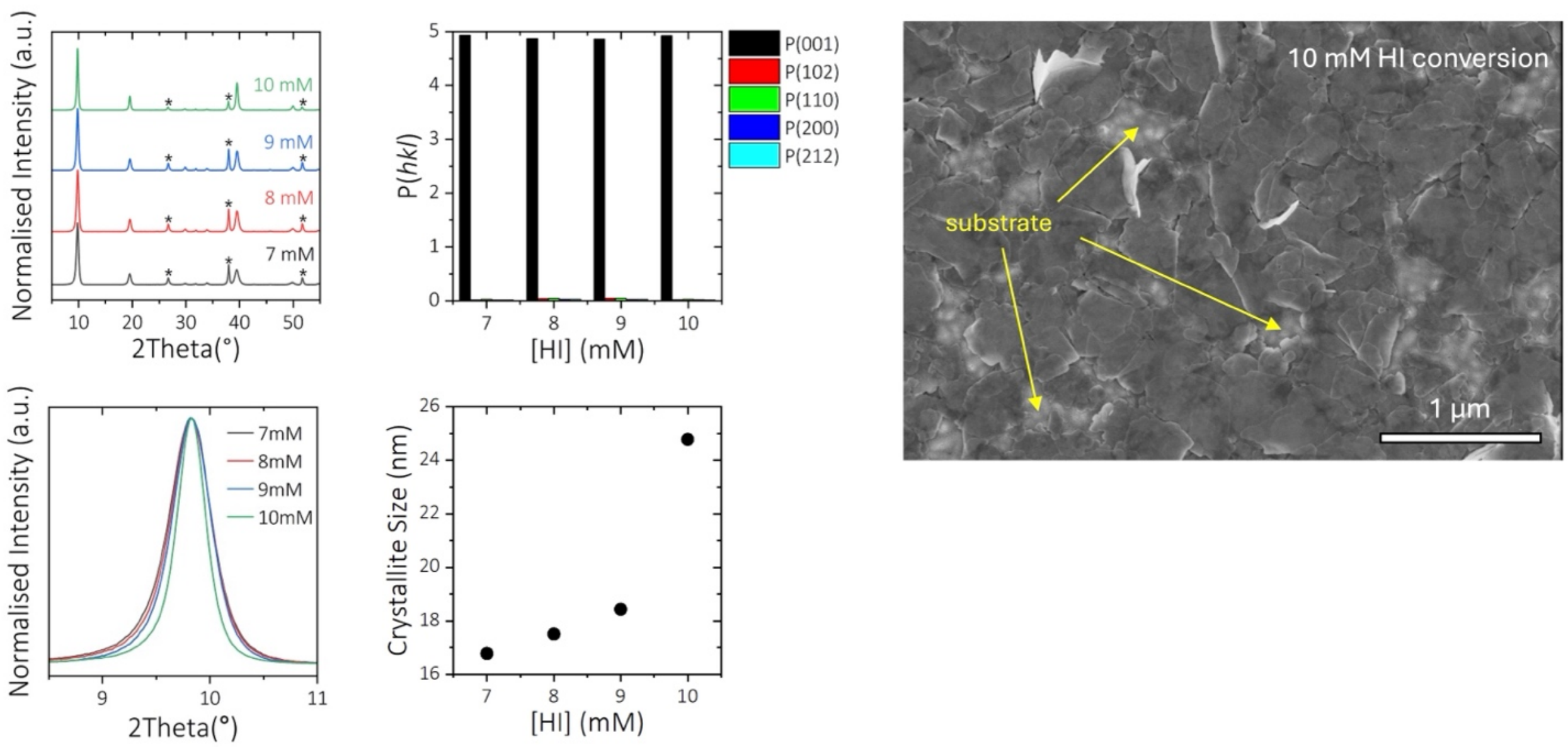

Figure S10: BiOI fabrication in conversion baths with [HI] from 7-10 mM. Within this range all films produce were 001 oriented (P(001>4.9). Increasing [HI] from 7 mM to 10 mM further narrows the 001 XRD peak (increasing average crystallite domain size). However, we found that specific (less controllable) parameters such as BiI_3 batch-to-batch variation, THF batch quality, lab-humidity when forming the BiI_3, precise measured HI concentration (also bottle age) all affected the results. Repeated experiments over long periods revealed that on some occasions we could obtain orientation and 001 BiOI thin films from 10 mM, while on other occasions this caused unacceptable film dissolution (with visible substrate domains difficult to fill via layer-by-layer). As such, we empirically arrived at the concentration of [HI]=7 mM as this reproducibly and reliably provided highly oriented 001 BiOI thin films regardless of all other factors - as required to provide a robust method to researchers.

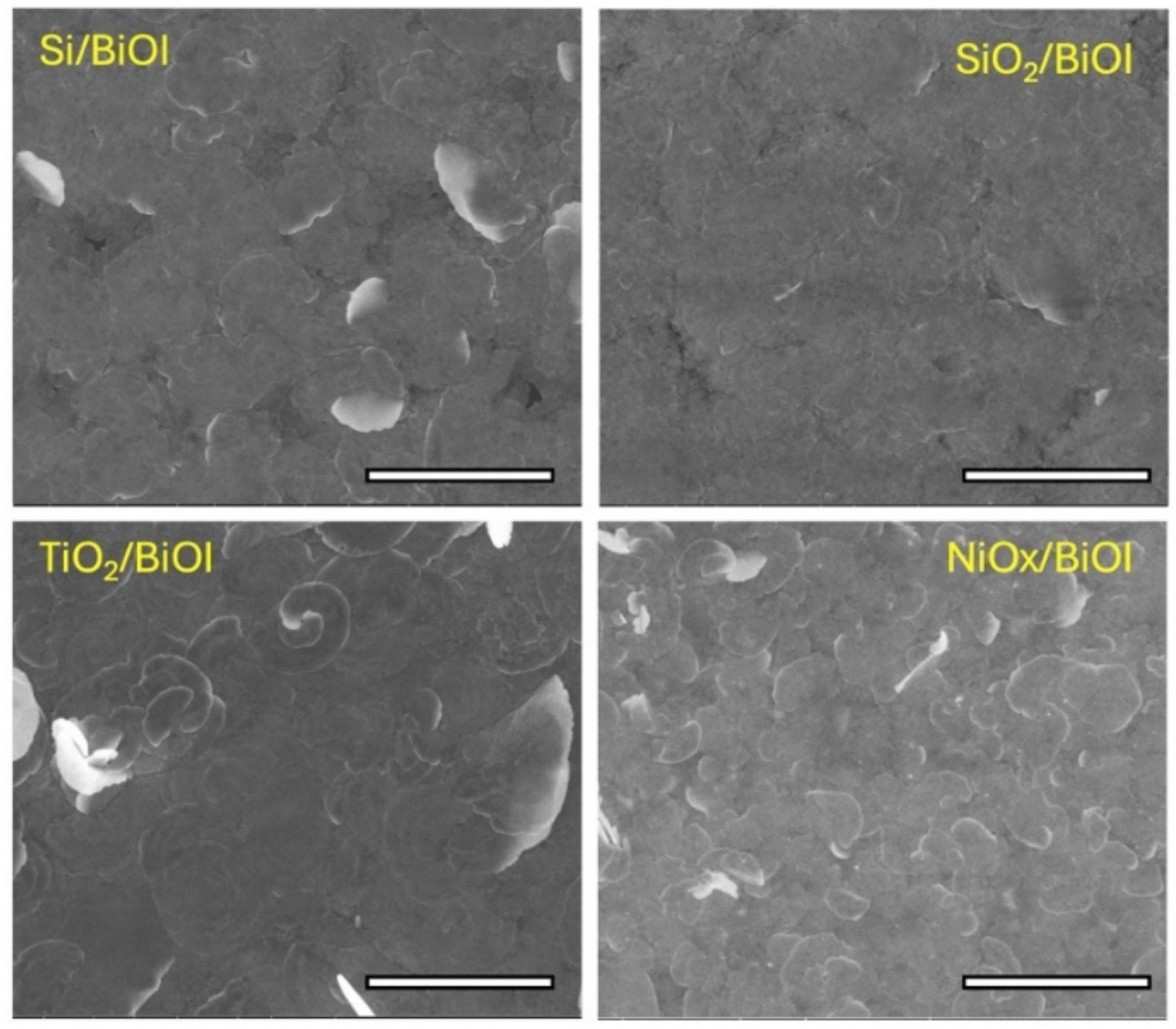

Figure S11: Top view SEM images of 001-BiOI on various device compatible substrates. Scale bar 2 um.

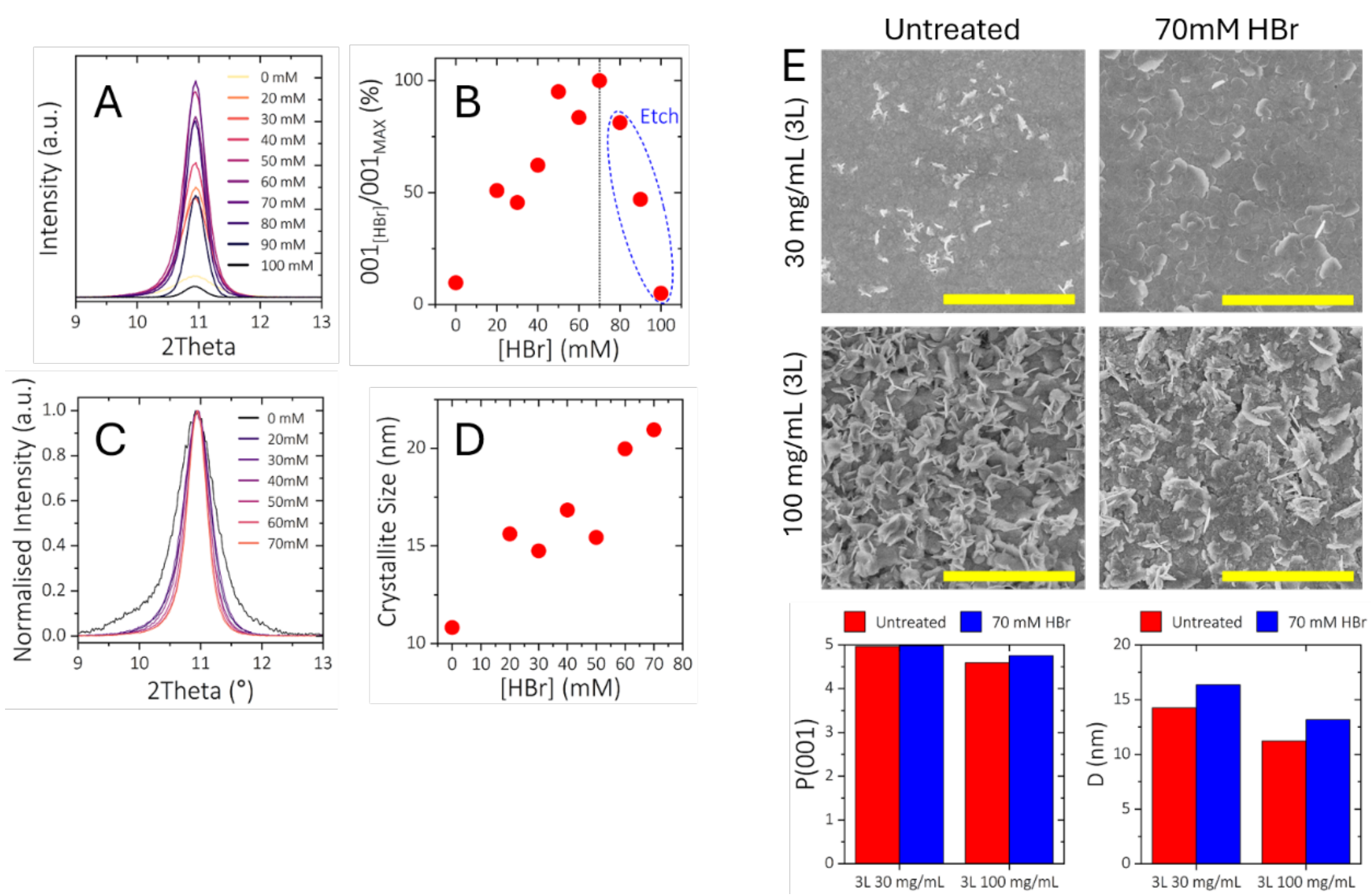

Figure S12: Halide-assisted orientation control of 001-BiOBr. a) The (001) diffraction peak and b) relative (001) intensity ratios with respect to [HBr], where 70 mM HBr is identified as optimal conditions. c) The normalised (001) diffraction peaks and d) corresponding crystallite sizes of 001-BiOBr with increasing [HBr]. e) SEM, P(001), and crystallite size comparisons of untreated and 70 mM HBr treated BiOBr thin films (3L, 30 mg/mL and 100 mg/mL). Although BiOBr is shown to begin (001) oriented for thinner films, there are minor but substantial improvements to morphology and texture as shown for thicker (3L, 100 mg/mL) films treated with 70 mM HBr, alongside increases to crystallite size (as similarly established for BiOI prepared with HI).

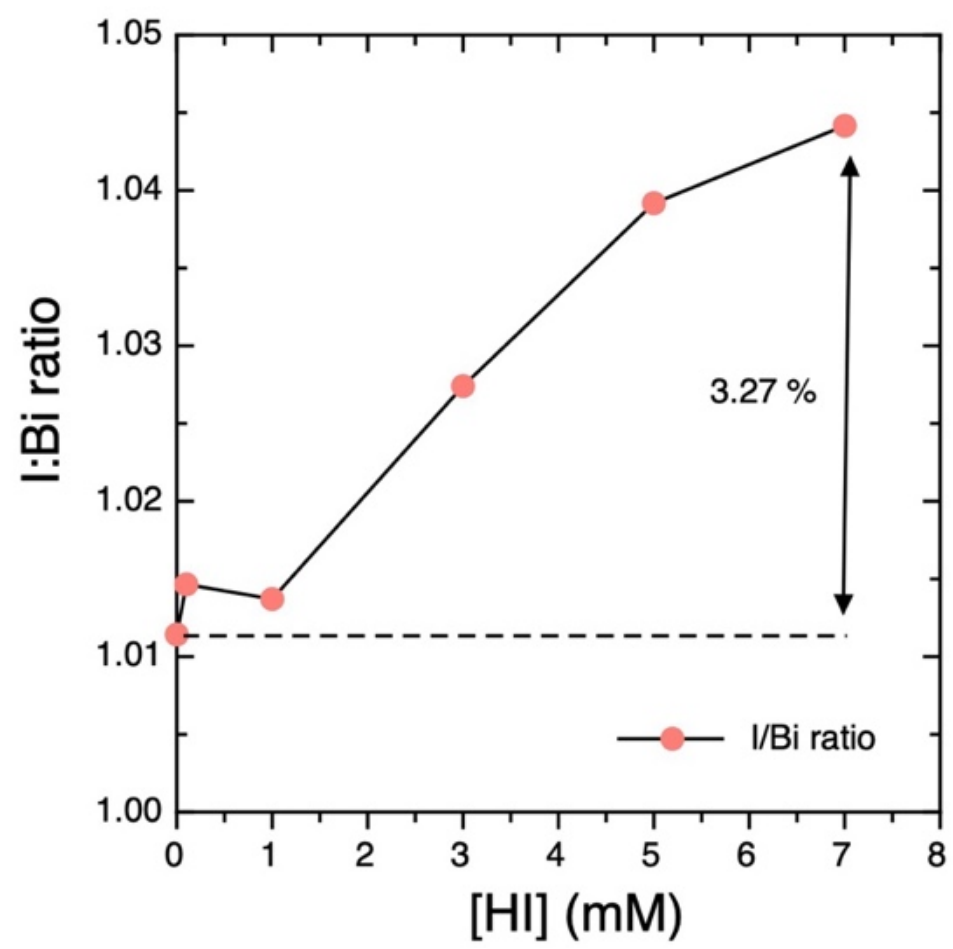

Figure S13: Elemental Analysis (SEM-EDS) of BiOI hydrolysed in various concentrations of HI in the conversion bath. Analysis was conducted on samples calibrated to BiI_3 powder (1:3).

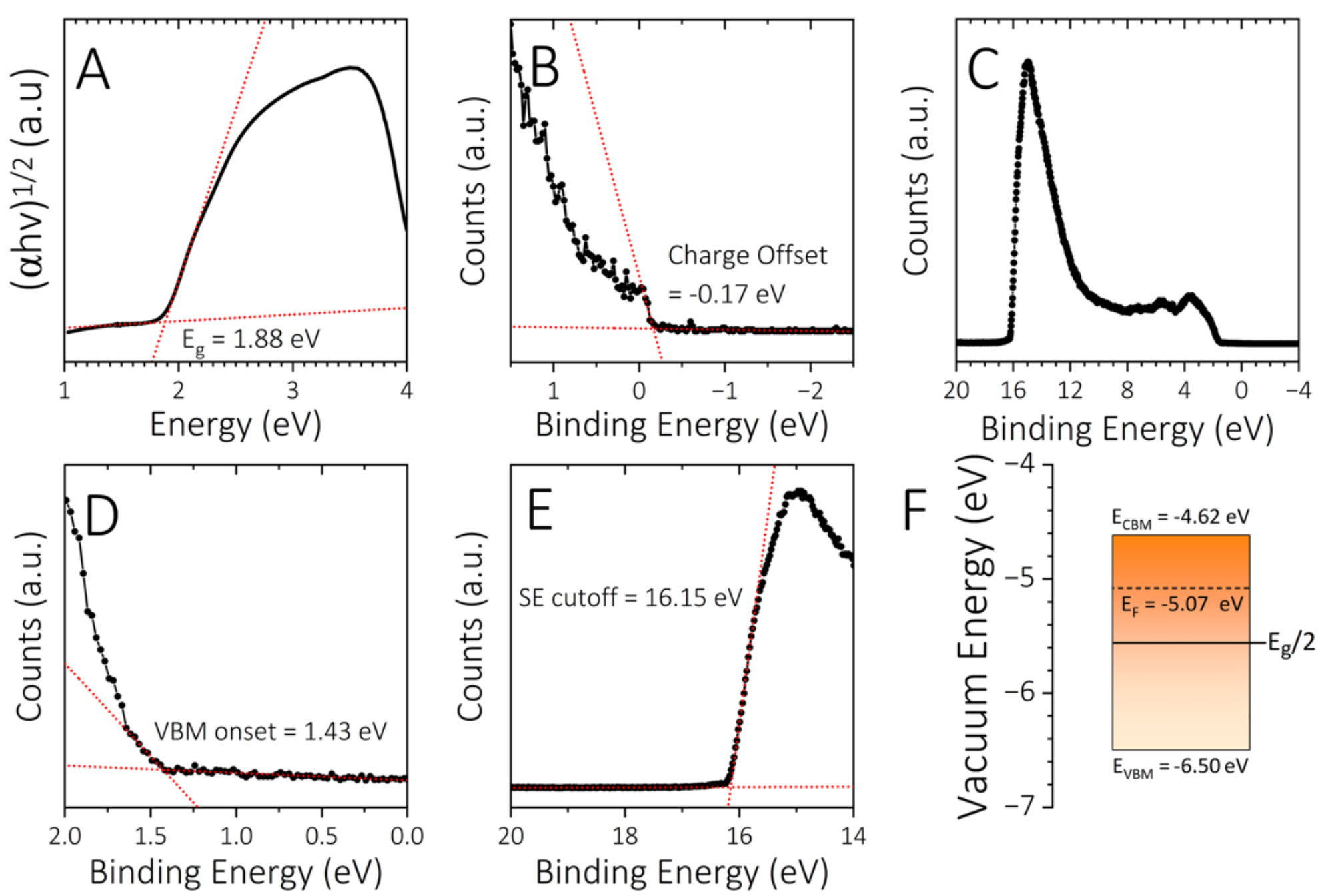

Figure S14: Band diagram characterisation of a three-layer 100 mg/mL 001-BiOI sample. a) The indirect Tauc analysis of 001-BiOI prepared on glass/TiO_2. b) UPS spectra of Au-spot, used for energy scale calibration. c) Charge-corrected UPS spectra of 001-BiOI sample, as-prepared on Si/Au substrate. d,e) Magnified UPS spectra regions of interest, with linear fits included. f) Band diagram of as prepared 001-BiOI, on Si/Au substrate.

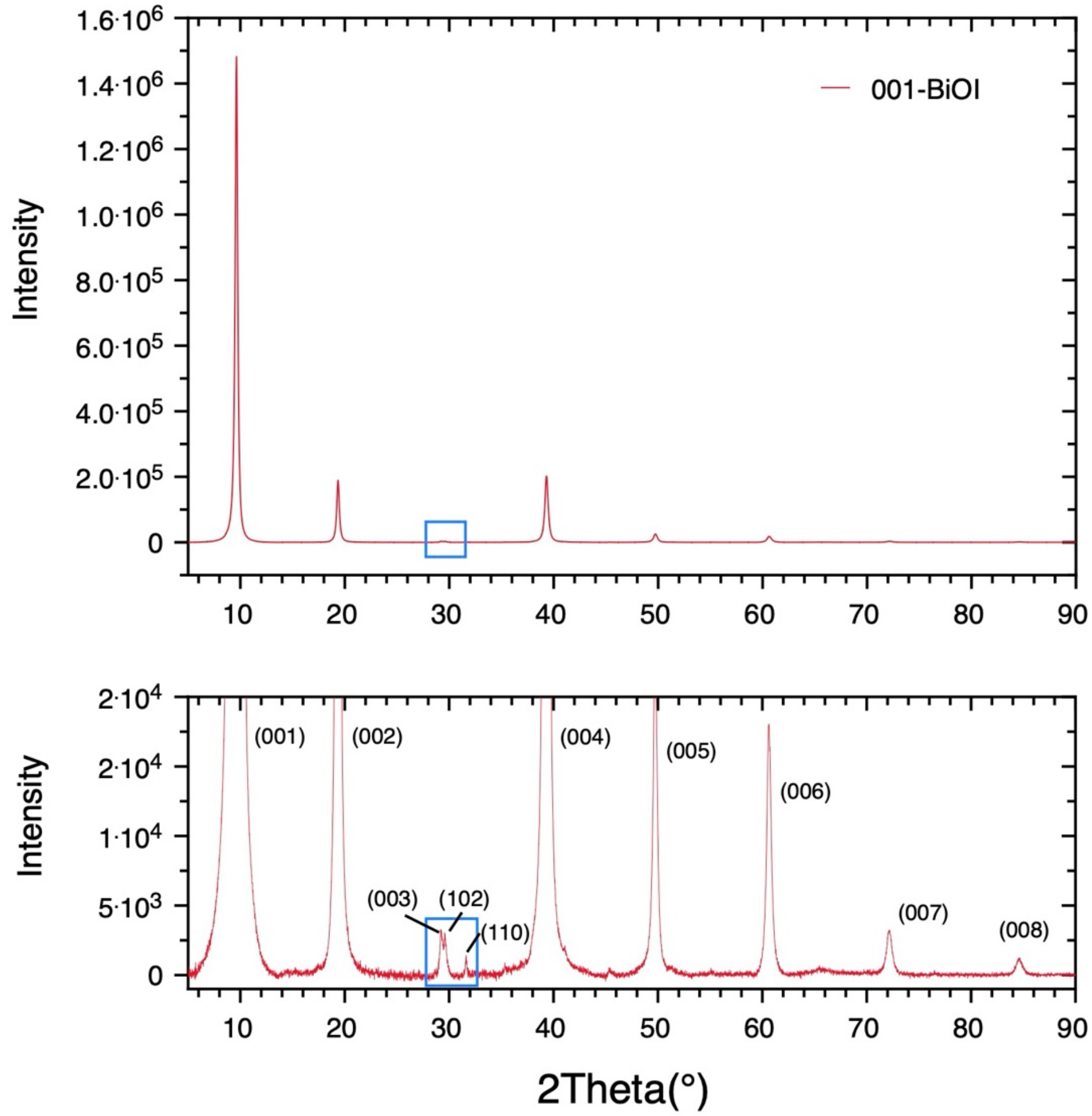

Figure S15: XRD of a typical highly oriented 001-BiOI thin film. The XRD is plotted on a linear scale to show the contributions from {00*l*} planes are dominant. Residual detectable peaks in the blue box are magnified in the lower panel and correspond to (102) and (110) reflections – although their intensities are barely detectable (even for long time scan XRD patterns) at ~0.1% of the (001) reflection.

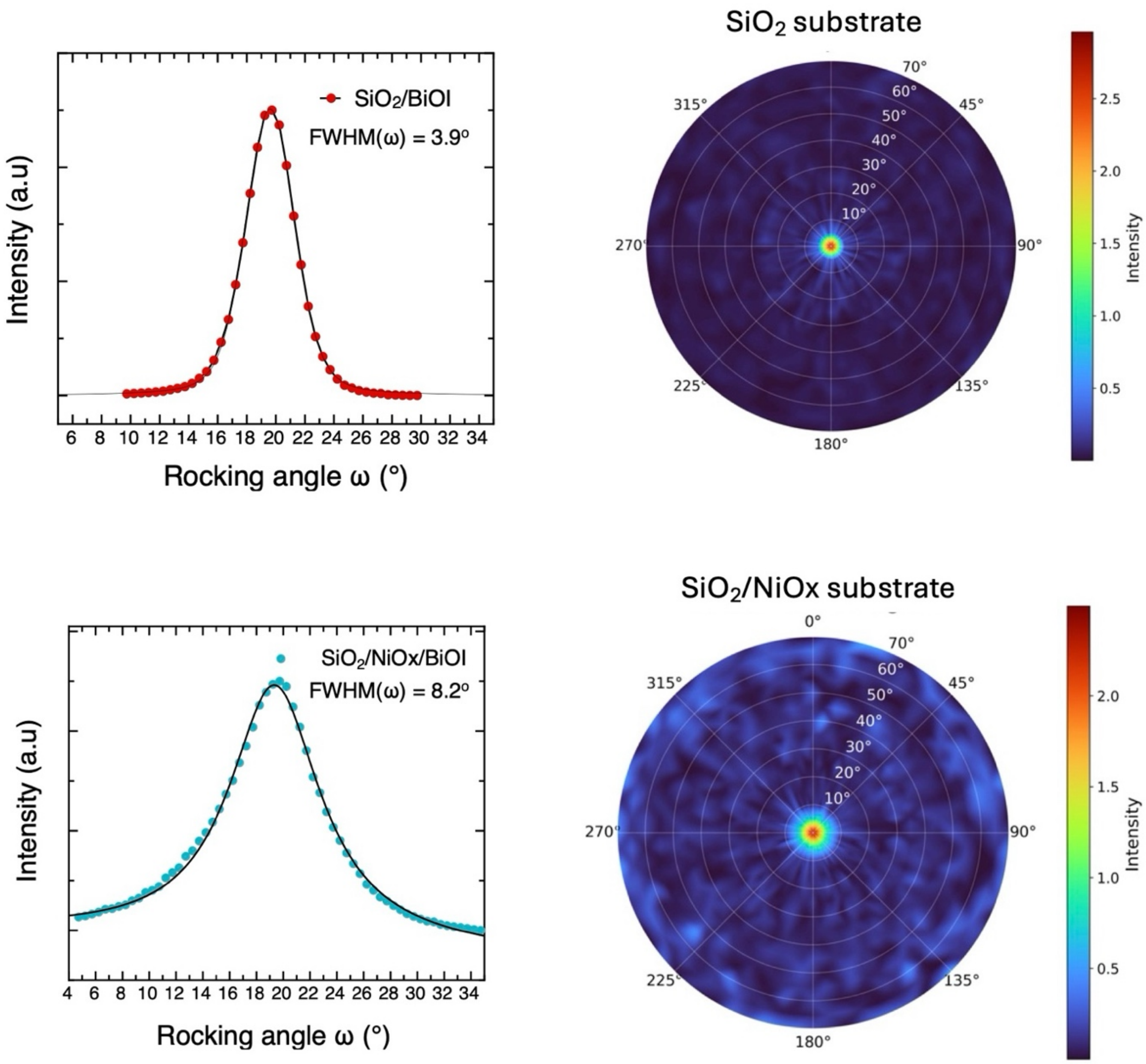

Figure S16: Pole figure and rocking-curve analysis of highly oriented 001-BiOI thin films deposited on SiO_2 (top panels) and SiO_2 incorporating a thin (~10 nm) NiOx interlayer (bottom panels). Pole figures measured using the BiOI (004) reflection show a highly concentrated central pole for both films, confirming strong preferential c-axis orientation normal to the substrate. The film deposited directly on SiO_2 exhibits a narrower angular intensity distribution, indicative of a more constrained crystallite orientation distribution. Correspondingly, Voigt fits to the BiOI (004) rocking curves yielded FWHM values of 3.9° (RMS tilt ≈ 1.7°) for SiO_2 and 8.2° (RMS tilt ≈ 3.5°) for SiO_2/NiOx, indicating a substantially broader crystallite tilt distribution and increased mosaic spread in the presence of the NiOx interlayer.

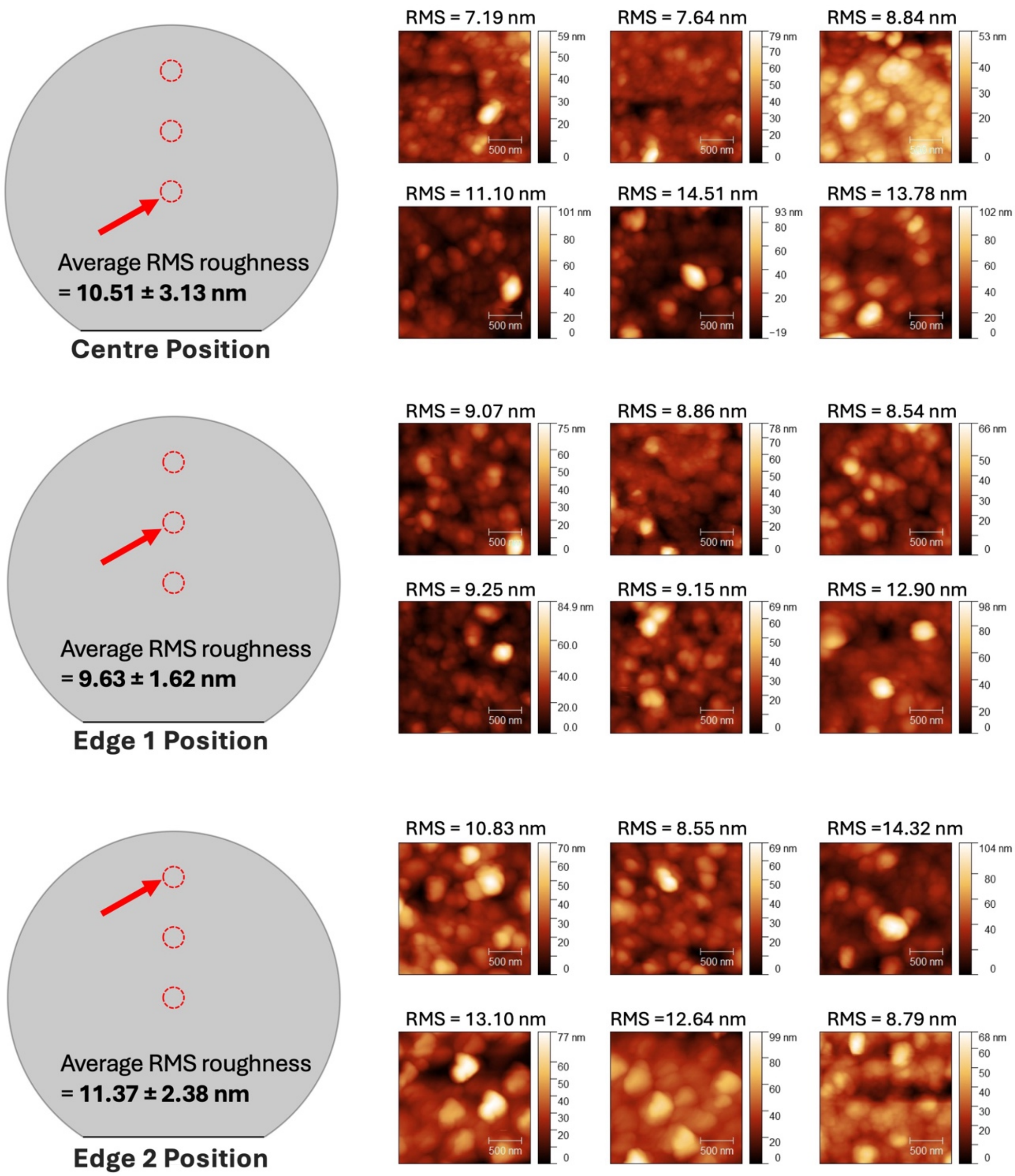

Figure S17: AFM characterization of BiOI films measured at multiple locations across the wafer. Representative AFM images acquired at the wafer centre and edge positions reveal similar surface morphologies with no obvious spatial variation in grain structure or topography. Statistical analysis of six measurements at each location yielded RMS roughness values of 10.51 ± 3.13 nm (centre), 9.63 ± 1.62 nm (edge1), and 11.37 ± 2.38 nm (edge2), corresponding to an overall wafer-average roughness of approximately 10.5 nm. One-way ANOVA analysis indicated no statistically significant difference in roughness between measurement locations ($p = 0.49$), demonstrating good wafer-scale uniformity in surface morphology.

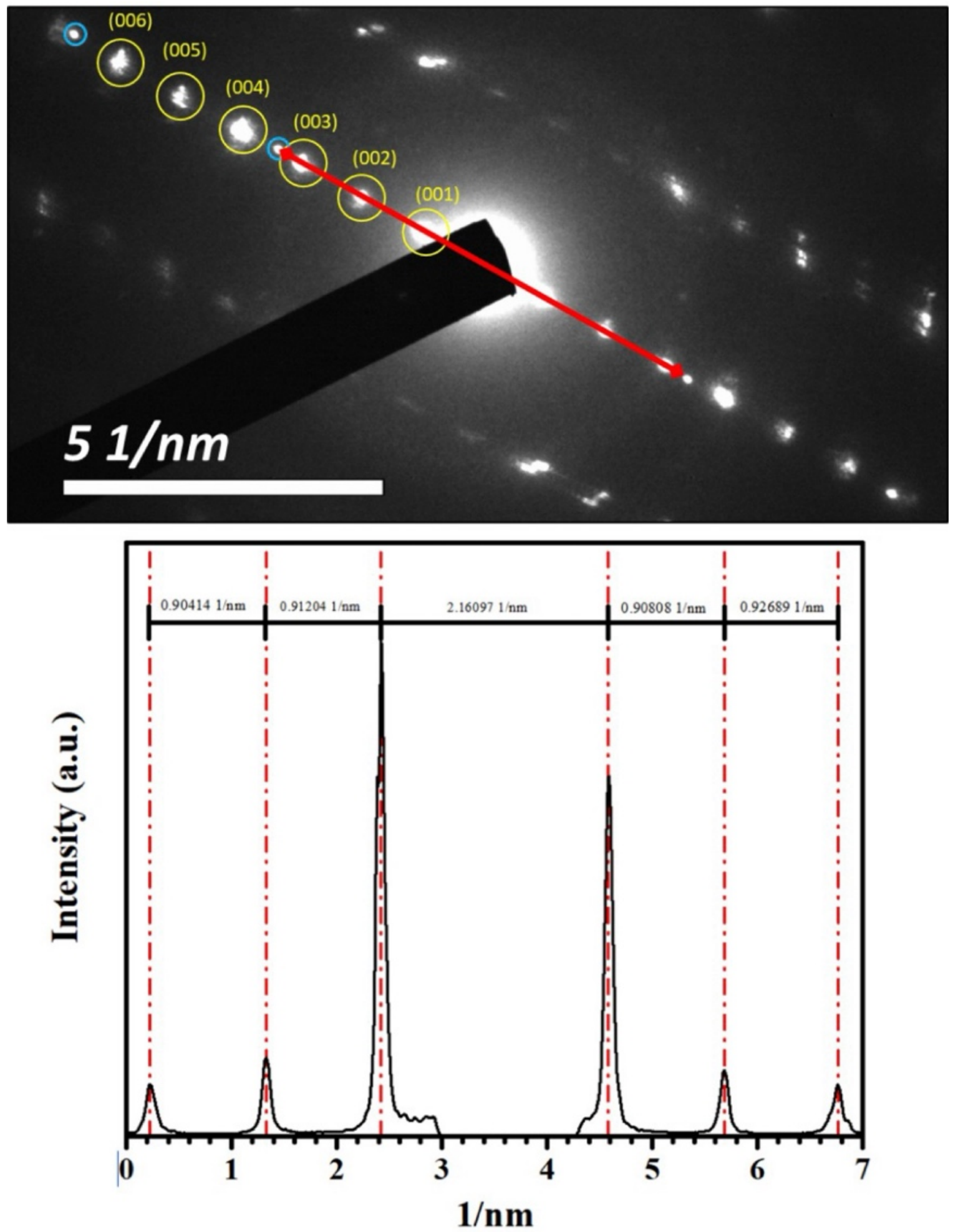

Figure S18: The SAED pattern (top) of three-layer 30 mg/mL 001-BiOI, with included line intensity profile (bottom) extracted from the corresponding red line for d-spacing determination. {00*l*} BiOI reflections are indexed, with substrate reflections circled in blue.

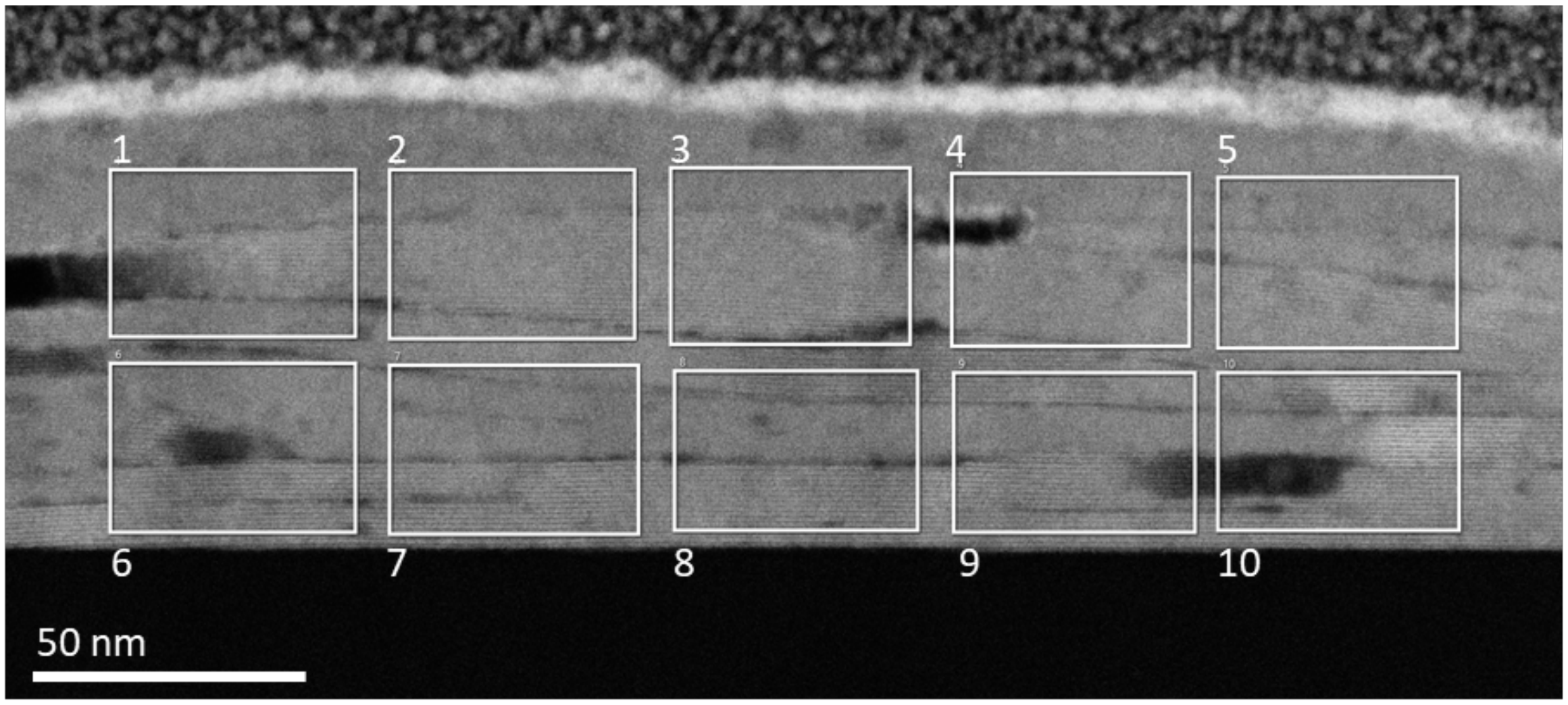

Statistic	O	I	Bi
Max	38.91	35.12	36.05
Min	31.01	30.26	29.50
Average	34.73	32.94	32.33
Standard Deviation	2.63	1.69	1.84

Figure S19: Elemental Analysis (STEM-EDS) of three-layer 30 mg/mL 001-BiOI cross-section, with included atomic percentage statistics shown in the attached table.

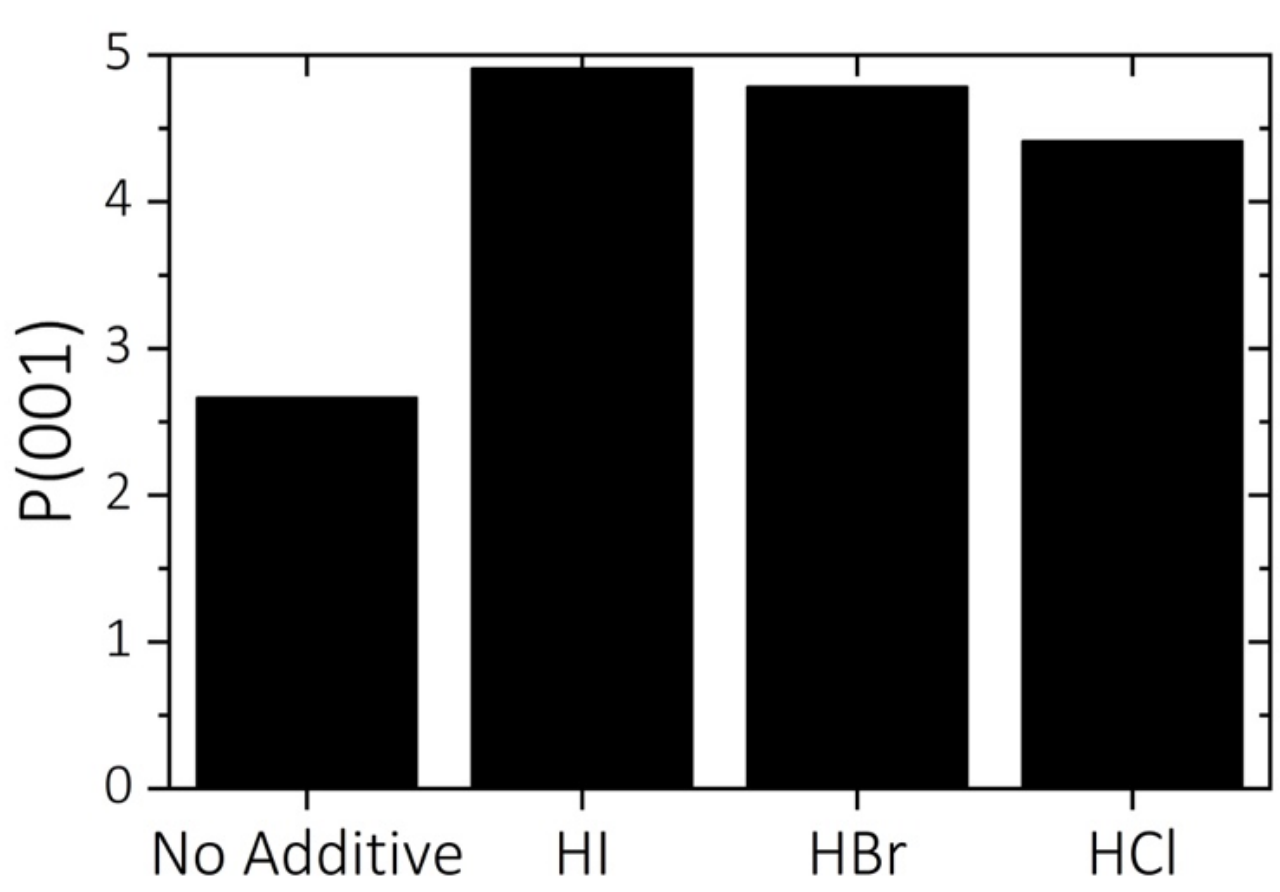

Figure S20: Data from 3L (100 mg/mL) templated films converted in different hydrohalic acids at 7 mM. All hydrohalic acids have orienting effects. From repeated experiments the (001) orientation is highest when using HI, then HBr and then HCl (I>Br>Cl).

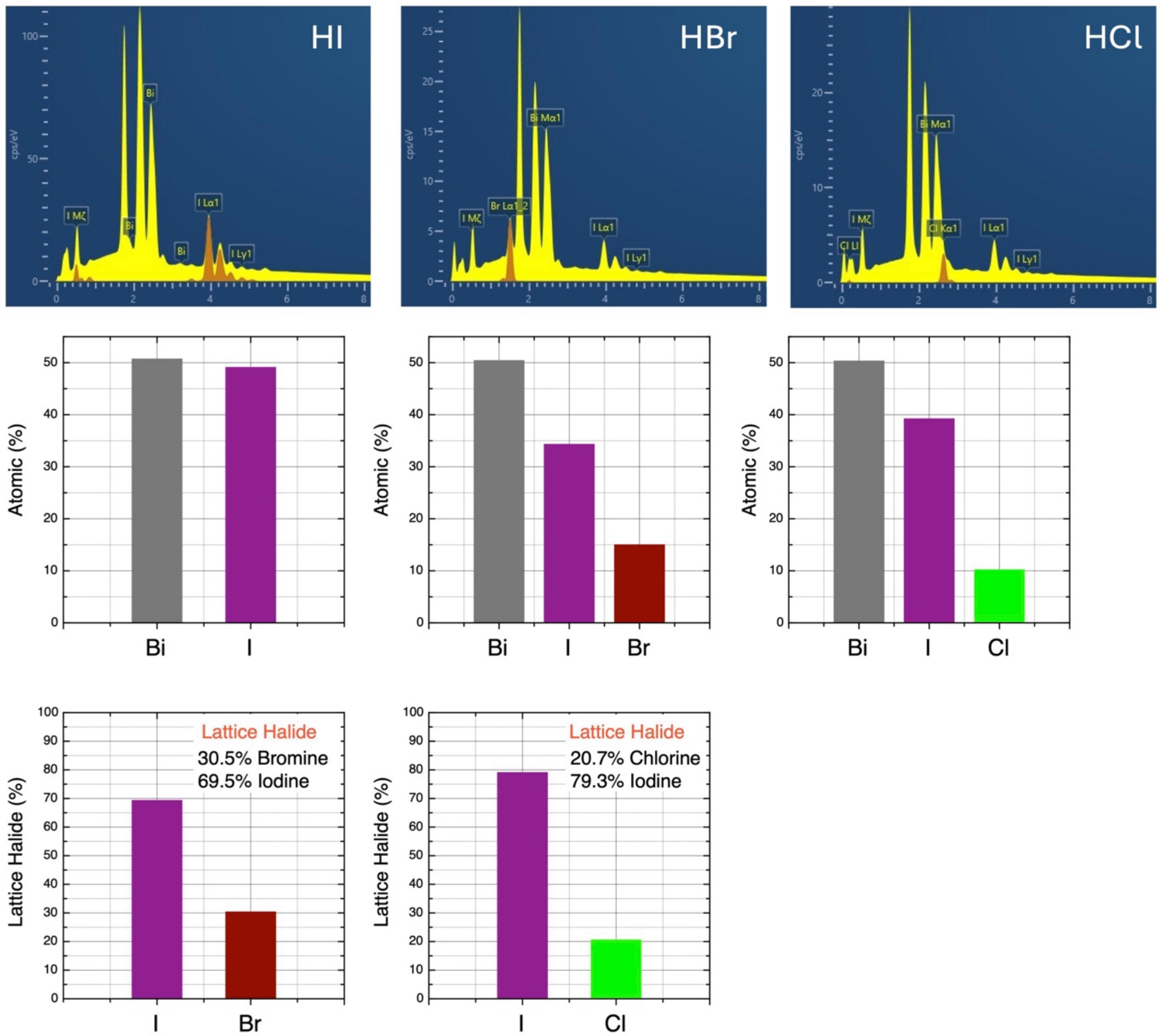

Figure S21: Raw EDS results from films oriented using HI, HBr, and HCl (top row). Calculated Atomic % of key elements in the film (middle row). Calculated % lattice halide (bottom row) showing that films oriented in HBr have ~30% Br substitution and films oriented in HCl have ~20% Cl substitution. Collectively these data show that bromine substitutes approximately 1/3 of the lattice halide sites and Chlorine ~1/5. This confirms that these acids liberate anions to form mixed halide $BiI_y(X_{1-y})$ compounds during BiI_3 hydrolysis.

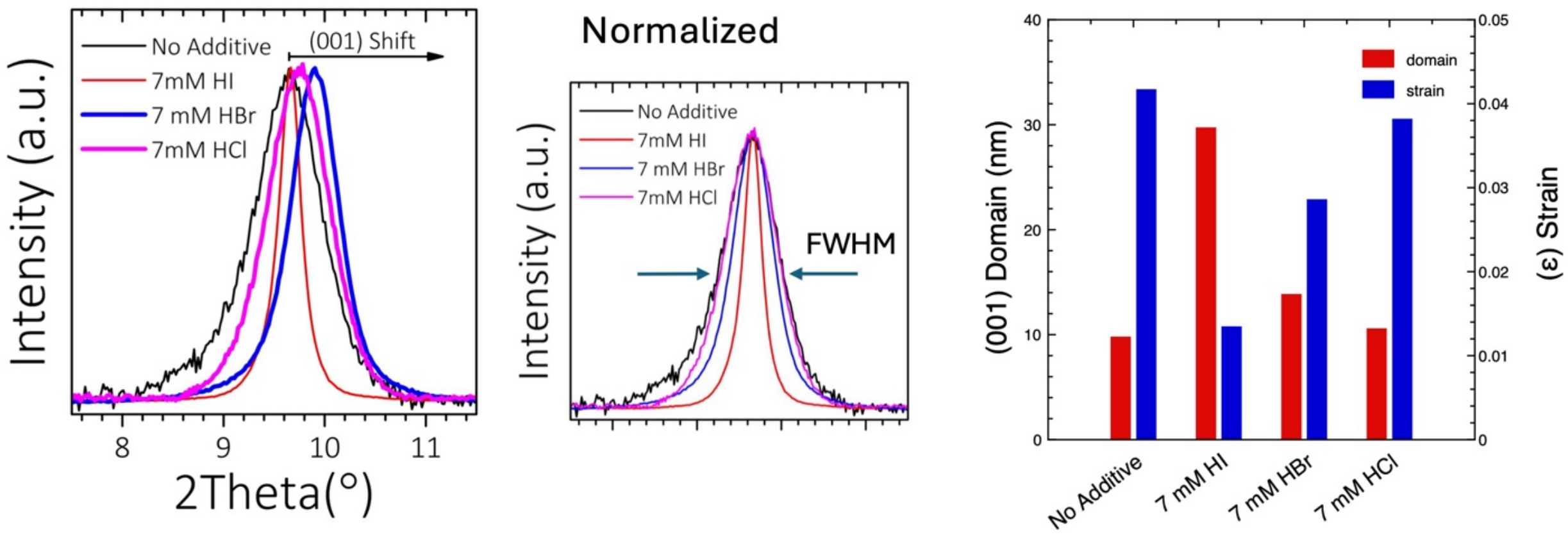

Figure S22: (Left) XRD patterns of the (001) peak for conversions of 3L (100 mg/mL) films in different hydrohalic acids. We can see that conversions using HBr and HCl result in a peak shift consistent with halide incorporation in the lattice (S12). (Middle) From the normalized patterns sequentially broader (001) XRD peaks for HI (0.265°), HBr (0.568°), and HCl (0.743°) can be seen. The is reinforced by the domain size and strain data (Right). These data show vastly improved domain size for iodine anions that follow the trend (I>Br>Cl). Likewise, the highest strain is found when using chloride anions (HCl) in the conversion bath, which is close to the strain seen for the samples without additive.

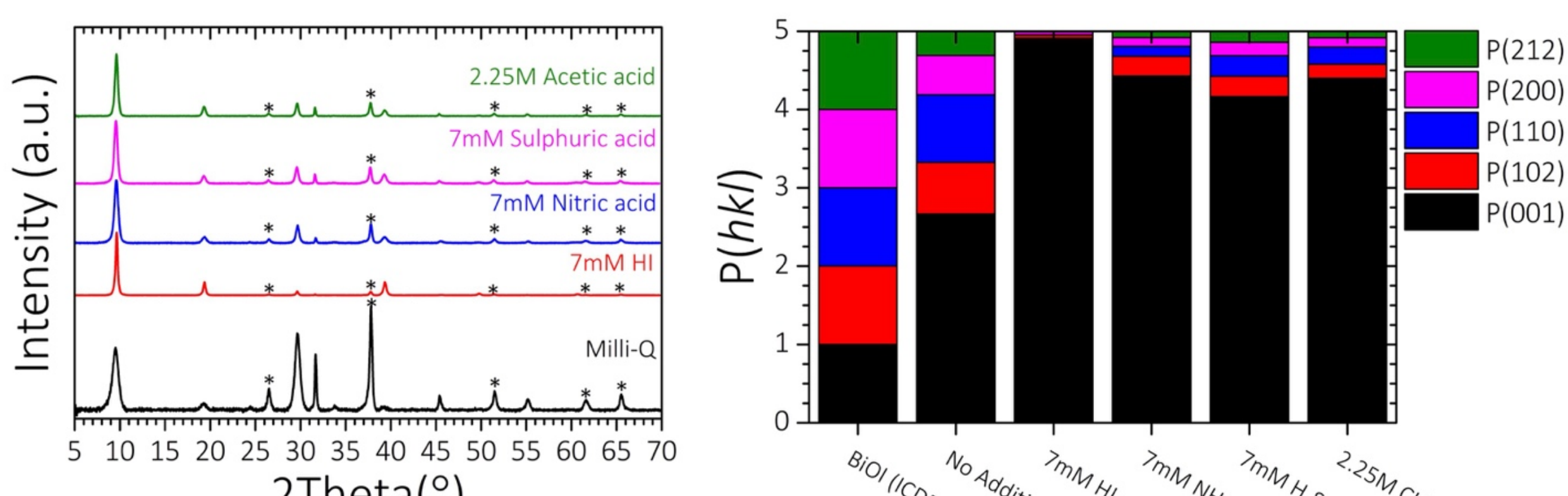

Figure S23: XRD patterns (left) and the corresponding texture coefficients (right) for 3L 100 mg/mL samples converted in different non-hydrohalic acids (with HI and pure water controls shown). FTO substrate peaks are asterisked. All acids are normalised to pH~2.

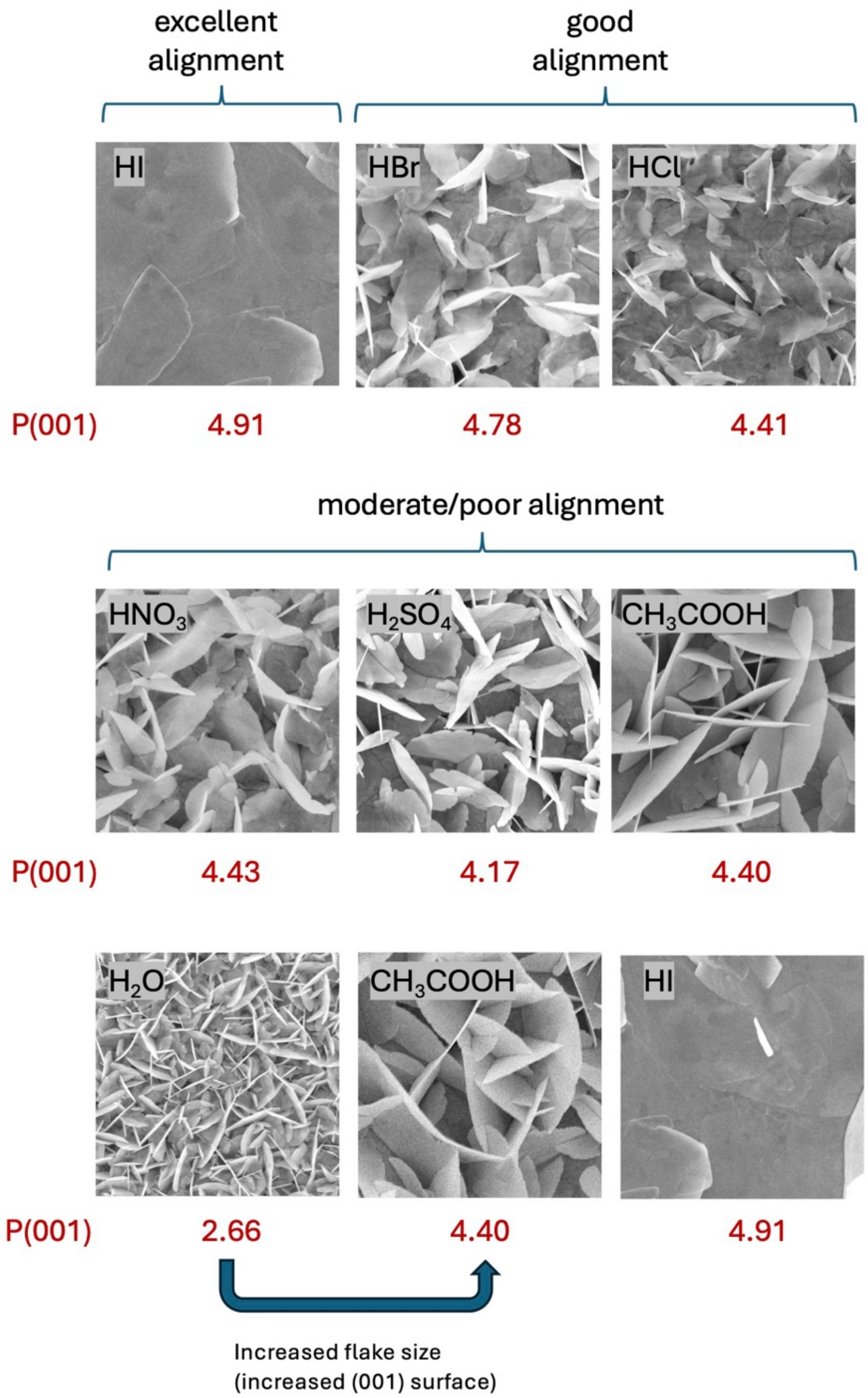

Figure S24: All 3L 100 mg/mL converted BiOI films with different types of acids in the conversion bath. All acids normalised to pH 7, with acetic acid at 2.25 M and the other acids at 7 mM. The most effective orientation occurs when using iodine (HI) additive.

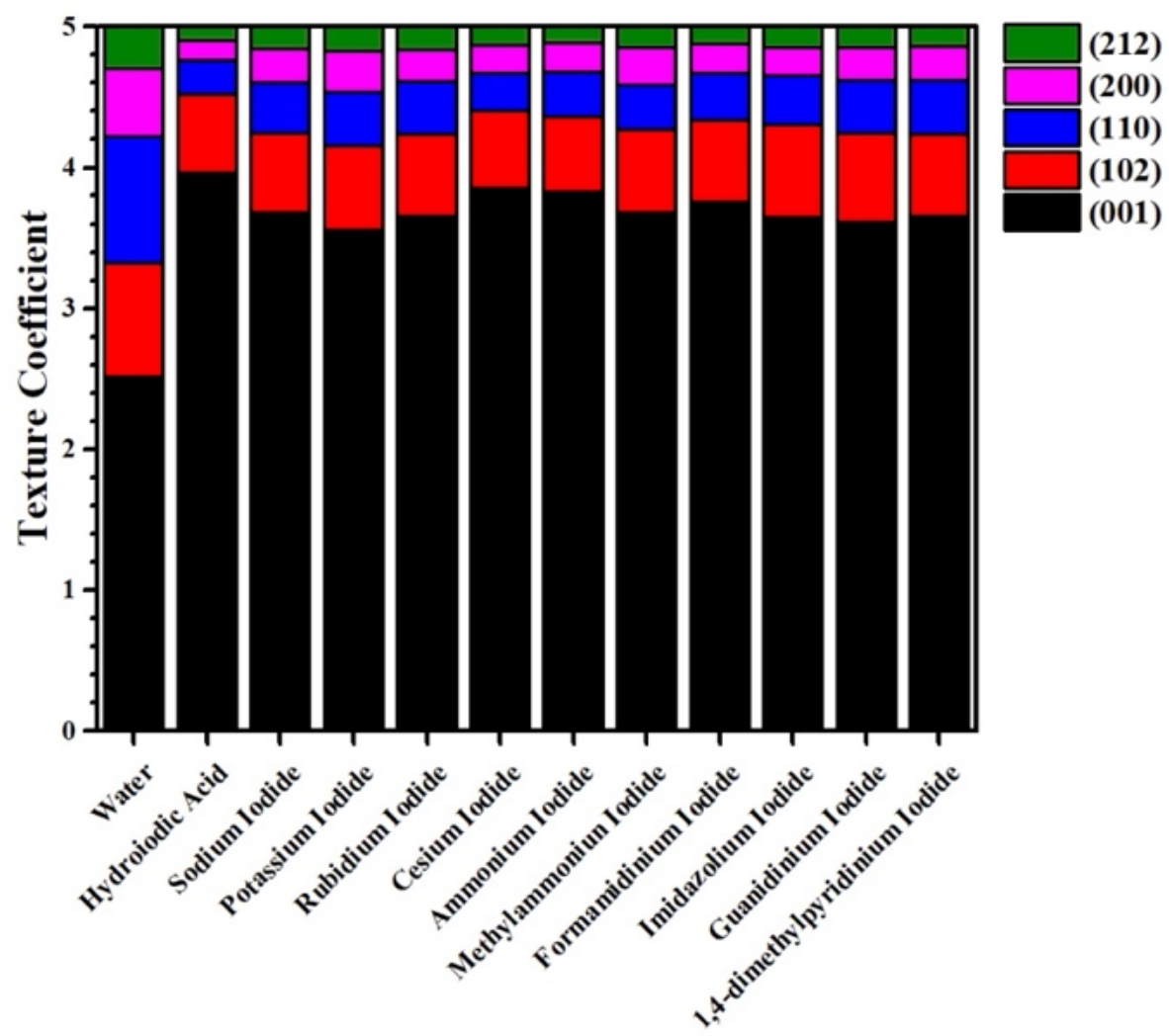

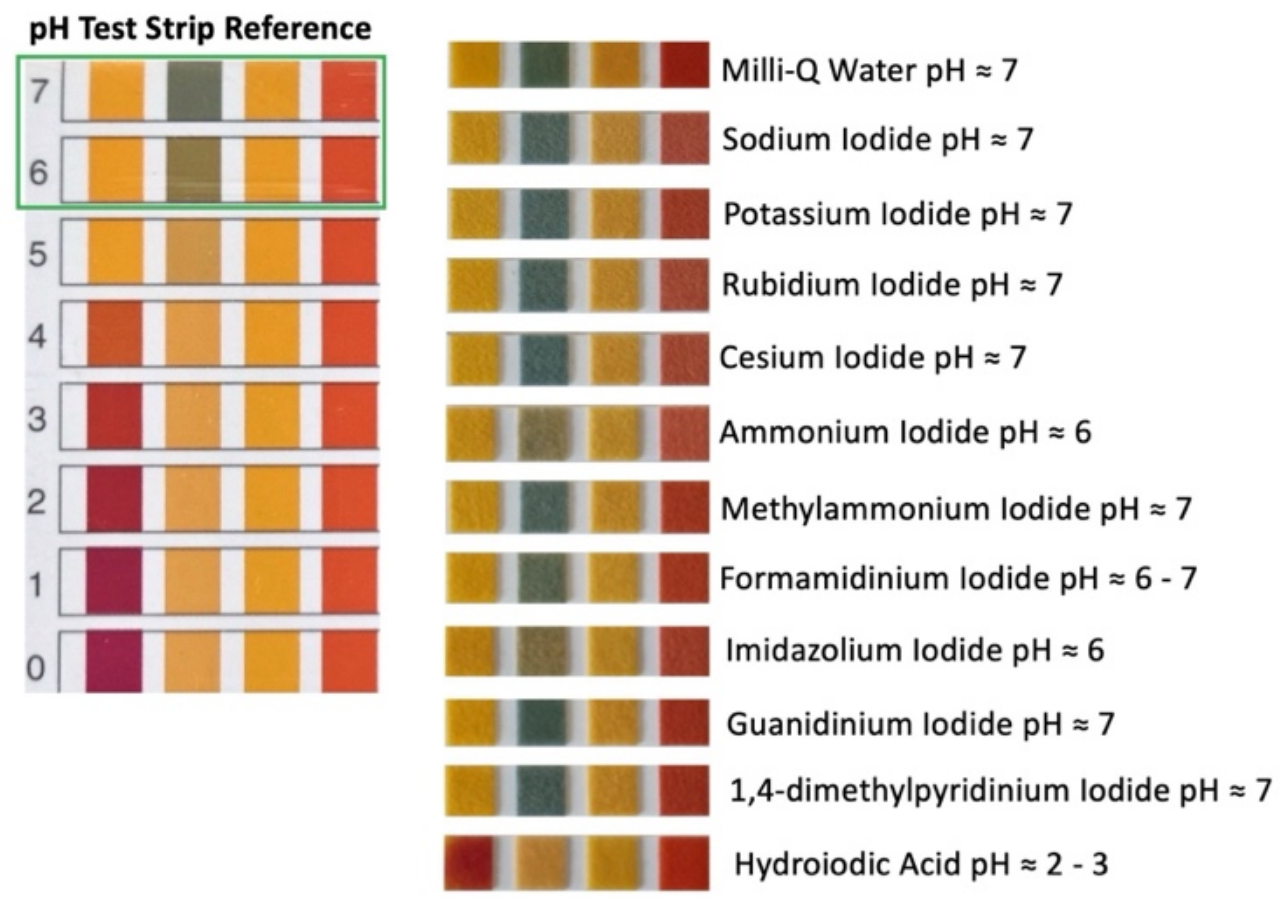

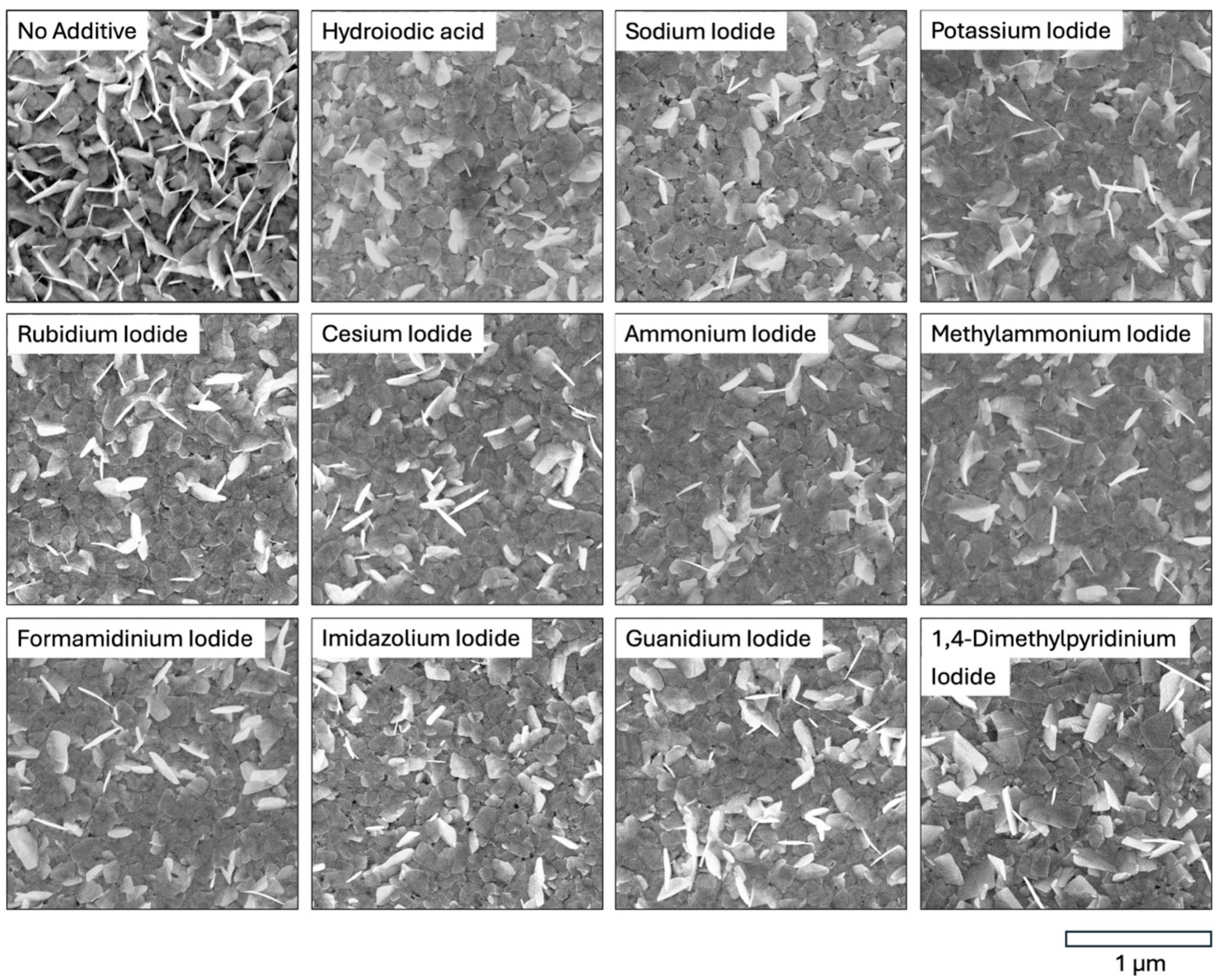

Figure S25: Preliminary experiments with a vast array of iodine-based salts (7mM) (between pH 6-7) further evidencing that the iodide ion is the dominant species related to BiOI crystal orientation.

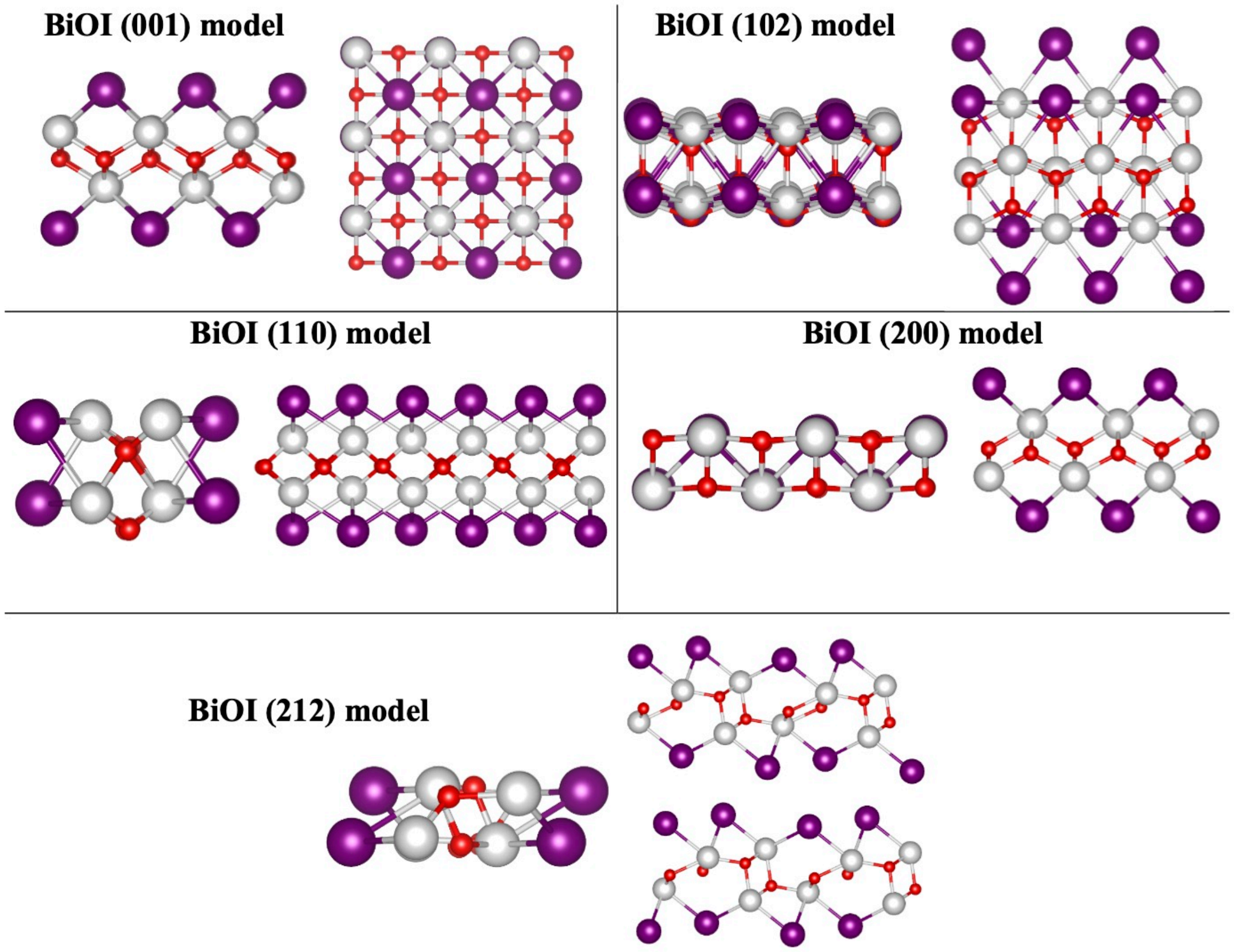

Figure S26: Side and top views of the BiOI (hkl) models used in this study.

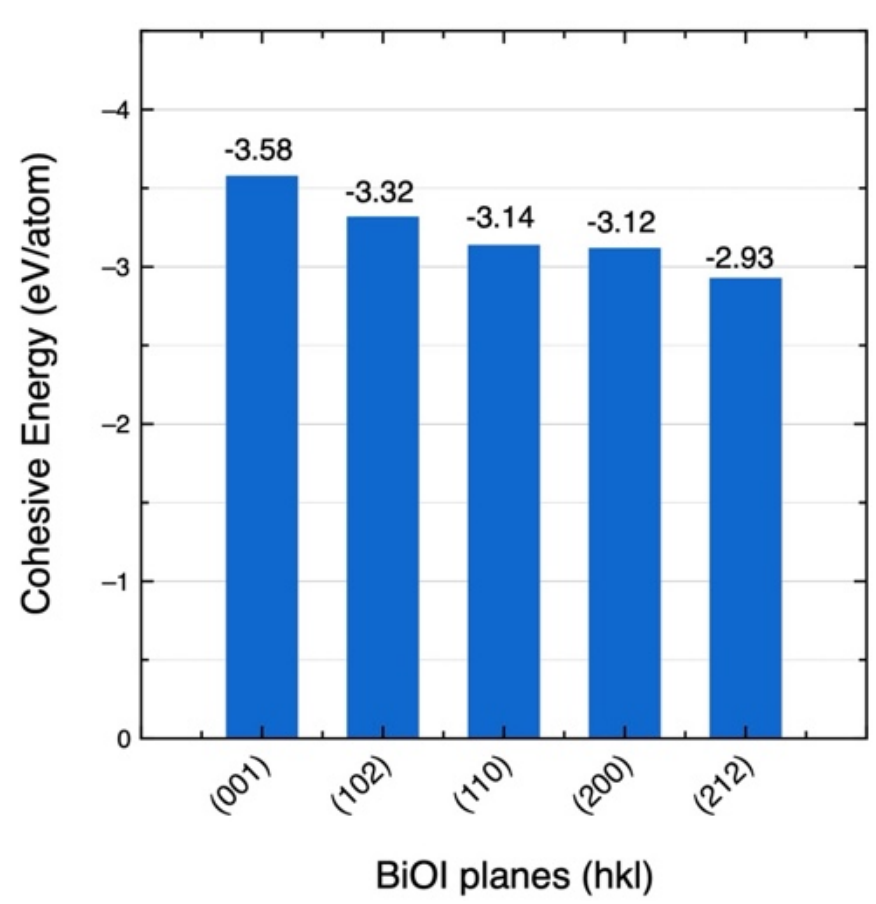

Figure S27: a) Cohesive energy of BiOI (hkl) planes. Planes with higher cohesive energy (i.e. more negative) have higher electronic stability.

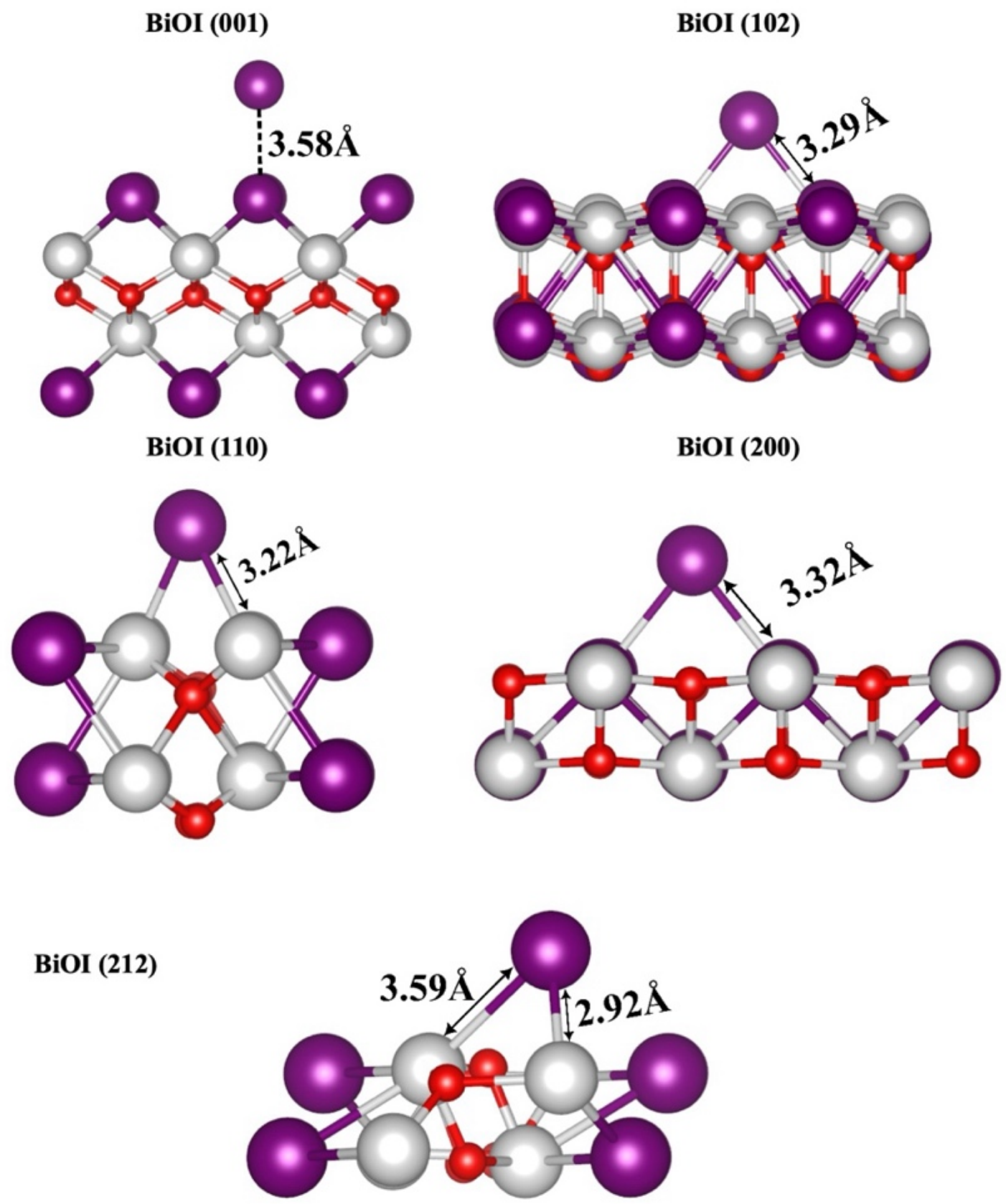

Figure S28: Iodide (I^-) adsorption on model BiOI (hkl) planes. The shortest distances between the BiOI and iodide are illustrated in the images. These models were used to calculate the iodide adsorption energy on different BiOI planes.

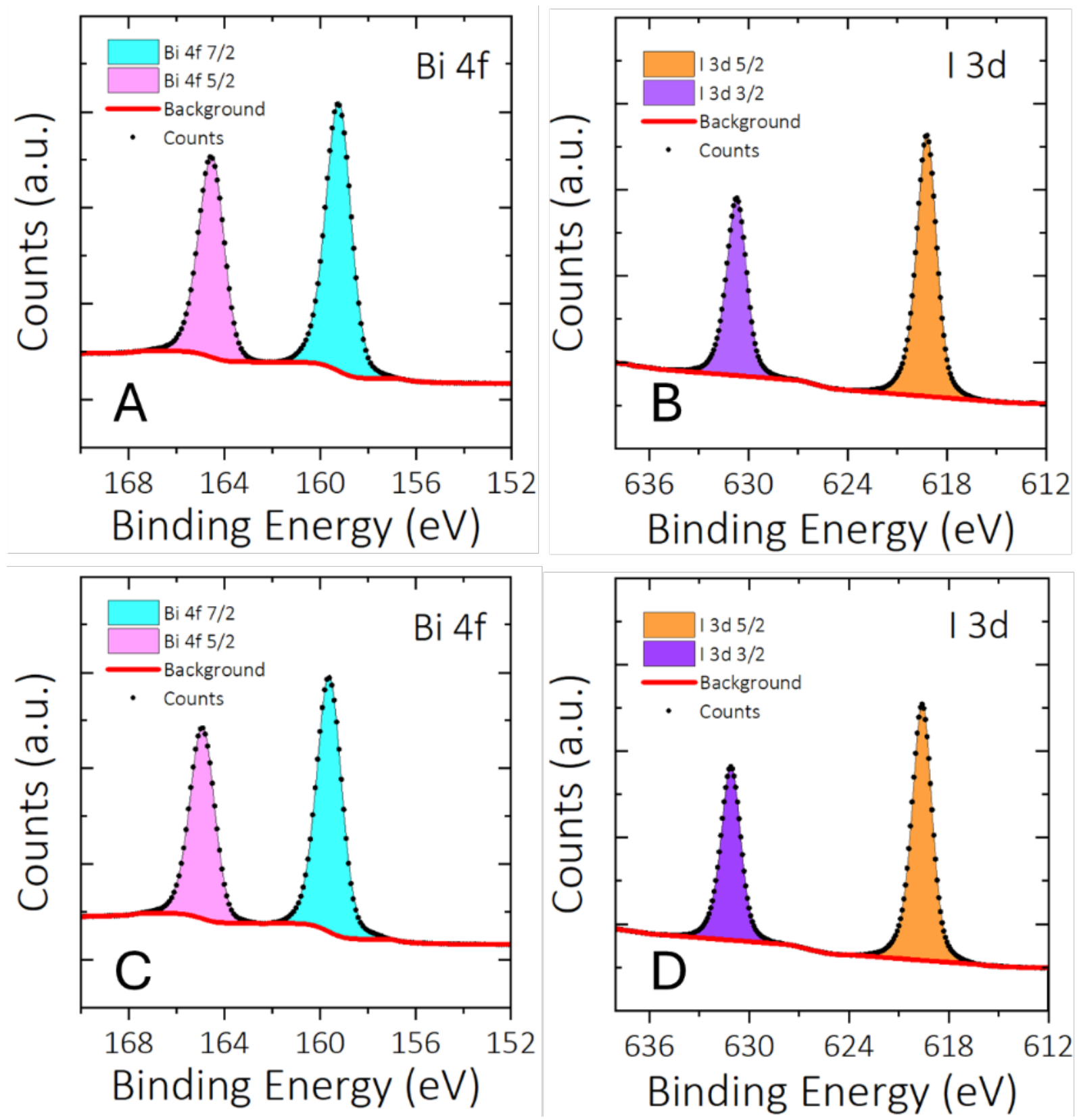

Condition	Bi%	I%	I/Bi Ratio
No Additive	50.43	49.57	0.98
7 mM HI	49.56	50.45	1.02

Figure S29: XPS core level analysis of three-layer 100 mg/mL standard BiOI a) Bi 4f, and b) I 3d spectra, alongside three-layer 100 mg/mL 001-BiOI c) Bi 4f, and d) I 3d spectra. Estimated atomic percentages and I/Bi ratios are included in attached table.

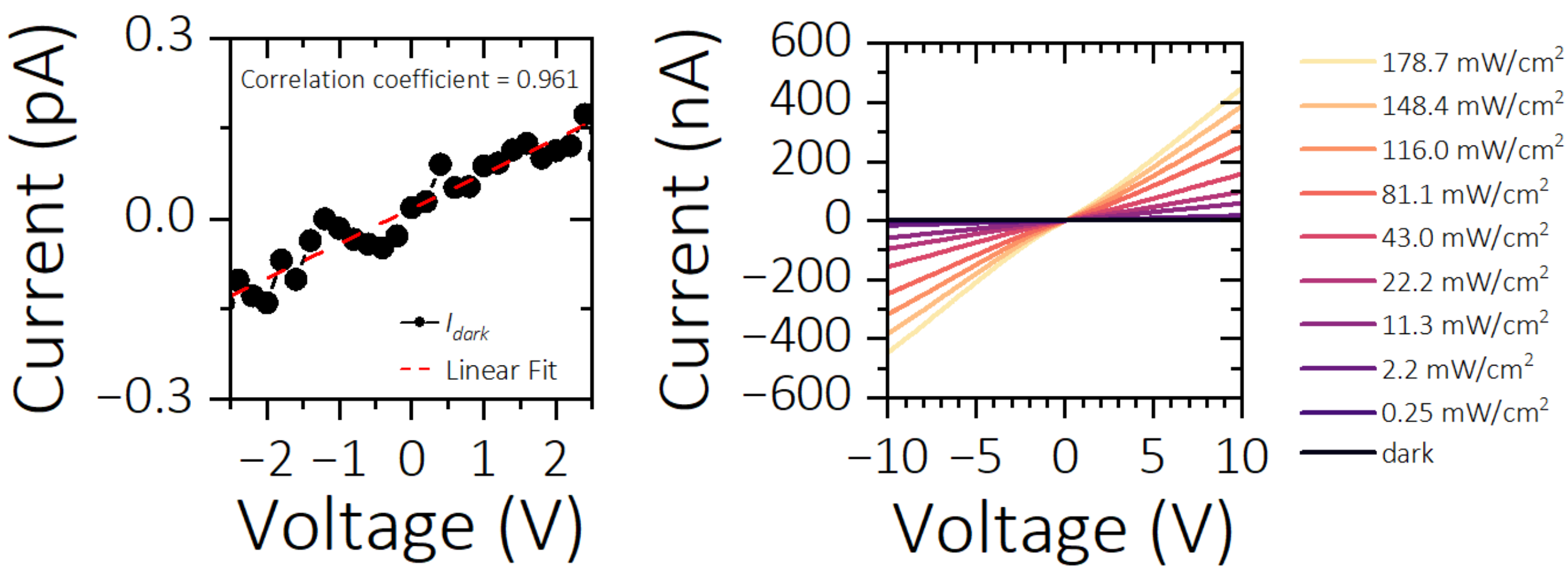

Figure S30: The *I-V* traces of an as-prepared standard 6-layer 50 mg/mL BiOI PD, measured under dark and varying irradiance conditions (455 nm light).

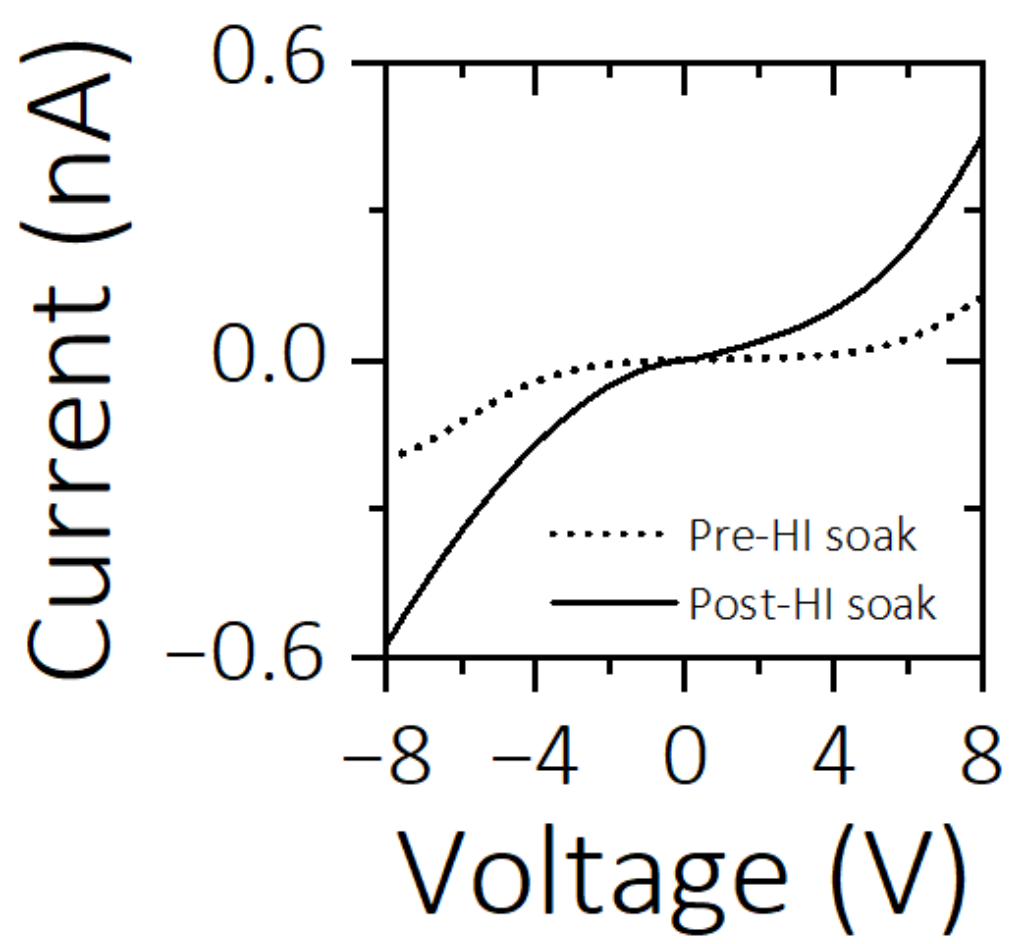

Figure S31: Dark *I-V* sweep of standard BiOI PD, before and after 1mM HI soak treatment (30 minutes).

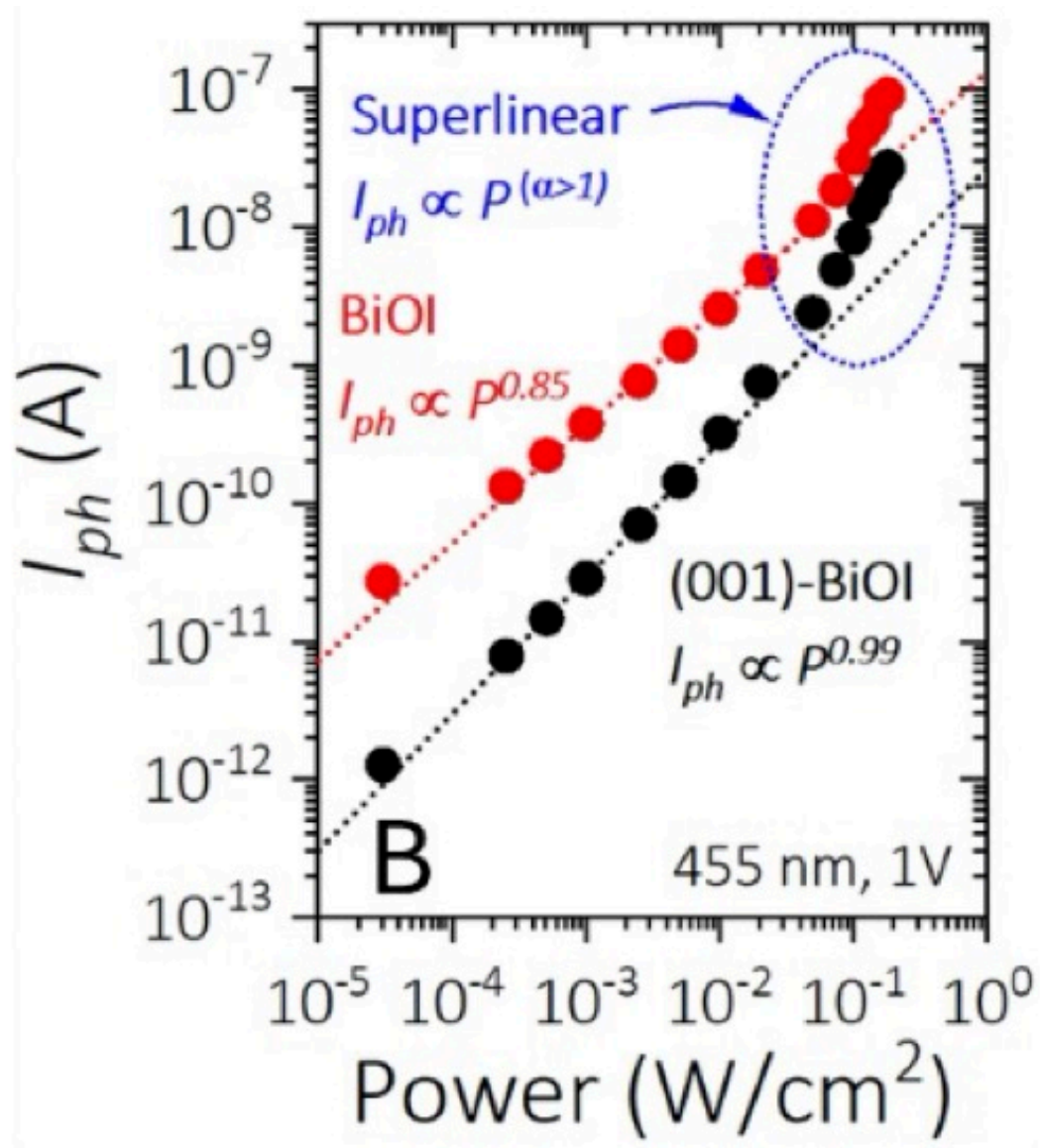

Figure S32: Power-law relationship trends of r-BiOI and 001-BiOI PDs (2L, 150 mg/mL) upon initial pristine first measurement. The power law exponent measured near unity (α=0.99) for pristine as prepared 001-BiOI PDs (prior to light soaking) under low light (<10 mW/cm^2). At irradiance levels exceeding ~10 mW/cm^2, the power-law relationship deviates, with the exponent α exceeding unity (superlinear response), which is the case for both PD types. This power-dependent superlinearity may be attributed to a trap-filling phenomena. Interestingly, after a short period of light-soaking to steady-state (constant I_{light}) the superlinear component was removed and the power law exponents change and stabilize to α=0.70 (r-BiOI) and α=0.89 (001-BiOI) as shown in the manuscript Figure 5.

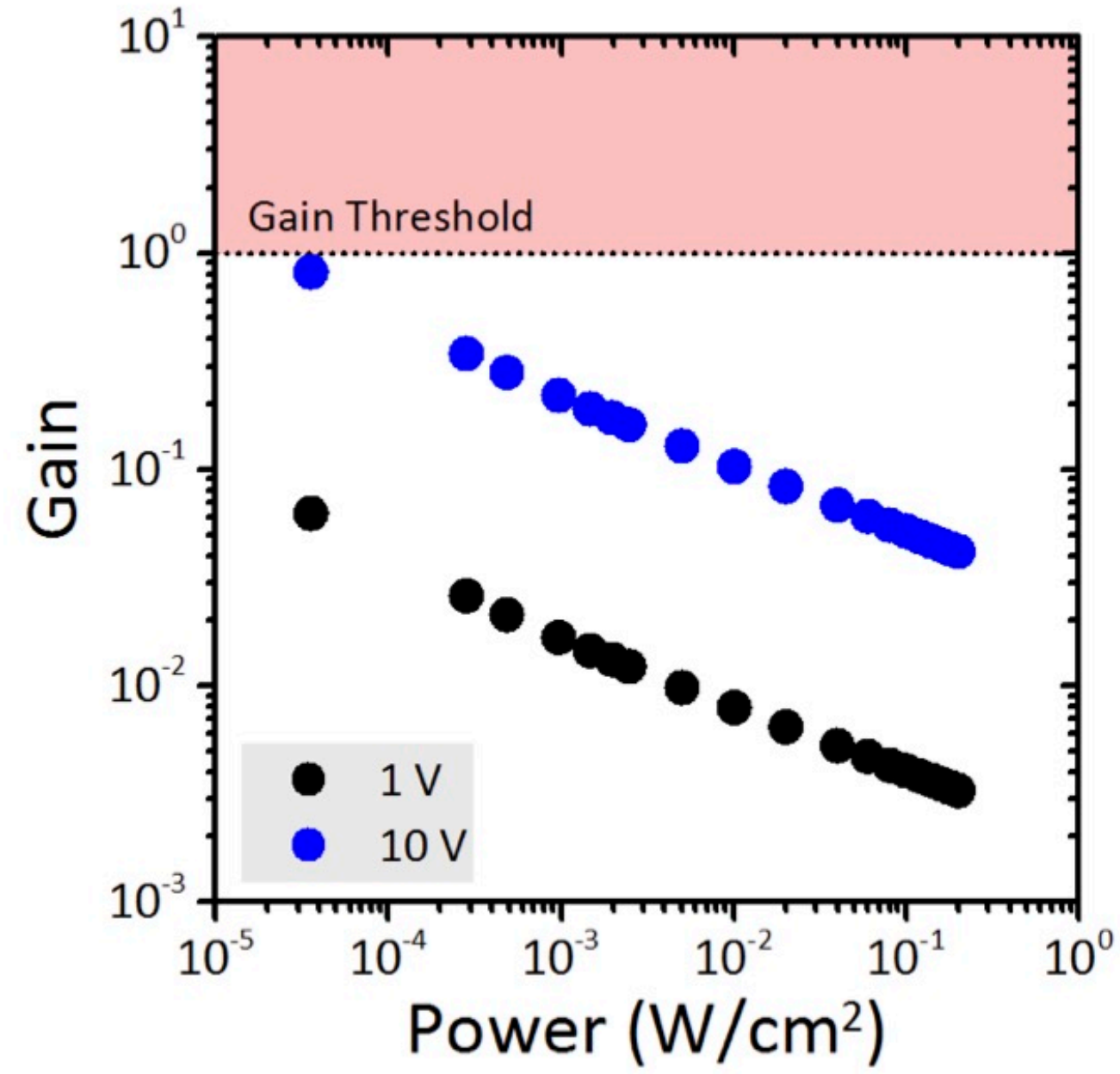

Figure S33: Calculation of photogain in our BiOI photodetectors. Photogain can be defined as follows

$$G = \frac{Rhc}{q\lambda} = \frac{\tau_{eff}}{\tau_{tr}} = \frac{k_t}{k_d \tau_{tr}} = \frac{\tau_{eff}}{L^2}$$

where h, c, λ, τ_{eff}, τ_{tr}, k_t, k_d, L, μ, and V represent the Planck constant, speed of light, wavelength of the incident light, effective lifetime of trapped (minority) charge carriers, transit time of (majority) charge carriers, trapping rate, detrapping rate, channel length, majority charge carrier mobility, and voltage bias. Substituting our R values into the above Equation, the photogain (G) of our champion 001-BiOI PD was estimated for both 1 V and 10 V applied biases, over a variety in blue light (455 nm) intensities. Even at the highest bias and lowest illumination the photodetectors have G<1 indicating that the photodetection mechanism is not Gain based. As BiOI is proven to be *n*-type, the minority carriers will consist of photogenerated holes. Therefore, as G is shown to be below the threshold, the assumption can then be made that τ_{eff} of the minority (hole) carrier is far less than the transit time for the majority (electron) carrier to migrate between electrodes. To exceed this threshold, aspects such as the channel spacing or applied voltage could be adjusted or defects introduced to both increase k_t and reduce k_d of the trapped minority carrier – resulting in an improved photogain. As we have established that during operation there is no net Gain (even at 10V bias) the trap states are predominantly acting as recombination centres rather than contributing to photoconductive Gain (photon recycling).

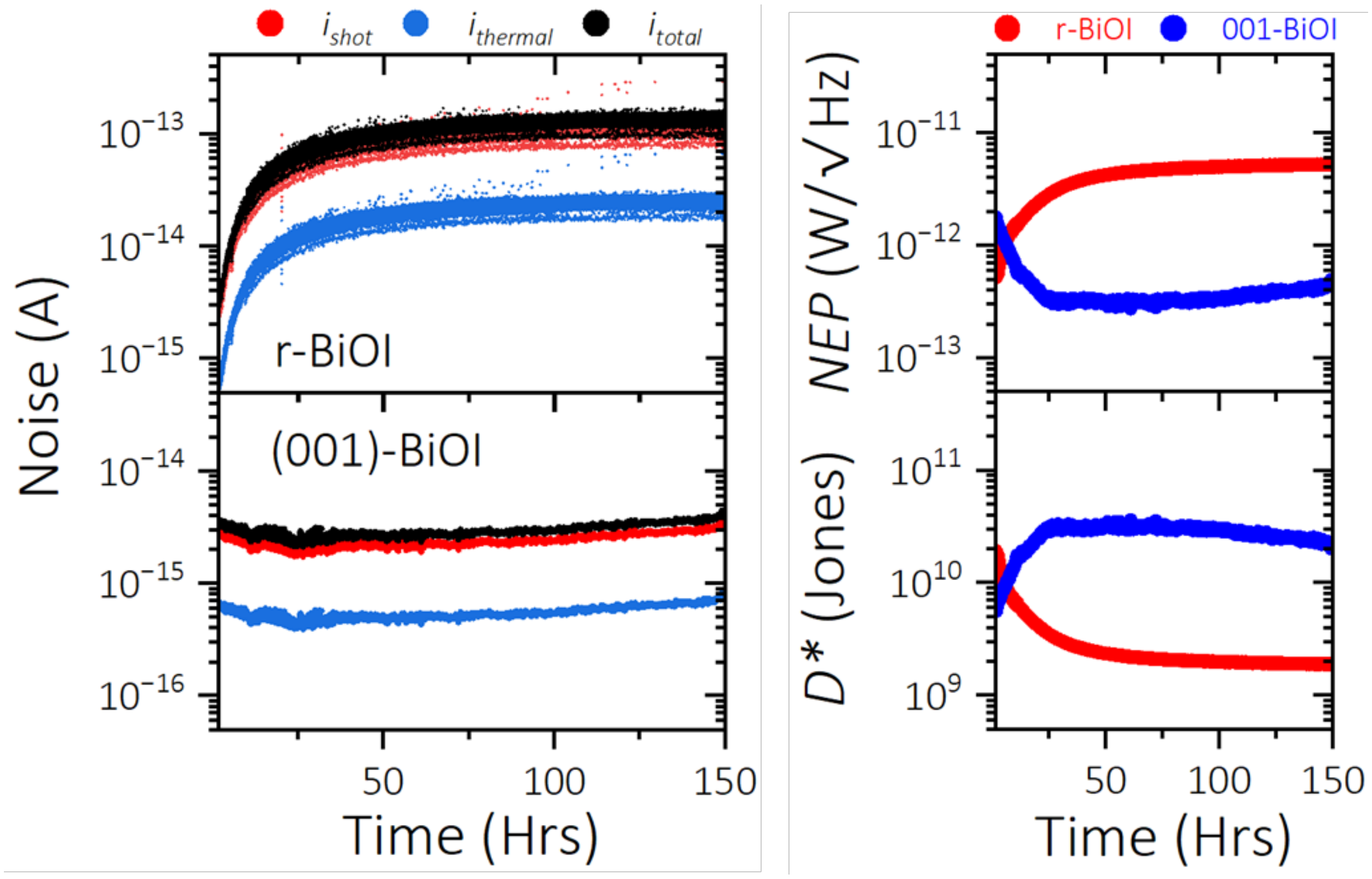

Figure S34: (Left) Approximated noise currents and (Right) NEP and *D** of r-BiOI and 001-BiOI PDs, measured from 200 mW/cm^2 irradiance (λ=455 mn) at 1V bias.

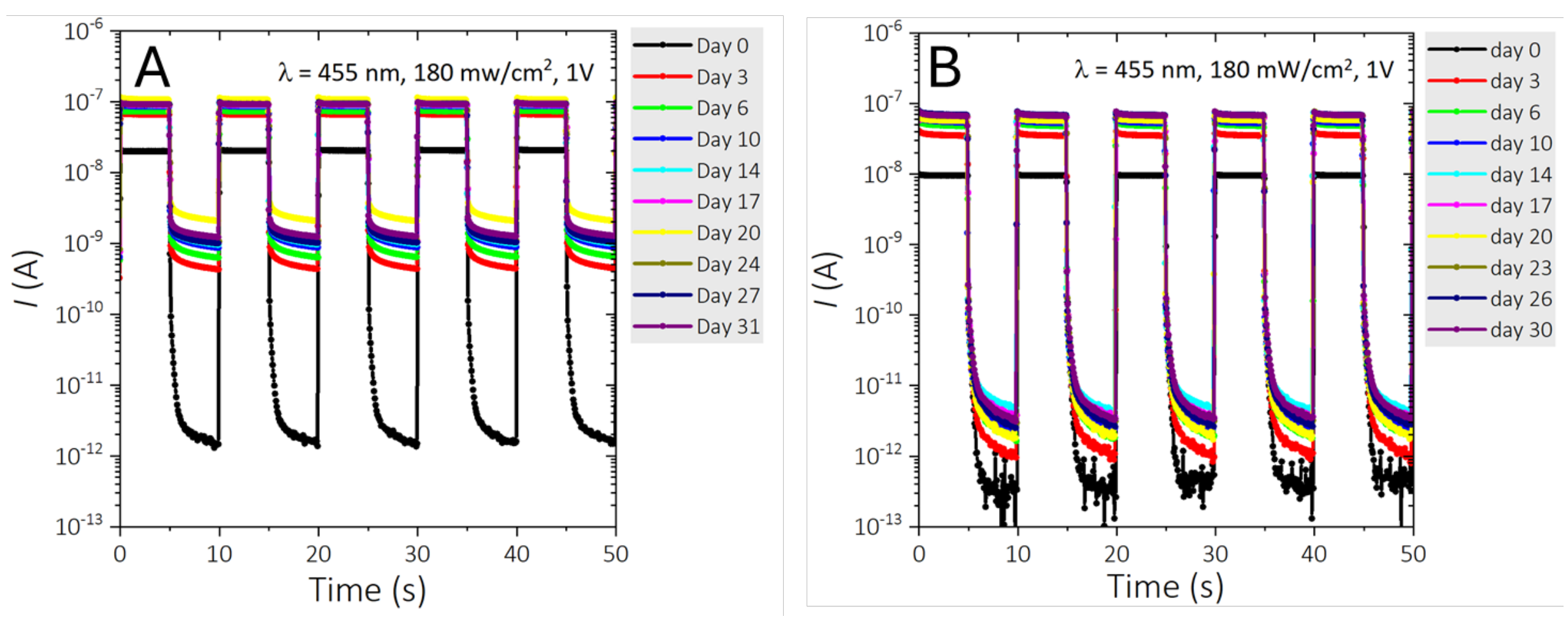

Figure S35: Transient *I-t* measurements of a) r-BiOI, and b) 001-BiOI PDs (two-layers, 150 mg/mL), obtained over a month-long period under high power (180 mW/cm^2) illumination.

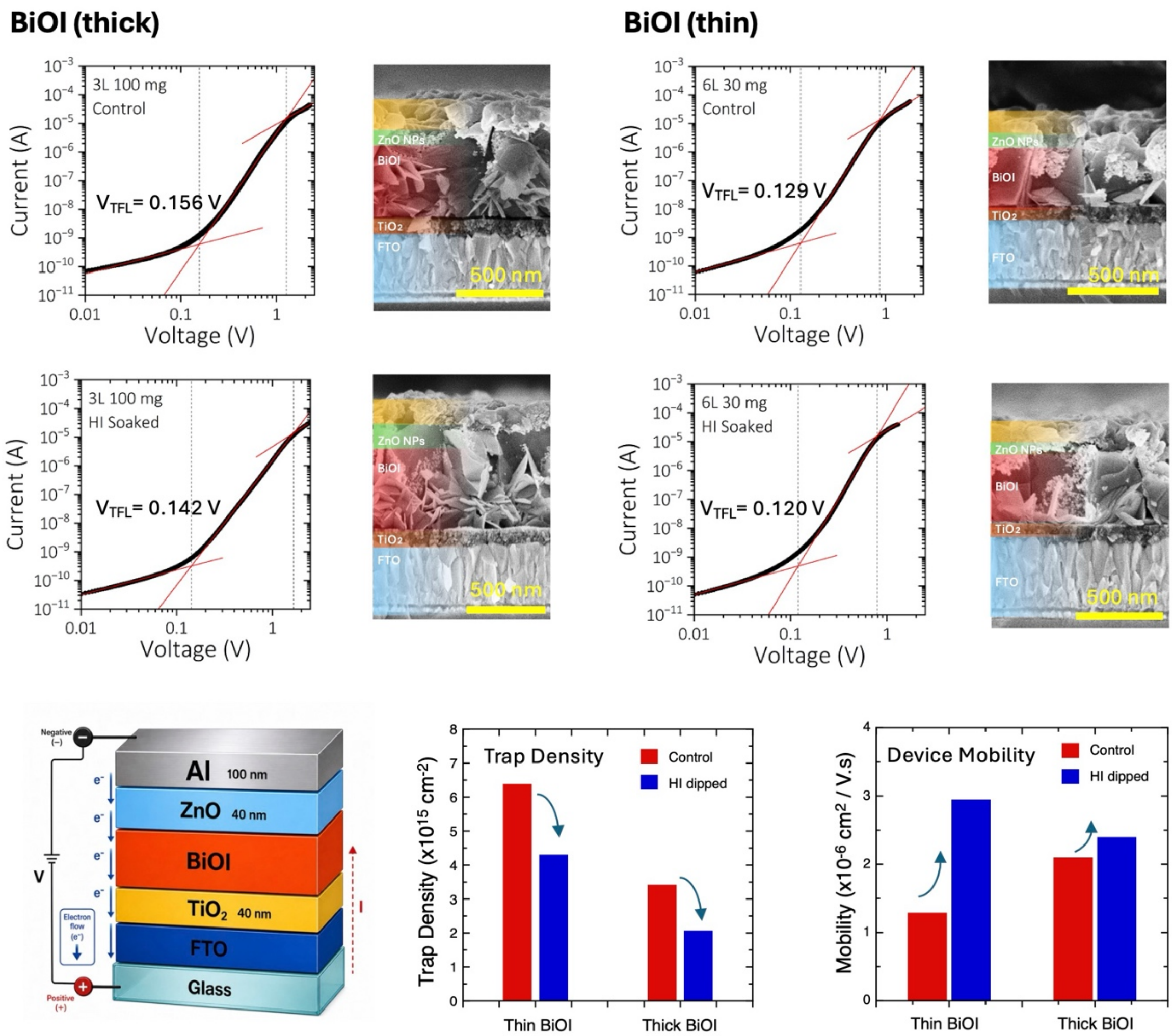

Figure S36: Space-charge-limited current (SCLC) analysis of control and HI-treated randomly oriented (r-BiOI) electron-only devices with thickness-corrected trap-density calculations for thin and thick BiOI films. Here we used a relative dielectric constant for BiOI $\varepsilon(r)$=47.62.[17] Log(I)–log(V) characteristics showing the ohmic, trap-filling, and Mott–Gurney transport regimes for thick (~490–602 nm) and thin (~326–383 nm) BiOI films before and after HI treatment alongside cross section SEM images of the resultant devices.

For the thick BiOI films, the control and HI-treated devices exhibited trap-filling slopes of 5.13 and 4.56, respectively, with comparable Mott–Gurney slopes of 1.99 and 2.00. HI treatment reduced the trap density from 3.42×10^{15}/cm^3 to 2.07×10^{15}/cm^3 (40% decrease) and increased the mobility from (2.1×10^{-6} cm^2/Vs) to (2.39×10^{-6} cm^2/Vs. Although the trap-filling region broadened from 1.12V to 1.53V following treatment, the simultaneous reduction in trap-filled-limit voltage (V_{TFL}), lower trap density, and improved mobility indicate that iodine incorporation improves the electronic quality of the films. The thin BiOI control and HI-treated films exhibited ohmic slopes of 1.00 and 0.99, trap-filling slopes of 5.17 and 5.97, and Mott–Gurney slopes of 2.00 and 2.01, respectively. HI treatment reduced V_{TFL} from 0.129 to 0.120 V, resulting in a reduction in trap density from 6.39×10^{15}/cm^3 to 4.31×10^{15}/cm^3 (33% decrease) and a corresponding increase in mobility from 1.29×10^{-6} cm^2/Vs to 2.95×10^{-6} cm^2/Vs. The trap-filling regime

also narrowed slightly from 0.74V to 0.67 V and became steeper, indicating a more abrupt trap-filling process and a less electronically disruptive trap landscape following iodine treatment.

Collectively, these data demonstrate that HI treatment systematically reduces the density of electronically active trap states while improving carrier transport. The effect is more pronounced in the thinner films, where trap reduction, mobility enhancement, steeper trap-filling behaviour, and a narrower trap-filling regime all point towards partial passivation of electrically active iodine-vacancy-related defects and a more ordered defect landscape.

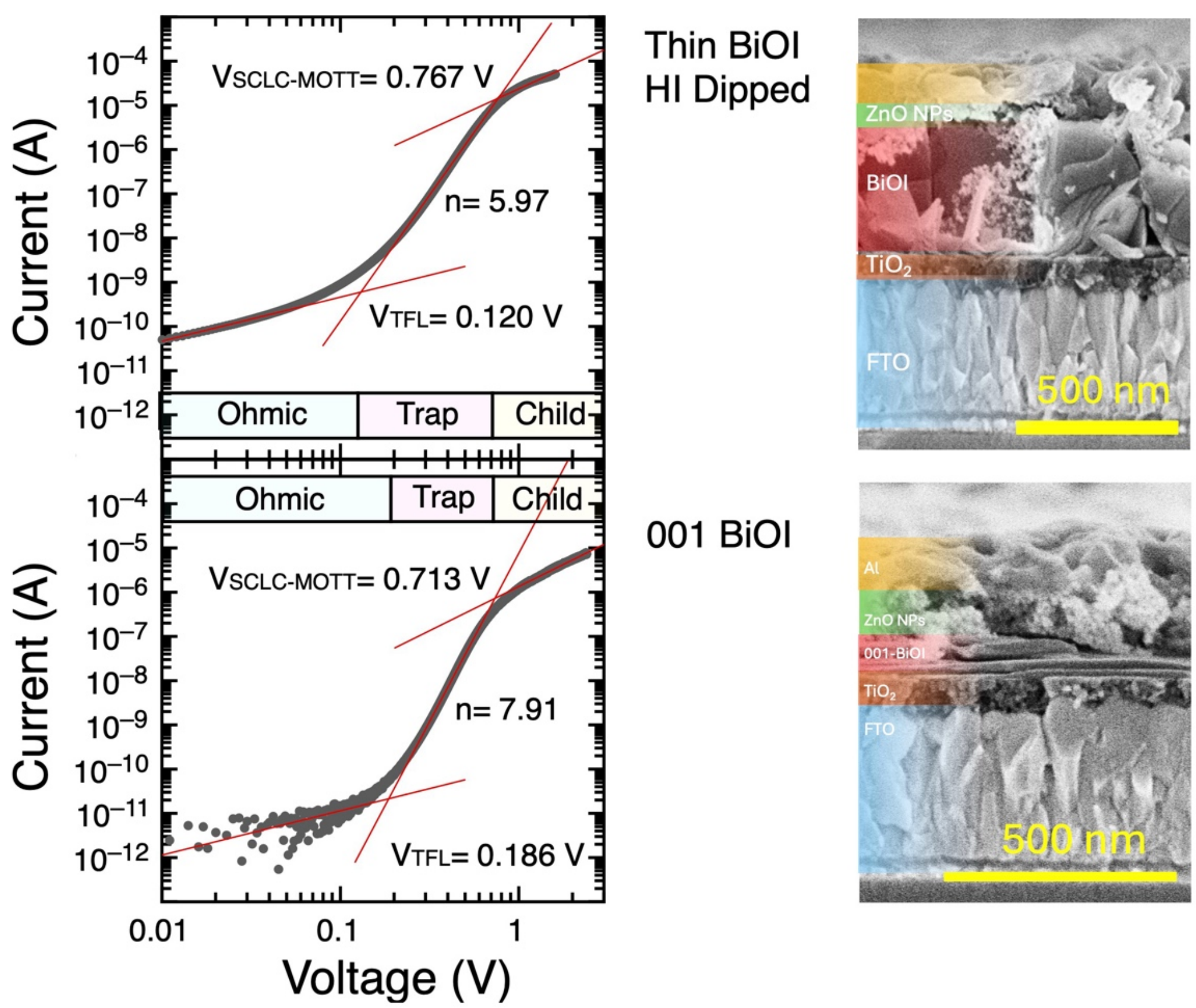

Figure S37: Space-charge-limited current (SCLC) analysis comparing HI-treated r-BiOI (~383 nm) and highly oriented 001-BiOI (~90 nm) electron-only devices. Log(I)–log(V) characteristics with fitted ohmic, trap-filling, and Mott–Gurney (child) regions. The HI-treated r-BiOI film exhibited an ohmic slope of 0.99, trap-filling slope of 5.97, and Mott slope of 2.01, yielding a trap-filled-limit voltage (V_{TFL}) of 0.120 V, trap density of $4.31\times10^{15}/cm^3$, and mobility of 2.95×10^{-6} cm^2/Vs. In contrast, the 001-BiOI film exhibited an ohmic slope of 0.96, a markedly sharper trap-filling transition (slope = 7.87), and an ideal Mott–Gurney slope of 2.00. Conventional SCLC analysis yielded an apparent trap density of $8.97\times10^{16}/cm^3$ and an effective mobility of 2.06×10^{-9} cm^2/Vs, approximately 3 orders of magnitude lower than the HI-treated r-BiOI film.

The 001-BiOI device also exhibited a narrower trap-filling regime (0.527 V versus 0.647 V) and a steeper trap-filling slope (n = 7.87 versus 5.97) than the HI-treated r-BiOI film, indicating a more abrupt trap-filling process and a more well-defined trap population. Conversely, the broader trap-filling region and shallower trap-filling slope observed in r-BiOI are consistent with a wider energetic distribution of electronically active trap states. While the sharper trap-filling transition indicates a more well-defined trap-filling process in 001-BiOI, the elevated (V_{TFL}) and dramatically reduced effective mobility are attributed, at least in part, to strongly anisotropic transport through the layered BiOI structure, where carriers traverse van der Waals gaps perpendicular to the (001) planes. These results indicate that iodine incorporation reduces the density of electronically active trap states, whereas crystallographic orientation independently exerts a dominant influence on charge transport behaviour in BiOI. Corresponding cross section SEM images of the devices are also provided for clarity.

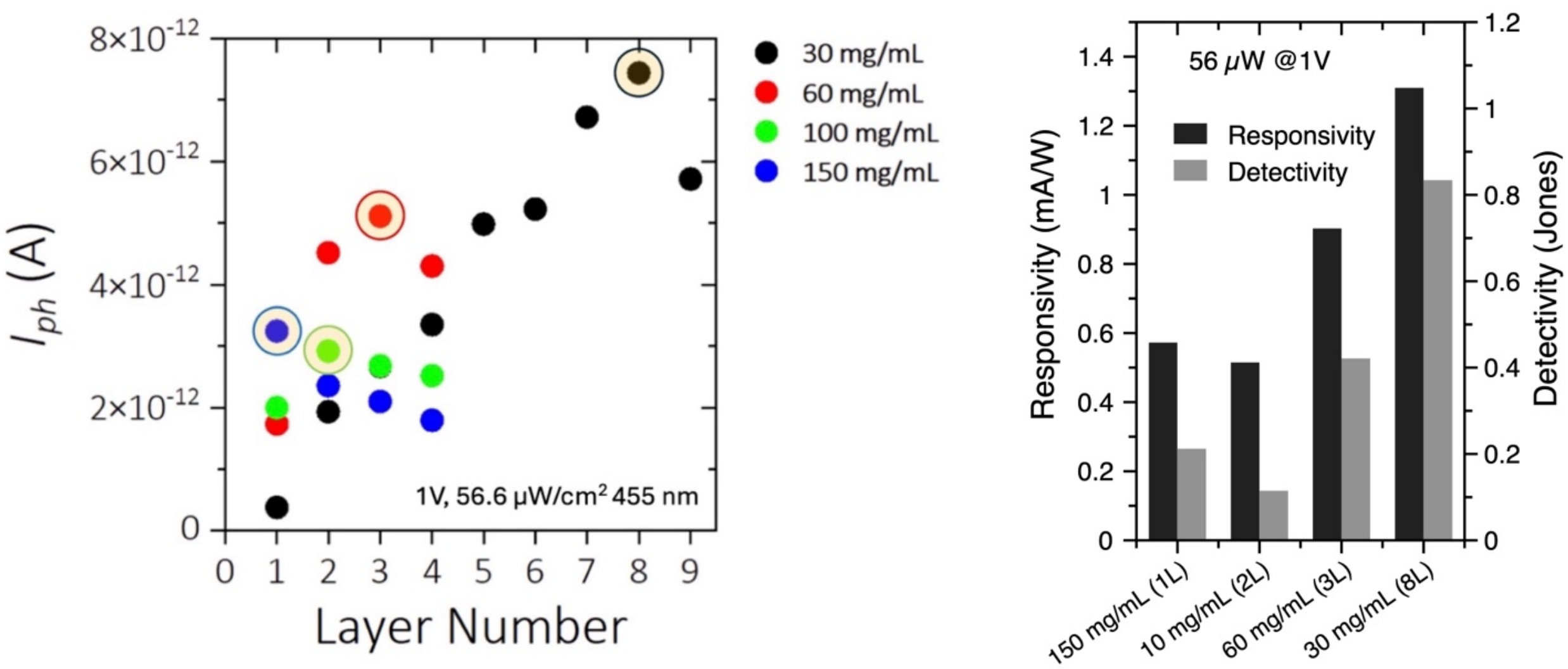

Figure S38: Preliminary device optimization. (left) The measured photocurrents of different concentration 001-BiOI PDs at low power (~56.6μW/cm^2) with sequentially increasing layer count. The maximum photocurrent occurs at lower layer numbers for increased template thicknesses (precursor concentrations) as highlighted by the larger circles. (right) Responsivity and Detectivity as a function of template deposition parameters highlighting that the best-performing device fabricated with eight layers (8L) of a 30 mg/mL precursor solution

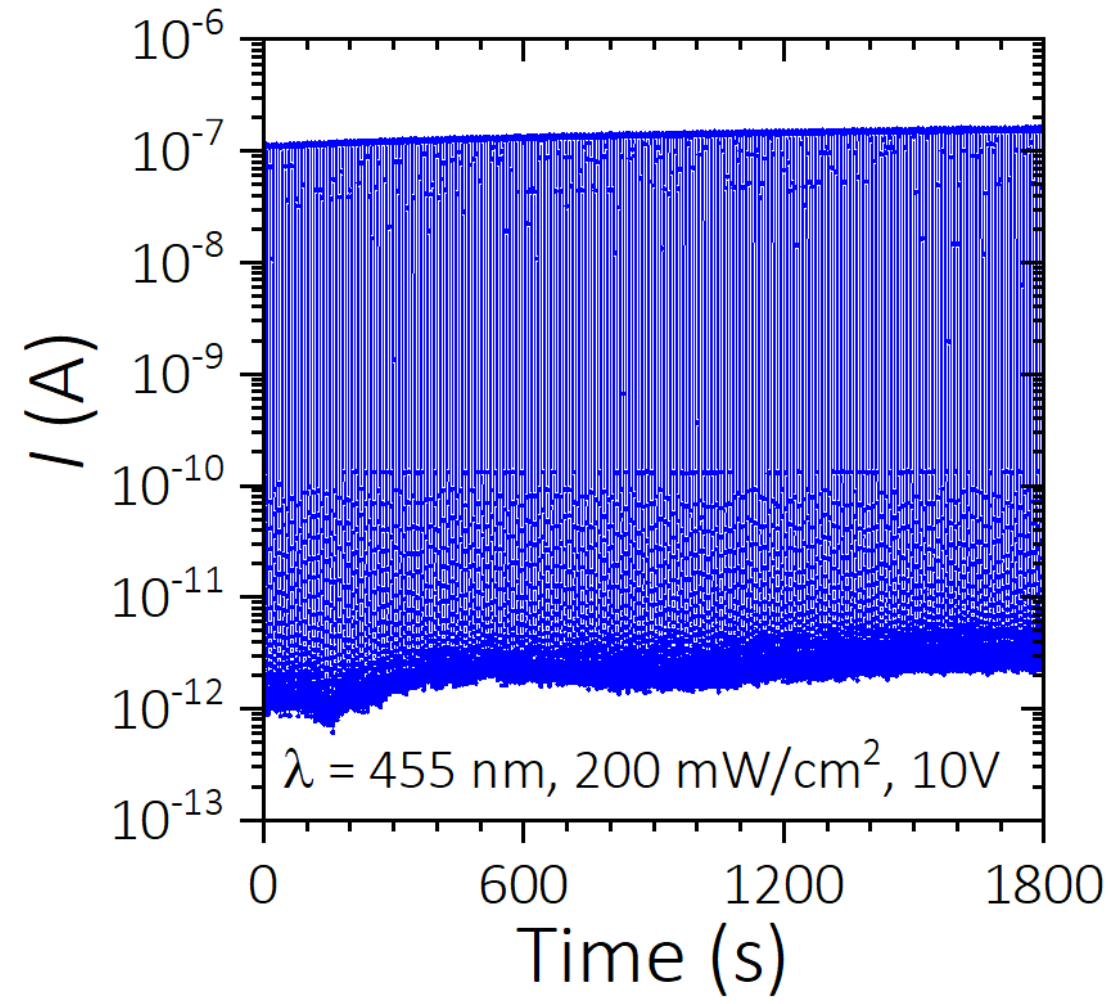

Figure S39: Measured *I-t* response of champion (001)-BiOI PD, illumination pulsed (5s ON, 5s OFF) over 30 mins.

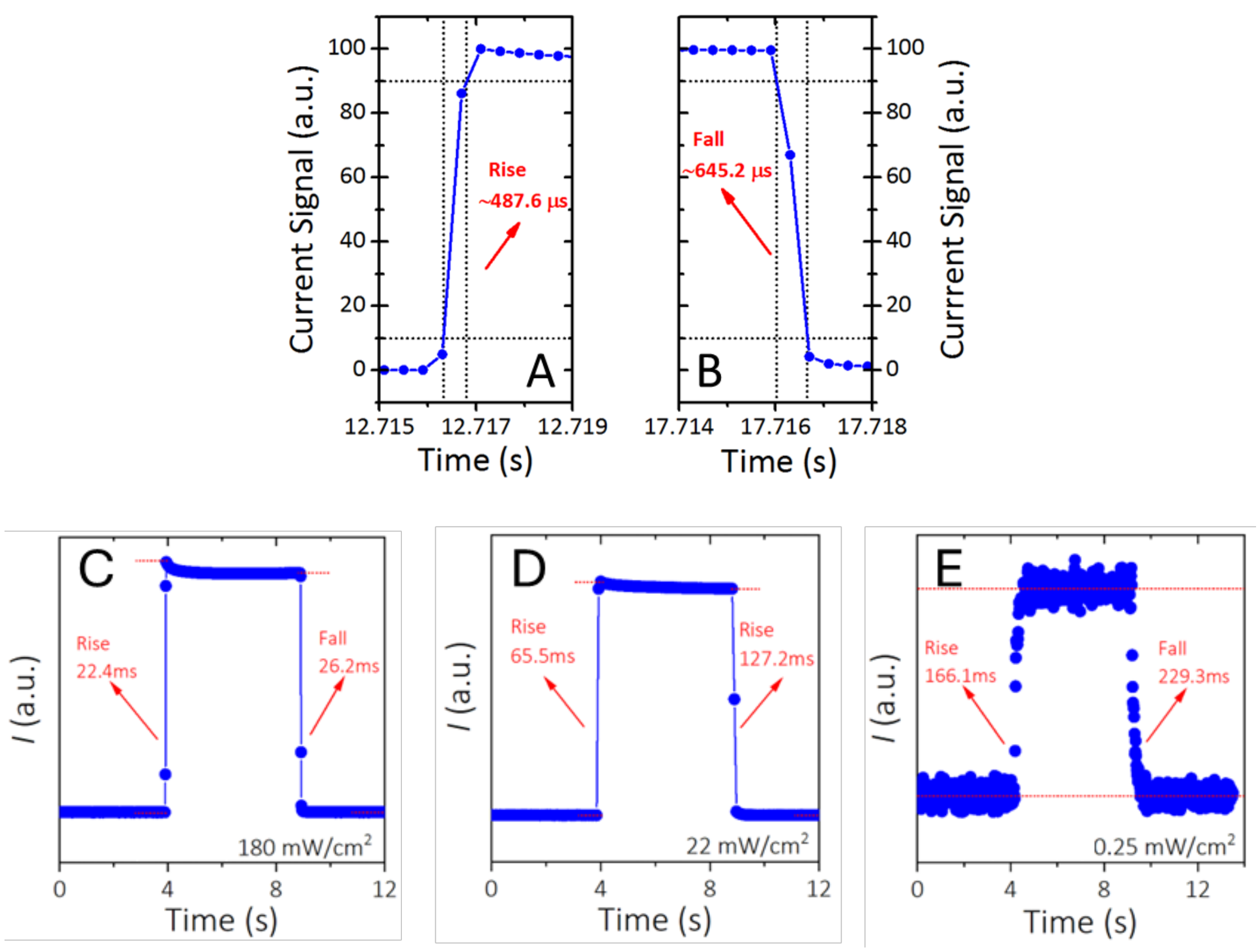

Figure S40: Temporal analysis of champion 001-BiOI PD, measured at high intensity (200 mW/cm^2) blue light (455 nm) at 10 V bias, showing a) the rise time, and b) the fall time. Temporal analysis of typical 001-BiOI PD rise-fall times, measured at c) high (180 mW/cm^2), d) intermediate (22 mW/cm^2), and e) low (0.25 mW/cm^2) intensity blue light (455 nm) at 1 V bias.

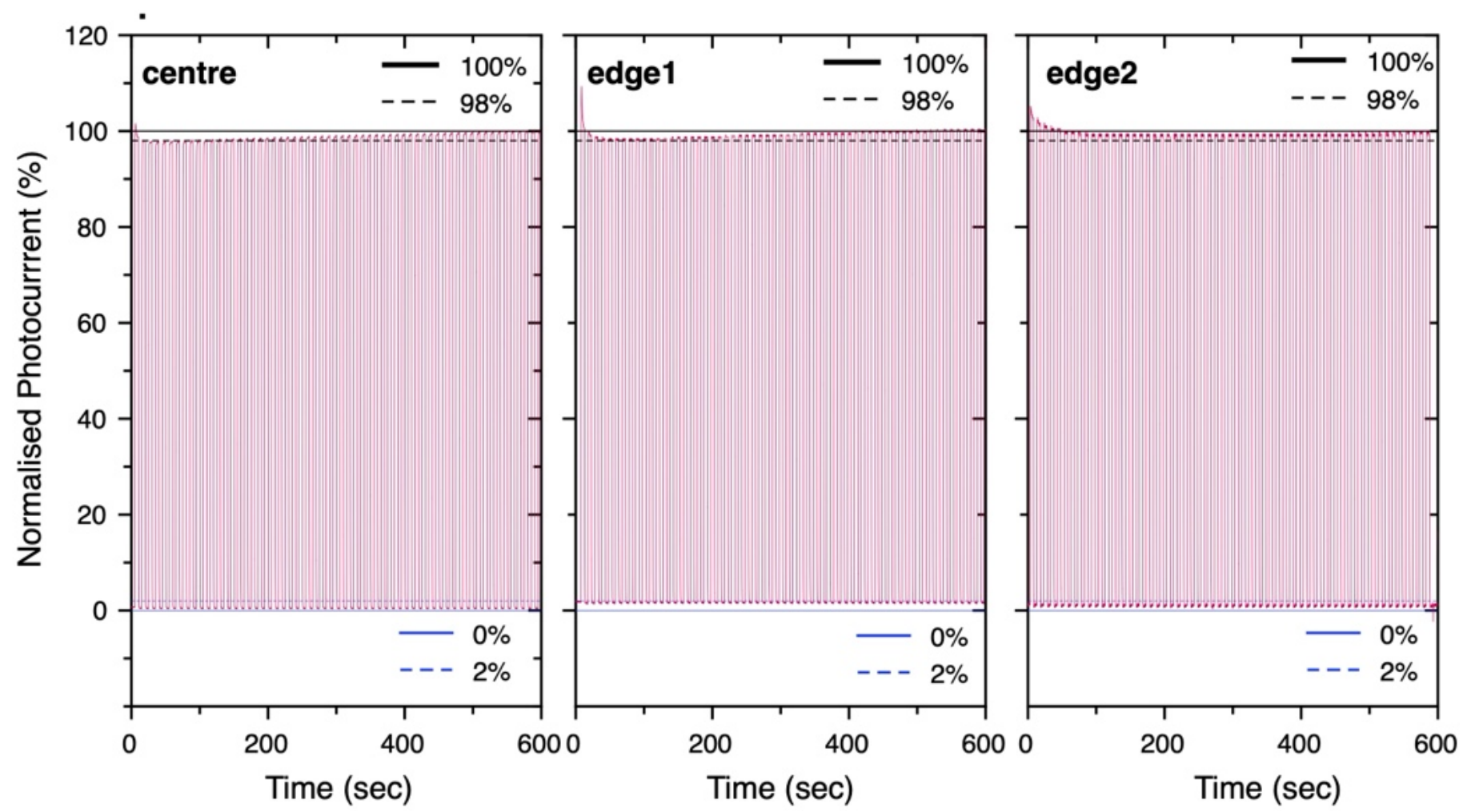

Figure S41: The temporal stability of single pixel wafer BiOI devices with top-down evaporated Au electrodes, corresponding to wafer position (λ = 455 nm, 2 V, 180 mW/cm^2). For all wafer positions, the pixel devices exhibited superb photocurrent stability within 2% margins.

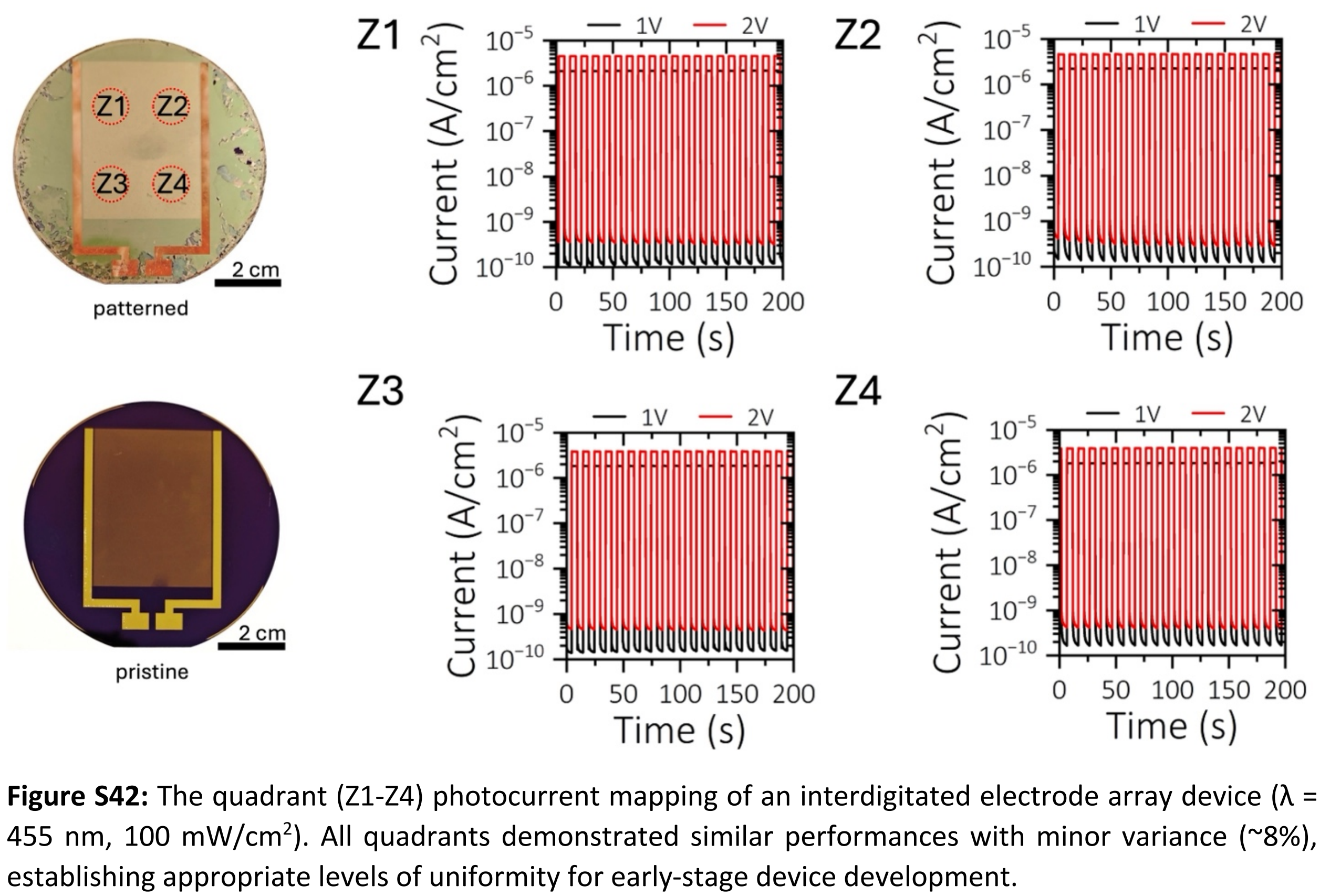

Figure S42: The quadrant (Z1-Z4) photocurrent mapping of an interdigitated electrode array device (λ = 455 nm, 100 mW/cm^2). All quadrants demonstrated similar performances with minor variance (~8%), establishing appropriate levels of uniformity for early-stage device development.

References:

1. Chen, W., et al., *Cesium Doped NiOx as an Efficient Hole Extraction Layer for Inverted Planar Perovskite Solar Cells.* Advanced Energy Materials, 2017. **7**(19): p. 1700722.
2. Zhang, C., et al., *Influence of different TiO2 blocking films on the photovoltaic performance of perovskite solar cells.* Applied Surface Science, 2016. **388**: p. 82–88.
3. Li, M., et al., *Microsized BiOCl Square Nanosheets as Ultraviolet Photodetectors and Photocatalysts.* ACS Applied Materials & Interfaces, 2016. **8**(10): p. 6662–6668.
4. Chen, L., C. Yang, and C. Yan, *High-performance UV detectors based on 2D CVD bismuth oxybromide single-crystal nanosheets.* Journal of Materials Science & Technology, 2020. **48**: p. 100–104.
5. Liu, P., et al., *Controllable preparation of ultrathin 2D BiOBr crystals for high-performance ultraviolet photodetector.* Science China Materials, 2021. **64**(1): p. 189–197.
6. Prasad, M.D., M.G. Krishna, and S.K. Batabyal, *Facet-Engineered Surfaces of Two-Dimensional Layered BiOI and Au–BiOI Substrates for Tuning the Surface-Enhanced Raman Scattering and Visible Light Photodetector Response.* ACS Applied Nano Materials, 2019. **2**(6): p. 3906–3915.
7. Sun, Z., Y. Wang, and B. Mei, *Highly sensitive UV photodetector based on solution-processed bismuth oxyiodide epitaxial thin films.* Journal of Materials Chemistry C, 2023. **11**(11): p. 3805–3811.
8. Zeng, W., et al., *Synthesis of Large-Area Atomically Thin BiOI Crystals with Highly Sensitive and Controllable Photodetection.* Advanced Functional Materials, 2019. **29**(16): p. 1900129.
9. Li, Y., et al., *Synthesis of large-area high quality 2D BiOI for highly sensitive ultraviolet photodetection.* Journal of Materials Chemistry C, 2024. **12**(19): p. 6826–6830.
10. Hussain, A.A., A.K. Rana, and M. Ranjan, *Air-stable lead-free hybrid perovskite employing self-powered photodetection with an electron/hole-conductor-free device geometry.* Nanoscale, 2019. **11**(3): p. 1217–1227.
11. Vuong, V.-H., et al., *Engineering Chemical Vapor Deposition for Lead-Free Perovskite-Inspired MA3Bi2I9 Self-Powered Photodetectors with High Performance and Stability.* Advanced Optical Materials, 2021. **9**(12): p. 2100192.
12. Zhang, Z.Y. and G.P. Wang, *High-Anisotropic Flexible Photodetector Based on Centimeter-Sized Cs3Bi2I9 Single-Crystal Sheet.* Advanced Optical Materials, 2025. **13**(27): p. e01263.
13. Li, W.-G., et al., *Enhanced On–Off Ratio Photodetectors Based on Lead-Free Cs3Bi2I9 Single Crystal Thin Films.* Advanced Functional Materials, 2020. **30**(12): p. 1909701.
14. Tailor, N.K., et al., *Polaron-Mediated Photoconduction in Lead-Free Single-Crystalline Perovskite Thin-Film Devices.* The Journal of Physical Chemistry C, 2022. **126**(27): p. 11165–11173.
15. Li, Y., et al., *Ultrastable Lead-Free Double Perovskite Photodetectors with Imaging Capability.* Advanced Materials Interfaces, 2019. **6**(10): p. 1900188.
16. Yang, J., et al., *Stable, High-Sensitivity and Fast-Response Photodetectors Based on Lead-Free Cs2AgBiBr6 Double Perovskite Films.* Advanced Optical Materials, 2019. **7**(13): p. 1801732.
17. Ran, Z., et al., *Bismuth and antimony-based oxyhalides and chalcohalides as potential optoelectronic materials.* npj Computational Materials, 2018. **4**(1): p. 14.
